# Polymorphisms in ERCC4 and ERCC5 and risk of cancers: Systematic research synopsis, meta-analysis, and epidemiological evidence

**DOI:** 10.3389/fonc.2022.951193

**Published:** 2022-08-11

**Authors:** Chunjian Zuo, Xiaolong Lv, Tianyu Liu, Lei Yang, Zelin Yang, Cao Yu, Huanwen Chen

**Affiliations:** ^1^ Department of Thoracic Surgery, Army Medical Center of PLA, Chongqing, China; ^2^ Department of Cardiothoracic Surgery, The First Affiliated Hospital of Chongqing Medical University, Chongqing, China; ^3^ Department of Cardiothoracic Surgery, The Jiang Jin Central Hospital of Chongqing, Chongqing, China

**Keywords:** genetic variant, cancer, susceptibility, epidemiology, ERCC gene

## Abstract

The variants of DNA repair genes have been widely reported to be associated with cancer risk in the past decades. As were two crucial members of nucleotide excision repair pathway, *ERCC4* and *ERCC5* polymorphisms are linked with susceptibility to multiple cancers, but the conclusions were controversial. In this updated meta-analysis concerned with *ERCC4* and *ERCC5* single-nucleotide polymorphisms (SNPs), 160 eligible publications were identified, and we exerted the meta-analysis of correlations between 24 variants and 19 types of cancer. Venice criteria and the false-positive report probability were used to evaluate a cumulative evidence of significant associations. We conducted functional annotations for those strong associations using data from the Encyclopedia of DNA Elements (ENCODE) Project. We obtained 11 polymorphisms significantly related to changed susceptibility to 11 cancers (*p* < 0.05). Strong evidence was assigned to four variant-related cancer risks in Asians (*ERCC4* rs744154 with bladder cancer, *ERCC5* rs2296147 with esophageal cancer, *ERCC5* rs17655 with laryngeal cancer and uterine cancer, and *ERCC5* rs751402 with gastric cancer), moderate to six SNPs with a risk of eight cancers, and weak to nine SNPs with nine cancers. Data from ENCODE and other public databases showed that the loci of these SNPs with strong evidence might fall in putative functional regions. In conclusion, this paper summarizes comprehensive evidence that common variants of *ERCC4* and *ERCC5* genes are strongly associated with the risk of bladder cancer, esophageal cancer, laryngeal cancer, uterine cancer, and gastric cancer and elucidates the crucial role of the DNA repair genes in the genetic predisposition to human cancers.

## Introduction

Cancer has become one of the major and most formidable obstacles to extending human life; the number of newly diagnosed cancer patients and cancer deaths worldwide reached 18.1 million and 9.6 million in 2018 (1). Among the complex array of carcinogenic factors, genetic variants have been shown in many studies to play a crucial role in the pathogenesis of cancer in recent decades (1, 2). Increasing genetic studies are being made to reveal the potential association between genetic polymorphism implicated in signaling pathways and the discordance of cancer predisposition among individuals.

In the process of metabolism, many factors such as exposure to environmental carcinogens and toxic metabolites may lead to the occurrence of DNA damage (3, 4). Under normal circumstances, our body deals with DNA damage through a complex set of repair mechanisms so that the hereditary material is balanced and stable to keep the body healthy. Nucleotide excision repair (*NER*) is one of the well-studied DNA repair pathways in human body, which reverses the multiform damage of the double-helix DNA with four steps: the recognition of lesion, the demarcation and unwinding of the impaired DNA fragment, oligonucleotide excision, and the ligation of new strands (5–7). The mutations of *NER* genes alter the capacity of DNA damage repairment, further resulting in an individual discrepancy of the risk of malignancy in tissue cells. Previous studies have identified that ineffective *NER* may give rise to incidence of a rare disease called xeroderma pigmentosum (*XP*), which can significantly increase the risk of skin cancer (3, 8).

As known to date, the functional performance of the *NER* pathway involved the participation of at least eight pivotal genes (*XP A-G* and *ERCC1*). The *XPF* gene, also known as excision repair cross-complementation group 4 (*ERCC4*), is located on chromosome 16p13.2 and consists of 11 exons that span approximately 28.2 *kb* (9). The proteins encoded by the *ERCC4* gene and *ERCC1* gene play a synergistic role in the *NER* pathway when participating in the excision of the damaged fragment (10, 11). Located on chromosome 13q22-33, consisting of 15 exons and 14 introns, the *XPG* gene is also termed as *ERCC5*, and the special endonuclease is produced by which it is indispensably enrolled in the two incision steps of the *NER* process (12). A growing number of genetic evidence indicated that the single-nucleotide polymorphisms (SNPs) in the *ERCC4* and *ERCC5* genes may vary susceptibility to malignant tumor; previous studies have demonstrated that *ERCC4* rs1800067 was associated with the risks of lung cancer, breast cancer, and glioma (13–15). Interestingly, the SNP rs17655 could trigger the occurrence of bladder cancer, leukemia, and glioma (16–18). Moreover, this SNP could downregulate the risk of head and neck cancer (19, 20) Variants other than the above-mentioned two SNPs in *ERCC4* and *ERCC5* have also been tested for the underlying relationship with the susceptibility to cancers, with inconsistent conclusions appearing due to the limitations of the sample and population.

Meta-analyses aiming to explore the relationship between *ERCC4* and *ERCC5* variants and the kinds of human cancers were continuously published (21, 22). However, most of these studies involved a single SNP and/or a single cancer; the conclusions are not always consistent, and the functional mechanisms remain unclear. Although in previous published meta-analysis studies, a single SNP with the risk of individual cancer was investigated, the results were still inconsistent. Furthermore, a comprehensive research synopsis with systematic functional annotation has not been performed to evaluate the epidemiological evidence of genetic associations between *ERCC4* or *ERCC5* genes and the risk of cancers till now. The purpose of the current study was to elucidate the role of all studied SNPs in *ERCC4* and *ERCC5* in the tendency of all implicated types of cancer. We firstly did meta-analysis with data collected from all relevant studies so far; then, the statistical power of generated significant evidence was detected. Finally, a systematic functional annotation was conducted for seeking the molecular mechanisms of approved connection.

## Methods and materials

We did this work with strict adherence to the guidelines of the Human Genome Epidemiology Network for systematic review of genetic association studies and Meta-analyses of Observational Studies in Epidemiology (MOOSE) and the Preferred Reporting Items for Systematic Reviews and Meta-Analyses Statement (PRISMA) guidelines **(**see **Supplementary Table S1** **)** (23–26)

### Literature searching and identification

A systematical article-searching was performed in the three most popular electronic databases: PubMed, Medline, and Web of Science. Eligible published studies up to 30 August 2021 were collected by using the following terms: “excision repair cross complementing group or *ERCC* or xeroderma pigmentosum group or *XP*” and “cancer or carcinoma or malignant tumor or adenocarcinoma” and “mutation or variant or variation or polymorphism or SNP or genotype.” Aside from articles originated from database, studies identified from meta-analyses and references were also added to the list.

### Criteria for inclusion and exclusion

We included genetic studies that meet the criteria below: (1) aiming to test the relationships between the *ERCC4* and/or *ERCC5* gene and the risk of cancer in case–control, cross-section, or cohort studies, (2) original articles published in a journal in English, (3) the concrete sample size of case and control groups and the quantity of genotype and/or allelic distributions were provided. Ineligible studies were excluded for these reasons: (1) studies researched the association between polymorphisms of other subgroup genes of *ERCC* and cancer risk; (2) meta-analyses, systematic reviews, pooled analyses, and duplicated publications; (3) adequate data (e.g., the amount of genotype) could not be acquired; and (4) studies focused only on the prognosis and survival of cancer patients, not cancer incidence.

### Data extraction and management

The authentic and precise data were independently extracted by two participators from qualified studies; all the divergences that occurred through the process were resolved by discussing with the corresponding author. Detailed information presented in the form including the first author, the year of publishing, cancer site, cancer type, country/region, ethnicity, genotyping methods, gene name, allele genotype and genotype distribution for each polymorphism, and minor allelic frequency (MAF). Ethnicity was comprised of four categories [Asian (East Asian descent), Caucasian (European descent), African (African descent), or others (including people from other countries such as Indians, Native Hawaiians, Latinos, Hispanics, and the mixed)] based on the criterion that at least 80% of the study populations belonged to one of these groups; “overall populations” integrates two or more. If the same genetic variant was reported in more than one study, we selected the most recently published study with the greatest number and most integrated participants. The specific minor allelic of each SNP were obtained from the website (https://www.ncbi.nlm.nih.gov/snp/).

### Statistical analysis

Meta-analyses were executed on the variants of more than one dataset, in which we employed three models: allelic, dominant, and recessive models for calculating the pooled ORs **(** **Supplementary Table S2** **)**. We also carried out a subgroup analysis of ethnicity among the SNPs with sufficient data. The heterogeneity across involved studies was examined by the utilization of Cochran’s *Q* statistic and the *I* ^2^ test (27, 28). Briefly, the *I* ^2^ value was categorized into *I* ^2^ ≤ 25%, 25% < *I* ^2^ < 50%, and *I* ^2^ ≥ 50%, which represented no or little heterogeneity, moderate heterogeneity, and large heterogeneity, respectively. Different kinds of models were employed according to the *P*-value generated from the *Q* statistic; the random effect model was used when the *P*-value <0.1, and the fixed effect model was appropriate for other circumstances. Furthermore, sensitivity analysis was applied to test the stability of significant ORs, which means producing a new OR value by excluding a single study (dataset), and/or the first published study, and/or studies that disobeyed the Hardy–Weinberg equilibrium (HWE) in the controls; it is an unstable association if the statistical significance was lost. We checked bias in two aspects: Begg’s test for potential publication bias and Egger’s test for small-study bias (29, 30). In this study, the strategy of affirming findings to be statistically significant was *P*-value <0.05 in the meta-analysis and *P-*value <0.10 in tests of heterogeneity and biases. An association was considered to be non-statistically significant if the 95% CI included 1.0 or if the P-value was ≥0.05. Statistical analyses were conducted utilizing Stata, version 12 (Stata, College Station, TX, USA).

### Assessment of cumulative evidence

The epidemiological credibility of statistically significant findings was primarily evaluated with Venice criteria (**Supplementary Notes for Venice criteria** **)** (23). Combined with the ratings of the three criteria (amount of evidence, replication, and protection from bias) and then got the assignment of grades as A, B, or C separately, the epidemiological evidence was ranked as strong, moderate, or weak. The amount of evidence was graded based on the result of sum of the tested alleles or genotype numbers in cases and controls, the sum more than 1,000, between 100 and 1,000, or less than 100 was graded as A, B, or C, respectively. To grade the replication, the consequences of heterogeneity estimation were employed as follows: A signified *I* ^2^ ≤ 25%, B signified 25% < *I* ^2^ < 50%, and C signified *I* ^2^ ≥ 50%. The grade of protection from bias was generated from comprehensively analyzing the outcome of sensitivity analysis, statistic of publication bias and small study bias, and assessment of an excess of significant findings. Eventually, grade A was assigned if no apparent bias was observed, or bias was unable for illuminating the presence of association, grade B would be assigned if we got moderate bias, and grade C was assigned if there was evident bias or bias could explain the existence of association. Meanwhile, connection intention was a non-negligible factor of the evaluation of the protection from bias; grade C was assigned on this criterion when the pooled OR was less than 1.15 (or more than 0.87 in a protection effect). However, this rule would be invalid if this significant finding had been replicated extensively by large collaborative studies including GWAS or GWAS meta-analysis (31). We strictly adhere to the checklist when checking the sources of bias in different settings proposed by the Venice criteria (see **Supplementary Information Notes**). Subsequently, those significant findings with grade A for all three criteria were determined as strong-credibility epidemiological evidence, those with grades were composed of A and B were determined as moderate-credibility evidence, and those with C assigned to any of three categories were considered as weak-credibility evidence.

A prior probability of 0.05 and a false-positive report probability (FPRP) cut-off value of 0.2 in the FPRP assay should be performed to detect the potential false-positive results among significant associations and assess whether these associations should be excluded, as Wacholder et al. recommended (32). If the calculated FPRP value was below the prespecified noteworthiness value of 0.2, we would consider the association noteworthy, indicating that the association might be true. The true evidence was graded by the FPRP value: <0.05, 0.05–0.2, or >0.2, indicating strong, moderate, or weak, respectively. With a strong magnitude of FPRP, the credibility of evidence would be upgraded from weak to moderate and from moderate to strong; if FPRP was assigned as weak, we would downgrade the credibility of association from strong to moderate and from moderate to weak. We utilized an Excel spreadsheet established by Wacholder et al. for calculating the FPRP values and corresponding statistical power.

### Functional annotation

The underlying functional role of the variants of *ERCC4* and *ERCC5* genes was evaluated with information from the Encyclopedia of DNA Elements (ENCODE) tool HaploReg (v4.1) (32) as well as UCSC Genome browser (http://genome.ucsc.edu/). Furthermore, the current work explored genome-wide cis-eQTL data in multiple tissues from the Genotype-Tissue Expression Project (33) and the Multiple Tissue Human Expression Resource Project (33) databases in order to reveal whether these genes might explain the observed findings in these loci.

## Results

### Characteristics of included studies

**Figure 1:**
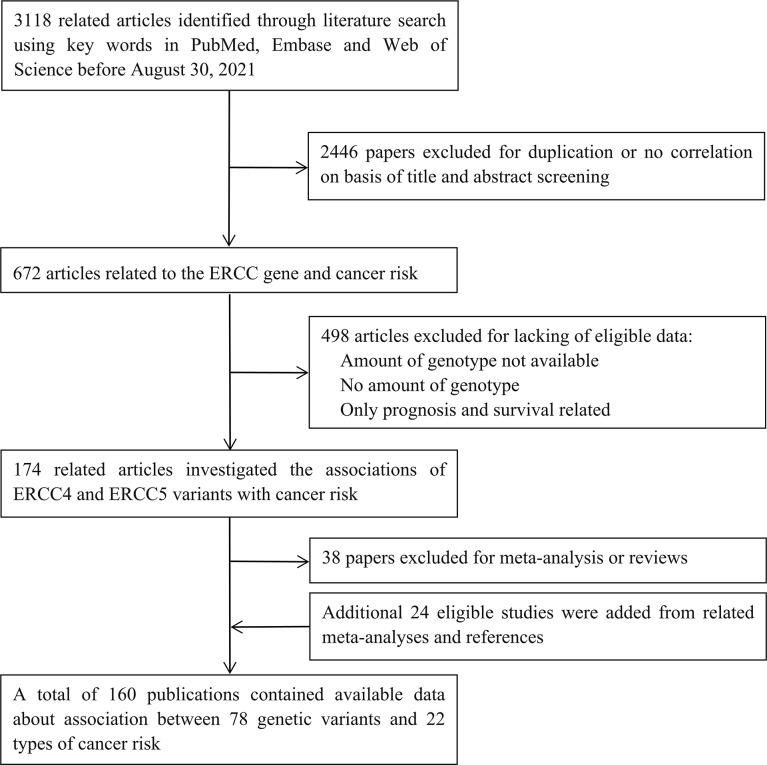
Flow diagram of literature search and study selection.

Initially, 3,118 studies were retrieved from PubMed, Medline, and Web of Science (**Figure 1**). After reviewing the title and abstract, we filtered out 672 articles related to the *ERCC* gene and cancer risk; those were excluded because of duplication or no correlation. Then, 498 articles were excluded due to the lack of eligible data: not SNPs of *ERCC4* and *ERCC5*; no amount of genotype; and prognosis and survival related. Next, 38 papers of meta-analysis and review were excluded; additional 24 studies were added from related meta-analyses and references. Ultimately, 160 publications were eligible, including 192 datasets in 84 publications of *ERCC4* and 280 datasets in 123 articles of *ERCC5* (47 articles containing data about both *ERCC4* and *ERCC5*). The demographical characteristics of all available publications are summarized in **Supplementary Table S3**. In current study, 55,446 cases of 19 types of cancer and 61,855 controls were enrolled in these 192 datasets for the investigation of the implication of 40 *ERCC4* variants on cancer susceptibility, and as for *ERCC5*, we collected 38 SNPs distributed in 55,393 cases of 22 types of cancer and 66,872 controls. A total of 19 types of cancer and 24 SNPs of both *ERCC4* and *ERCC5* were incorporated into meta-analysis because there were at least two serviceable datasets.

### Main meta-analyses

#### ERCC4

We executed meta-analysis on the correlation between 12 variants of *ERCC4* and the risk of 13 types of cancer, and four SNPs (rs744154, rs1800067, rs2276466, and rs1799801) were figured out to be significantly associated with risk of three cancers (bladder cancer, gastric cancer, and glioma) **(** **Table 1** **)**. To be specific, rs744154 (C vs. G) was confirmed to be a risk factor of bladder cancer in Asians in the allelic model (OR = 1.566, 95% CI = 1.233-1.989, *p* < 0.001) and recessive model (OR = 1.731, 95% CI = 1.296-2.313, *p* < 0.001). Two SNPs were significantly associated with the risk of glioma, an increased susceptibility was observed for rs2276466 (G vs. C) in Asians (allelic model: OR = 1.332, 95% CI = 1.101-1.612, *p* = 0.003; dominant model: OR = 1.336, 95% CI = 1.030-1.733, *p* = 0.029; recessive model: OR = 1.553, 95% CI = 1.094-2.206, *p* = 0.014); nevertheless, we demonstrated that rs1800067 (A vs. G) was a protective factor in the occurrence of glioma in the overall population (allelic model: OR = 0.634, 95% CI = 0.426-0.944, *p* = 0.025; recessive model: OR = 0.528, 95% CI = 0.350-0.796, *p* = 0.002). In addition, significant associations with the risk of gastric cancer were revealed for rs1799801 (C vs. T) in the overall population in the dominant model (OR = 0.755, 95% CI = 0.614-0.930, *p* = 0.008), and for rs744154 (C vs. G) in Asians (allelic model: OR = 0.790, 95% CI = 0.666-0.937, *p* < 0.007; dominant model: OR = 0.681, 95% CI = 0.535-0.866, *p* = 0.020).

**Table 1:** Genetic variants showing significant summary odds ratios for different cancer risks in main meta-analyses in all three genetic models.

Gene	Variant	Alleles^a^	Cancer site	Ethnicity	MAF^b^	Genetic models	Effect model	Number Evaluation		Risk of Meta-Analysis		Heterogeneity		Venice criteria Grade^c^	FPRP values^d^	Credibility of evidence^e^
								Study	Sample size (case/controls)	OR (95% CI)	P value	I^2^ (%)	P_Q_			
ERCC4	rs744154	C vs. G	Bladder	Asian	0.7213	Allelic	Fixed	2	484 (234/250)	1.566 (1.233-1.989)	0.000	0.0	0.789	AAA	0.012	Strong
ERCC4	rs744154	C vs. G	Bladder	Asian	0.7213	Recessive	Fixed	2	484 (234/250)	1.731 (1.296-2.313)	0.000	0.0	0.862	BAA	0.023	Strong
ERCC4	rs1800067	A vs. G	Brain (Glioma)	Overall	0.7253	Allelic	Random	5	3502 (1481/2021)	0.634 (0.426-0.944)	0.025	82.9	0.000	ACC	0.540	Weak
ERCC4	rs1800067	A vs. G	Brain (Glioma)	Overall	0.7253	Recessive	Random	5	3502 (1481/2021)	0.528 (0.350-0.796)	0.002	69.3	0.011	ACA	0.247	Weak
ERCC4	rs1800067	A vs. G	Brain (Glioma)	Asian	0.9263	Allelic	Random	4	2645 (1119/1526)	0.543 (0.389-0.760)	0.000	69.5	0.020	ACA	0.057	Weak
ERCC4	rs1800067	A vs. G	Brain (Glioma)	Asian	0.9263	Dominant	Random	4	2645 (1119/1526)	0.502 (0.312-0.808)	0.005	0.0	0.825	AAC	0.416	Weak
ERCC4	rs1800067	A vs. G	Brain (Glioma)	Asian	0.9263	Recessive	Random	4	2645 (1119/1526)	0.535 (0.347-0.826)	0.005	76.7	0.005	ACC	0.361	Weak
ERCC4	rs2276466	G vs. C	Brain (Glioma)	Asian	0.3243	Allelic	Fixed	2	930 (432/498)	1.332 (1.101-1.612)	0.003	0.0	0.878	BAA	0.065	Moderate
ERCC4	rs2276466	G vs. C	Brain (Glioma)	Asian	0.3243	Dominant	Fixed	2	930 (432/498)	1.336 (1.030-1.733)	0.029	0.0	0.881	BAA	0.4240	Weak
ERCC4	rs2276466	G vs. C	Brain (Glioma)	Asian	0.3243	Recessive	Fixed	2	930 (432/498)	1.553 (1.094-2.206)	0.014	0.0	0.917	BAA	0.385	Weak
ERCC4	rs1799801	C vs. T	Stomach	Overall	0.4027	Dominant	Fixed	3	2796 (1327/1469)	0.755 (0.614-0.930)	0.008	42.2	0.177	ABA	0.151	Moderate
ERCC4	rs744154	C vs. G	Stomach	Asian	0.4854	Allelic	Fixed	2	1504 (681/823)	0.790 (0.666-0.937)	0.007	62.2	0.104	ACA	0.117	Weak
ERCC4	rs744154	C vs. G	Stomach	Asian	0.4854	Dominant	Fixed	2	1504 (681/823)	0.681 (0.535-0.866)	0.020	37.6	0.2060	BBA	0.055	Moderate
ERCC5	rs17655	C vs. G	Blood	Overall	0.2597	Allelic	Random	7	5100 (2592/2508)	1.176 (1.017-1.360)	0.029	50.2	0.061	ACA	0.354	Weak
ERCC5	rs17655	C vs. G	Blood	Overall	0.2597	Dominant	Fixed	7	5100 (2592/2508)	1.169 (1.041-1.313)	0.009	37.3	0.144	ABA	0.138	Moderate
ERCC5	rs17655	C vs. G	Blood	Caucasian	0.2517	Allelic	Random	4	2114 (1014/1100)	1.298 (1.007-1.671)	0.044	59.0	0.062	ACA	0.484	Weak
ERCC5	rs17655	C vs. G	Blood	Caucasian	0.2517	Dominant	Fixed	4	2114 (1014/1100)	1.285 (1.065-1.550)	0.009	61.0	0.051	BCA	0.149	Weak
ERCC5	rs17655	C vs. G	Blood	Caucasian	0.2517	Recessive	Fixed	4	2114 (1014/1100)	1.511 (1.093-2.090)	0.013	0.0	0.472	BAA	0.332	Weak
ERCC5	rs1047768	C vs. T	Colorectum	Asian	0.2815	Recessive	Random	2	55889 (2755/2834)	1.218 (1.006-1.474)	0.044	0.0	0.367	BAA	0.452	Weak
ERCC5	rs1047768	C vs. T	Colorectum	Caucasian	0.5512	Allelic	Fixed	2	3305 (1135/2170)	0.876 (0.774-0.991)	0.036	44.5	0.180	BBC	0.402	Weak
ERCC5	rs17655	C vs. G	Colorectum	Overall	0.3667	Allelic	Fixed	13	17943 (8068/9875)	1.053 (1.006-1.102)	0.027	18.3	0.259	AAC	0.331	Weak
ERCC5	rs17655	C vs. G	Colorectum	Overall	0.3667	Dominant	Random	13	17943 (8068/9875)	1.132 (1.020-1.255)	0.019	53.3	0.012	ACC	0.260	Weak
ERCC5	rs17655	C vs. G	Colorectum	Caucasian	0.2283	Dominant	Random	7	7206 (2858/4348)	1.118 (1.006-1.242)	0.038	0.0	0.431	AAC	0.417	Weak
ERCC5	rs2094258	T vs. C	Colorectum	Asian	0.3394	Allelic	Fixed	2	5589 (2755/2834)	1.128 (1.043-1.219)	0.002	9.0	0.295	AAC	0.043	Moderate
ERCC5	rs2094258	T vs. C	Colorectum	Asian	0.3394	Dominant	Fixed	2	5589 (2755/2834)	1.141 (1.025-1.270)	0.015	0.0	0.706	AAC	0.231	Weak
ERCC5	rs2094258	T vs. C	Colorectum	Asian	0.3394	Recessive	Fixed	2	5589 (2755/2834)	1.232 (1.051-1.445)	0.010	59.8	0.115	BCA	0.165	Weak
ERCC5	rs2296147	T vs. C	Esophagus	Asian	0.2550	Allelic	Fixed	2	4292 (1672/2620)	0.825 (0.741-0.919)	0.000	0.0	0.531	AAA	0.009	Strong
ERCC5	rs2296147	T vs. C	Esophagus	Asian	0.2550	Dominant	Fixed	2	4292 (1672/2620)	0.783 (0.687-0.892)	0.000	0.0	0.868	AAA	0.004	Strong
ERCC5	rs17655	C vs. G	Head and Neck	Overall	0.4481	Recessive	Random	6	6772 (2919/3853)	0.787 (0.627-0.989)	0.040	58.8	0.033	ACC	0.451	Weak
ERCC5	rs17655	C vs. G	Head and Neck	Asian	0.5252	Dominant	Fixed	3	2069 (783/1286)	0.796 (0.649-0.976)	0.028	54.0	0.114	ACC	0.360	Weak
ERCC5	rs17655	C vs. G	Laryngeal	Overall	0.3732	Recessive	Fixed	3	1667 (634/1033)	0.571 (0.533-0.753)	0.000	0.0	0.933	BAC	0.010	Moderate
ERCC5	rs17655	C vs. G	Laryngeal	Asian	0.6049	Allelic	Random	2	772 (386/386)	0.636 (0.520-0.779)	0.000	0.0	0.683	BAA	0.001	Strong
ERCC5	rs17655	C vs. G	Laryngeal	Asian	0.6049	Dominant	Random	2	772 (386/386)	0.613 (0.444-0.847)	0.003	0.0	0.770	BAA	0.158	Moderate
ERCC5	rs17655	C vs. G	Laryngeal	Asian	0.6049	Recessive	Fixed	2	772 (386/386)	0.574 (0.427-0.772)	0.000	0.0	0.720	BAA	0.025	Strong
ERCC5	rs2228959	A vs. C	Lung	Asian	0.2186	Allelic	Fixed	2	984 (492/492)	0.370 (0.283-0.484)	0.000	61.6	0.107	BCA	0.000	Moderate
ERCC5	rs2228959	A vs. C	Lung	Asian	0.2186	Dominant	Random	2	984 (492/492)	0.429 (0.188-0.979)	0.044	66.9	0.082	BCA	0.852	Weak
ERCC5	rs17655	C vs. G	Oral	Asian	0.4433	Allelic	Fixed	2	965 (424/541)	1.334 (1.105-1.611)	0.003	0.0	0.365	BAA	0.054	Moderate
ERCC5	rs17655	C vs. G	Oral	Asian	0.4433	Dominant	Fixed	2	965 (424/541)	1.414 (1.059-1.889)	0.019	0.0	0.974	BAA	0.359	Weak
ERCC5	rs17655	C vs. G	Oral	Asian	0.4433	Recessive	Fixed	2	965 (424/541)	1.485 (1.082-2.038)	0.014	52.3	0.148	BCA	0.347	Weak
ERCC5	rs17655	C vs. G	Prostate	Overall	0.3844	Dominant	Fixed	4	4755 (2128/2627)	1.149 (1.005-1.312)	0.042	24.8	0.263	AAC	0.419	Weak
ERCC5	rs17655	C vs. G	Stomach	Caucasian	0.5808	Allelic	Fixed	2	1493 (643/850)	1.282 (1.024-1.606)	0.030	18.3	0.269	AAA	0.404	Moderate
ERCC5	rs17655	C vs. G	Stomach	Caucasian	0.5808	Recessive	Random	2	1493 (643/850)	1.513 (1.126-2.034)	0.006	0.0	0.686	BAA	0.187	Moderate
ERCC5	rs751402	G vs. A	Stomach	Asian	0.6558	Allelic	Random	10	8565 (3989/4576)	0.865 (0.784-0.954)	0.004	51.8	0.028	ACA	0.044	Moderate
ERCC5	rs751402	G vs. A	Stomach	Asian	0.6558	Dominant	Random	10	8565 (3989/4576)	0.802 (0.657-0.980)	0.031	50.4	0.034	ACC	0.379	Weak
ERCC5	rs751402	G vs. A	Stomach	Asian	0.6558	Recessive	Fixed	10	8565 (3989/4576)	0.867 (0.794-0.946)	0.001	6.7	0.380	AAA	0.010	Strong
ERCC5	rs873601	A vs. G	Stomach	Asian	0.4757	Allelic	Fixed	6	8717 (4177/4540)	1.069 (1.007-1.135)	0.029	29.2	0.216	ABC	0.444	Weak
ERCC5	rs873601	A vs. G	Stomach	Asian	0.4757	Recessive	Fixed	6	8717 (4177/4540)	1.133 (1.026-1.251)	0.014	22.5	0.265	AAC	0.195	Weak
ERCC5	rs17655	C vs. G	Thyroid	Caucasian	0.5551	Recessive	Fixed	2	647 (181/466)	0.501 (0.313-0.801)	0.004	0.0	0.491	BAA	0.388	Weak
ERCC5	rs17655	C vs. G	Uterus	Overall	0.3544	Allelic	Random	5	4431 (1936/2495)	1.239 (1.050-1.463)	0.011	63.5	0.027	ACA	0.181	Weak
ERCC5	rs17655	C vs. G	Uterus	Overall	0.3544	Dominant	Random	5	4431 (1936/2495)	1.315 (1.039-1.664)	0.023	63.5	0.027	ACC	0.332	Weak
ERCC5	rs17655	C vs. G	Uterus	Overall	0.3544	Recessive	Fixed	5	4431 (1936/2495)	1.355 (1.142-1.608)	0.001	0.0	0.482	AAA	0.011	Strong
ERCC5	rs17655	C vs. G	Cervix	Asian	0.4764	Allelic	Random	2	1800 (678/1122)	1.365 (1.190-1.565)	0.000	0.0	0.483	AAA	0.000	Strong
ERCC5	rs17655	C vs. G	Cervix	Asian	0.4764	Dominant	Random	2	1800 (678/1122)	1.618 (1.286-2.035)	0.000	0.0	0.820	AAA	0.003	Strong
ERCC5	rs17655	C vs. G	Cervix	Asian	0.4764	Recessive	Fixed	2	1800 (678/1122)	1.387 (1.121-1.715)	0.003	43.6	0.183	BBA	0.059	Moderate

OR, odds ratio; A, adenine; T, thymine; G, guanine; C, cytosine; ERCC: excision repair cross-complementation.

a Minor alleles vs. major alleles (reference).

b Frequency of minor allele in controls.

c Strength of epidemiological evidence based on the Venice criteria.

d FPRP values at prior probability of 0.05 at power OR of 1.5, and the FPRP level of noteworthiness is 0.20.

e Degree of epidemiological credibility based on the combination of results from Venice guidelines and FPRP tests.

The results of subgroup analysis by ethnicity showed that rs1800067 (A vs. G) could decrease the risk of glioma in Asians (allelic model: OR = 0.543, 95% CI = 0.389-0.760, *p* < 0.001; dominant model: OR = 0.502, 95% CI = 0.312-0.808, *p* = 0.005; recessive model: OR = 0.535, 95% CI = 0.347-0.826, *p* = 0.005) but did not in Caucasians. In addition, there was no relationship between rs1799801 (C vs. T) and gastric cancer risk in Asians but in Caucasians (allelic model: OR = 0.698, 95% CI = 0.505-0.963, *p* < 0.029; dominant model: OR = 0.567, 95% CI = 0.378-0.849, *p* = 0.006).

#### ERCC5

A total of 12 SNPs of the *ERCC5* gene and 15 types of cancer were involved into meta-analyses **(** **Table 1** **)**. Rs17655 (C vs. G); the most extensively researched variants were testified to be significantly associated with the risk of nine cancers in the overall population, including leukemia (allelic model: OR = 1.176, 95% CI = 1.017-1.360, *p* = 0.029; dominant model: OR = 1.169, 95% CI = 1.041-1.313, *p* = 0.009), colorectal cancer (allelic model: OR = 1.053, 95% CI = 1.006-1.102, *p* = 0.027; dominant model: OR = 1.132, 95% CI = 1.020-1.255, *p* = 0.019), head and neck cancer (recessive model: OR = 0.787, 95% CI = 0.627-0.989, *p* = 0.040), laryngeal cancer (recessive model: OR = 0.571, 95% CI = 0.533-0.753, *p* < 0.001), prostate cancer (dominant model: OR = 1.149, 95% CI = 1.005-1.312, *p* = 0.042), and uterine cancer (allelic model: OR = 1.239, 95% CI = 1.050-1.463, *p* = 0.011; dominant model: OR = 1.315, 95% CI = 1.039-1.664, *p* = 0.023; recessive model: OR = 1.355, 95% CI = 1.142-1.608, *p* = 0.001).

Through the subgroup analysis by ethnicity, we got these following associations between rs17655 (C vs. G) and cancers: increased risk of leukemia in Caucasians (allelic model: OR = 1.298, 95% CI = 1.007-1.671, *p* = 0.044; dominant model: OR = 1.285, 95% CI = 1.065-1.550, *p* = 0.009; recessive model: OR = 1.511, 95% CI = 1.093-2.090, *p* = 0.013), colorectal cancer in Caucasians (dominant model: OR = 1.118, 95% CI = 1.006-1.242, *p* = 0.038), oral cancer in Asians (allelic model: OR = 1.334, 95% CI = 1.105-1.611, *p* = 0.003; dominant model: OR = 1.414, 95% CI = 1.059-1.889, *p* = 0.019; recessive model: OR = 1.485, 95% CI = 1.082-2.038, *p* = 0.014), prostate cancer in Caucasians (allelic model: OR = 1.208, 95% CI = 1.003-1.454, *p* =< 0.046), gastric cancer in Caucasians (allelic model: OR = 1.282, 95% CI = 1.024-1.606, *p* = 0.030; recessive model: OR = 1.513, 95% CI = 1.126-2.034, *p* = 0.006), uterine cancer in Asians (allelic model: OR = 01.365, 95% CI = 1.190-1.565, *p* < 0.001; dominant model: OR = 1.618, 95% CI = 1.286-2.035, *p* < 0.001; recessive model: OR = 1.387, 95% CI = 1.121-1.715, *p* = 0.003); a decreased risk of head and neck cancer in Asians (dominant model: OR = 0.796, 95% CI = 0.649-0.976, *p* = 0.028), laryngeal cancer in Asians (allelic model: OR = 0.636, 95% CI = 0.520-0.779, *p* < 0.001; dominant model: OR = 0.613, 95% CI = 0.444-0.847, *p* = 0.003; recessive model: OR = 0.574, 95% CI = 0.427-0.772, *p* < 0.001), and thyroid cancer in Caucasians in the recessive model (OR = 0.501, 95% CI = 0.313-0.801, *p* = 0.004).

With the exception of rs17655 (C vs. G), six SNPs (rs1047768, rs2094258, rs2296147, rs2228959, rs751402, and rs873601) of *ERCC5* were also demonstrated to significantly alter the susceptibility of cancers. We found that rs1047768 (C vs. T) remarkably increased the risk of colorectal cancer in Asians in the recessive model (OR = 1.218, 95% CI = 1.006-1.474, *p* = 0.044), in contrast, it is a protective factor of colorectal cancer in Caucasians in the allelic model (OR = 0.876, 95% CI = 0.774-0.991, *p* = 0.036). Another significant association with the risk of colorectal cancer was observed for rs2094258 (T vs. C) in Asians (allelic model: OR = 1.128, 95% CI = 1.043-1.219, *p* = 0.002; dominant model: OR = 1.141, 95% CI = 1.025-1.270, *p* = 0.015; recessive model: OR = 1.232, 95% CI = 1.051-1.445, *p* = 0.010). It was uncovered that rs2296147 (T vs. C) polymorphism was relevant to the decreased risk of esophageal cancer in Asians (allelic model: OR = 0.825, 95% CI = 0.741-0.919, *p* < 0.001; dominant model: OR = 0.783, 95% CI = 0.687-0.892, *p* < 0.001), and the same association was shown between rs2228959 (A vs. C) and lung cancer in Asians (allelic model: OR = 0.370, 95% CI = 0.283-0.484, *p* < 0.001; dominant model: OR = 0.429, 95% CI = 0.188-0.979, *p* = 0.044). When researching the incidence of gastric cancer in Asians, a protective effect was observed in the implication of rs751402 (G vs. A) polymorphism on gastric cancer (allelic model: OR = 0.865, 95% CI = 0.784-0.954, *p* = 0.004; dominant model: OR = 0.802, 95% CI = 0.657-0.980, *p* = 0.031; recessive model: OR = 0.867, 95% CI = 0.794-0.946, *p* = 0.001); however, the opposite effect appeared when it comes to rs873601 (A vs. G) in the allelic model (OR = 1.069, 95% CI = 1.007-1.135, *p* = 0.029) and recessive model (OR = 1.133, 95% CI = 1.026-1.251, *p* = 0.014).

### Non-significant association in meta‐analyses

We additionally found that among those associations lack of statistical significance, five polymorphisms (two of *ERCC4* and three of *ERCC5*) had no evidence of relationship with four cancers risk in meta-analyses with at least 3,000 cases and 3,000 controls **(** **Table 2** **)**.

**Table 2:** Variants showing no relation to cancer risk in meta-analyses with at least 3,000 cases and 3,000 controls in additive model.

Gene	Variant	Alleles^a^	Cancer site	Ethnicity	MAF^b^	Effect model	Number Evaluation		Risk of Meta-Analysis		Heterogeneity		Power(%)	The value of Power (%) if the MAF is 0.2	The value of power (%) if the MAF is 0.1
							Studies	Sample size (case/controls)	OR (95% CI)	P value	I^2^ (%)	P_Q_			
ERCC4	rs1800067	A vs. G	Breast	Overall	0.1040	Random	12	17,885 (9,310/8,575)	1.011 (0902-1.133)	0.850	37.5	0.091	98.2	100.0	97.9
ERCC4	rs1800067	A vs. G	Breast	Caucasian	0.0696	Random	9	15,228 (7,936/7,292)	1.042 (0.924-1.176)	0.502	38.6	0.111	87.9	99.8	95.7
ERCC4	rs744154	C vs. G	Breast	Overall	0.3517	Fixed	4	81,066 (41,439/39,627)	1.000 (0.978-1.023)	0.997	16.5	0.309	100.0	100.0	100.0
ERCC4	rs744154	C vs. G	Breast	Caucasian	0.3598	Fixed	2	75,040 (3,8088/36,952)	0.996 (0.972-1.019)	0.714	40.3	0.195	100.0	100.0	100.0
ERCC5	rs17655	C vs. G	Breast	Overall	0.3003	Random	15	17,222 (8,341/8,881)	1.039 (0.959-1.125)	0.355	51.6	0.011	100.0	99.9	97.6
ERCC5	rs17655	C vs. G	Breast	Caucasian	0.2392	Random	8	12,053 (5,873/6,180)	1.071 (0.962-1.195)	0.109	58.8	0.017	99.6	99.2	91.0
ERCC5	rs17655	C vs. G	Lung	Overall	0.4252	Random	11	9,728 (4,284/5,444)	1.078 (0.961-1.209)	0.199	65.2	0.001	99.5	96.2	80.5
ERCC5	rs17655	C vs. G	Skin	Caucasian	0.3245	Fixed	10	11,284 (5,162/6,122)	0.951 (0.890-1.015)	0.133	0.0	0.670	99.7	98.4	87.2
ERCC5	rs2094258	T vs. C	Stomach	Asian	0.3885	Random	9	9,884 (4,648/5,236)	1.022 (0.929-1.124)	0.652	54.9	0.023	99.7	97.3	83.6
ERCC5	rs2296147	C vs. T	Stomach	Asian	0.2121	Fixed	5	7,589 (3,699/3,890)	0.989 (0.915-1.069)	0.778	33.7	0.197	94.3	93.4	74.6

OR, odds ratio; A, adenine; C, cytosine; G, guanine; T, thymine; ERCC: excision repair cross-complementation.

a Minor alleles vs. major alleles (reference).

b Frequency of minor allele in controls.

### Heterogeneity, sensitivity analyses, and publication bias

Among all the significant findings of the correlation between variants of *ERCC4* and *ERCC5* and cancer risk, little heterogeneity (*I* ^2^ ≤ 25%) was discovered in 29 (53.7%) relationships; moderate (25% < *I* ^2^ < 50%) and large (*I* ^2^ ≥ 50%) heterogeneity were figured out in 6 (11.1%) and 19 (35.2%) associations.(**Table 1**) The results of sensitivity analysis are shown in **Table 1**. We identified that 12 associations were dusted on the account of the removal of a single study (dataset); the first published and/or studies deviated from HWE in controls, including rs1800067 with glioma in the overall population in the allelic model and in the Asians in the dominant model, rs17655 with colorectal cancer in overall population in the allelic model and in Caucasians in the dominant model, rs17655 with head and neck cancer in the overall population in recessive and in Asians in the dominant model, rs17655 with laryngeal cancer in the overall population in the recessive model, rs17655 with prostate cancer in the overall population in dominant model, rs751402 and gastric cancer in Asians in the dominant model, rs873601 and gastric cancer in the allelic and recessive model, and rs17655 with uterine cancer in the overall population in the dominant model. The evidence of significant publication bias (*p* < 0.1) was found in two connections (rs17655 with head and neck cancer in the overall population in recessive, rs1800067 with glioma in Asians in the recessive model). We could not test the excess of significant finding because of the absence of data of the genotype or allele in most of the studies **(** **Supplementary Table S4** **)**.

### Cumulative evidence of significant findings

We conducted epidemiological evidence evaluation on all of the 54 significant associations, 10 of which were rated as strong credibility, 13 results were rated as moderate credibility, and 31 associations were rated as weak credibility **(** **Table 1** **)**. Firstly, by assessing the amount of evidence of the Venice criteria, we got 32 relationships that were assigned grade A, and 22 others were assigned grade B. In terms of replication, grade A was distributed in 29 findings, grade B in 6 findings, and grade C in 19 results. As for protection from bias, grades A, B, and C were assigned to 37, 0, and 17 associations. In summary, 17, 8, and 29 evidence were separately determined as strong, moderate, and weak credibility in the Venice criteria (**Table 2**). Subsequently, the FPRP values of all the significant findings were computed for the evaluation of the probability of true association. With the result of the FPRP value < 0.05, the rate of credibility was upgraded from moderate to strong in three findings (rs744154 and bladder cancer in Asians in the recessive model, rs17655 and laryngeal cancer in Asians in allelic and recessive models), and from weak to moderate in four associations (rs2094258 and colorectal cancer in Asians in the allelic model, rs17655 and laryngeal cancer in the overall population in the recessive model, rs2228959 and lung cancer in Asians in the allelic model, rs751402 and gastric cancer in Asians in the allelic model). On the contrary, owing to FPRP values >0.2, the credibility of evidence in one connection (rs17655 and gastric cancer in Caucasians in the allelic model) were downgraded from strong to moderate, and six of the findings (rs2276466 and glioma in Asians in dominant and recessive models, rs17655 and leukemia in Caucasians in the recessive model, rs1047768 and colorectal cancer in Asians in the recessive model, rs17655 and oral cancer in Asians in the dominant model, rs17655 and thyroid cancer in Caucasians in the recessive model) were downgraded from moderate to weak. Ultimately, we got 10 strong-credibility evidence incorporating rs744154 and bladder cancer in Asians in allelic and recessive models, rs2296147 and esophageal cancer in Asians in allelic and dominant models, rs17655 and laryngeal cancer in Asians in allelic and recessive models, rs751402 and gastric cancer in Asians in the recessive model, and rs17655 and uterine cancer in overall population in the recessive model and in Asians in allelic and dominant models **(** **Supplementary Table S5** **)**.

### Functional annotation

**Figure 2:**
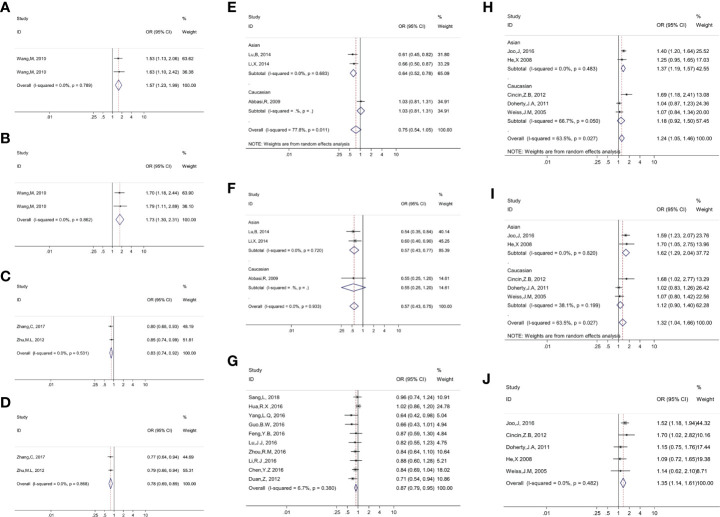
Presented forest plot of 10 strong-credibility evidence: **(A).** association between ERCC4 rs744154 and bladder cancer risk in the Asian population in the allelic model; **(B).** association between ERCC4 rs744154 and bladder cancer risk in the Asian population in the recessive model; **(C).** association between ERCC5 rs2296147 and esophageal cancer risk in the Asian population in the allelic model; **(D).** association between ERCC5 rs2296147 and esophageal cancer risk in the Asian population in the dominant model; **(E).** association between ERCC5 rs17655 and laryngeal cancer risk in the allelic model, stratified by ethnicity; **(F).** association between ERCC5 rs17655 and laryngeal cancer risk in the recessive model, stratified by ethnicity; **(G).** association between ERCC5 rs751402 and gastric cancer risk in Asian population in the recessive model; **(H).** association between ERCC5 rs17655 and Uterine cancer risk in the allelic model, stratified by ethnicity; **(I).** association between ERCC5 rs17655 and uterine cancer risk in the dominant model, stratified by ethnicity; **(J).** association between ERCC5 rs17655 and uterine cancer risk in overall population in the recessive model.

**Figure 3:**
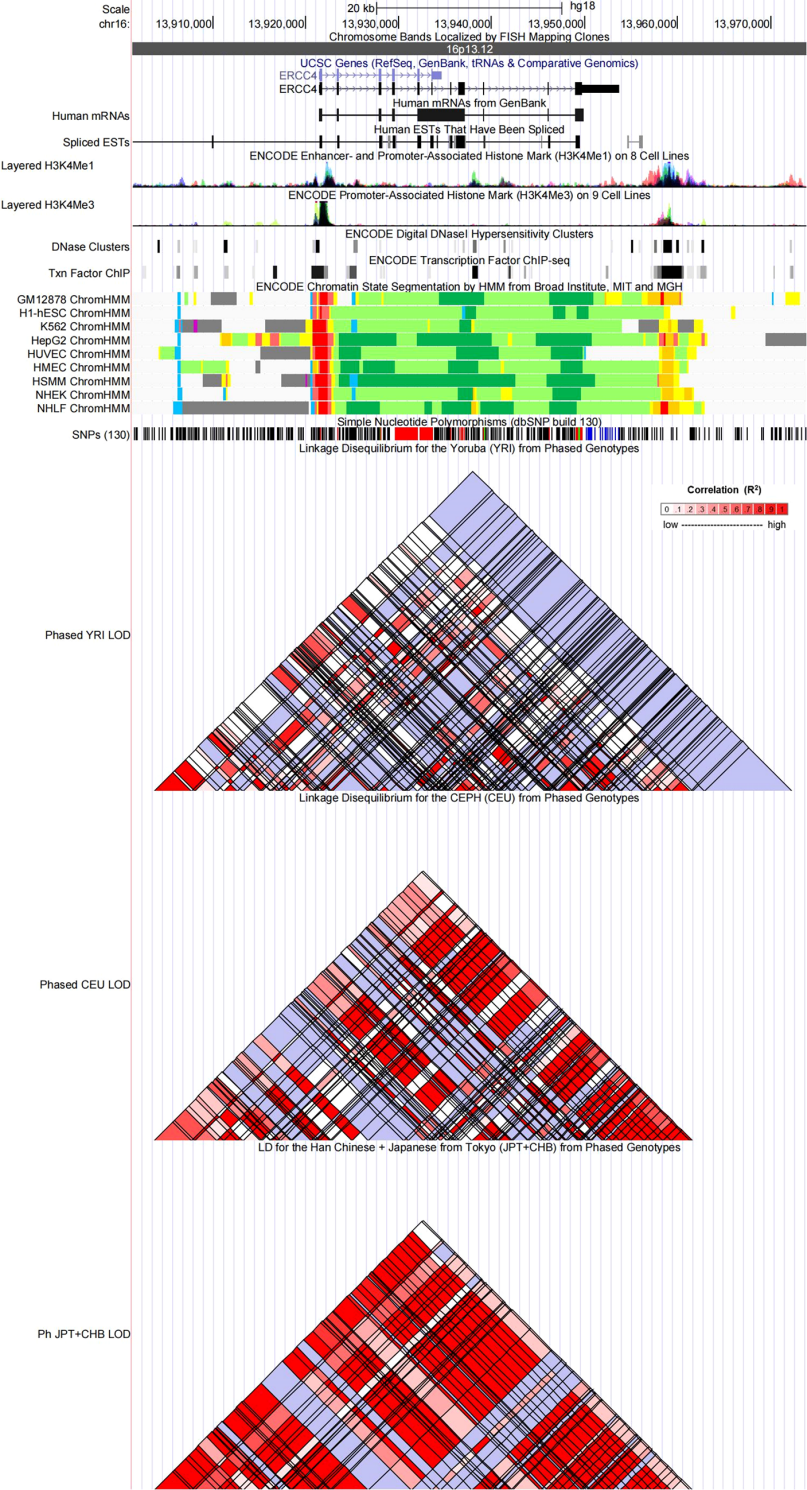
Evidence from the Encyclopedia of DNA Elements (ENCODE) data for the regulatory function of variants in 16p13.12 using the UCSC Genome Browser. The plot represents 16p13.12 within a 20-kb window centered on the ERCC4 gene region. Tracks (from top to bottom) in each of the plots are Genome Base Position, Chromosome Bands, UCSC Genes, Human messenger RNAs from GenBank, Human expressed sequence tag (ESTs) That Have Been Spliced, ENCODE Enhancer and Promoter-Associated Histone Mark (H3K4Me1) on 8 Cell Lines, ENCODE Promoter-Associated Histone Mark (H3K4Me3) on 9 Cell Lines, ENCODE Digital DNaseI Hypersensitivity Clusters, ENCODE Transcription Factor ChIP-seq, ENCODE Chromatin State Segmentation by Hidden Markov Model (HMM) from Broad Institute (bright red, active promoter; light red, weak promoter; purple, inactive/poised promoter; orange, strong enhancer; yellow, weak/poised enhancer; blue, insulator; dark green, transcriptional transition/elongation; light green, weak transcribed; gray, polycomb-repressed; light gray, heterochromatin/low signal/repetitive/copy number variation), Simple Nucleotide Polymorphisms (dbSNP build 130), Linkage Disequilibrium for the Yoruba (YRI) from Phased Genotypes, Linkage Disequilibrium for the CEPH (CEU) from Phased Genotypes and LD for the Han Chinese + Japanese from Tokyo (JPT+CHB) from Phased Genotypes.

**Figure 4:**
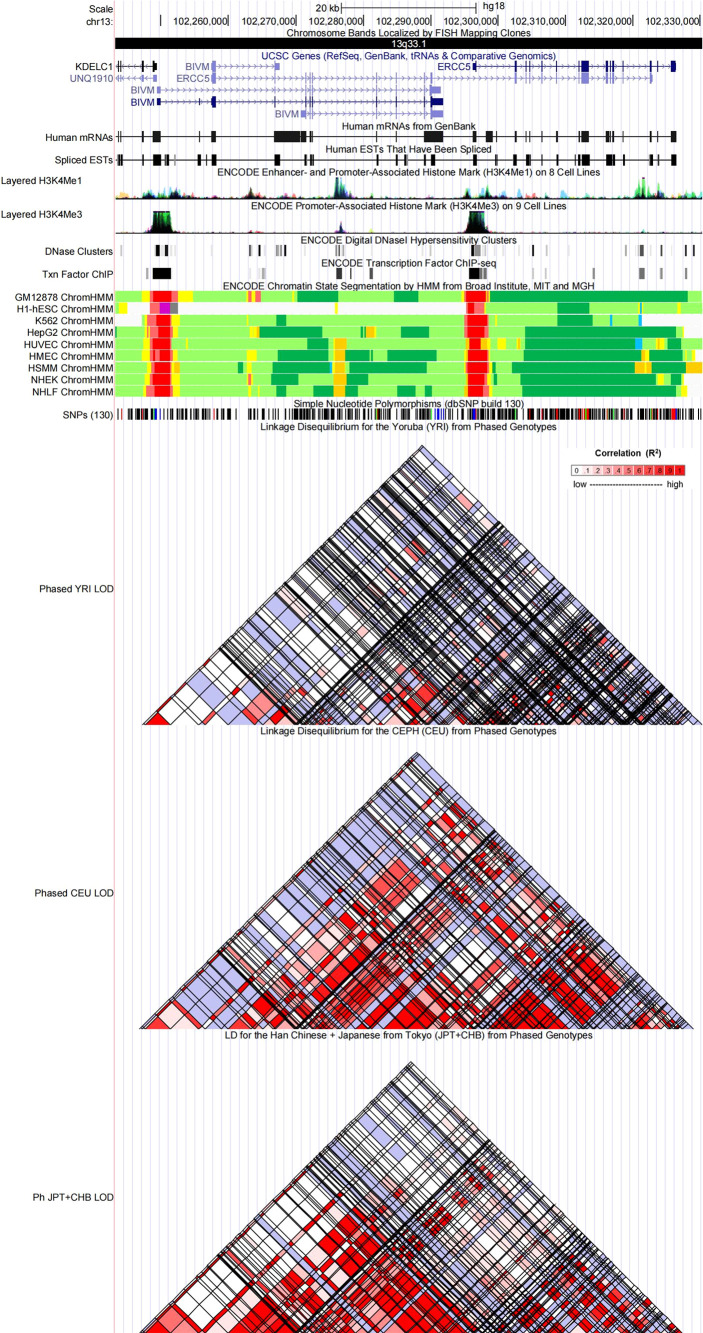
Evidence from the ENCODE data for the regulatory function of variants in 13q33.1 using the UCSC Genome Browser. The plot represents 13q33.1 within a 20-kb window centered on the ERCC5 gene region. Tracks (from top to bottom) in each of the plots are Genome Base Position, Chromosome Bands, UCSC Genes, Human messenger RNAs from GenBank, Human expressed sequence tag (ESTs) That Have Been Spliced, ENCODE Enhancer and Promoter-Associated Histone Mark (H3K4Me1) on 8 Cell Lines, ENCODE Promoter-Associated Histone Mark (H3K4Me3) on 9 Cell Lines, ENCODE Digital DNaseI Hypersensitivity Clusters, ENCODE Transcription Factor ChIP-seq, ENCODE Chromatin State Segmentation by Hidden Markov Model (HMM) from Broad Institute (bright red, active promoter; light red, weak promoter; purple, inactive/poised promoter; orange, strong enhancer; yellow, weak/poised enhancer; blue, insulator; dark green, transcriptional transition/elongation; light green, weak transcribed; gray, polycomb-repressed; light gray, heterochromatin/low signal/repetitive/copy number variation), Simple Nucleotide Polymorphisms (dbSNP build 130), Linkage Disequilibrium for the Yoruba (YRI) from Phased Genotypes, Linkage Disequilibrium for the CEPH (CEU) from Phased Genotypes and LD for the Han Chinese + Japanese from Tokyo (JPT+CHB) from Phased Genotypes.

Referring to the data gained from the Encyclopedia of DNA Elements tool HaploReg v4.1, we analyzed the functional roles of those four variants strongly associated with five cancers (**Table 3**). Results showed that rs744154 mapped to intronic regions, rs2296147 and rs751402 mapped to 5’UTR, and rs17655 was annotated as missense. All these four SNPs might be identified as expression quantitative trait loci (eQTLs) for many genes in various tissue types; two SNPs might be located within the histone modification regions of enhancers and three SNPs in promoters and sites exhibiting DNase I hypersensitivity. Furthermore, we also found that rs2296147 and rs751402 had the alteration in transcription factor binding and all these four variants may affect transcriptional regulatory element activity in this region. Subsequently, as the consequence of the function evaluation using the PolyPhen-2 web server (34), the unique non-synonymous variant rs17655 was qualitatively predicted to be “probably damaging” with a naïve Bayes posterior probability of more than 0.85 (**Figure 2**). In addition, the linkage disequilibrium (LD) plots explained that the regions represented by significant SNPs had distinct genetic structures among European, Asian, and African ancestries (**Figure 3**, **Figure 4**). In addition, the Genotype-Tissue Expression Project revealed that rs744154 is eQTLs for *ERCC4*, whereas rs2296147, rs17655, and rs751402 are eQTLs for *ERCC5*, respectively. Additionally, rs744154 is associated with a decrease in *ERCC4* gene expression in muscle tissues and in *MKL2* gene expression in colon tissues; rs2296147 is associated with a decrease in *BIVM* gene expression and an increase in *METTL21EP* gene expression in esophagus tissues; and rs751402 is associated with a decrease in *BIVM* gene expression in breast tissues and in *ERCC5* gene expression in esophagus tissues **(** **Supplementary Table S6** **).**

**Table 3:** Summary of functional annotations for four single-nucleotide polymorphisms in ERCC4 and ERCC5 with five cancer sites risk (strong epidemiological credibility).

Variant	Gene	Position^a^	Annotation	Promoter histone marks^b^	Enhancer histone marks^c^	DNAse^d^	Proteins bound^e^	Motifs changed^f^
rs744154	ERCC4	13921224	Intronic	17 tissues	17 tissues	6 tissues		Smad
rs2296147	ERCC5	102846025	5’-UTR	24 tissues		53 tissues	17 bound proteins	BDP1
rs17655	ERCC5	102875652	Missense		ESDR, BRN			DEC,GR,Nkx2
rs751402	ERCC5	102845848	5’-UTR	24 tissues		53 tissues	48 bound proteins	E2F,IRC900814,Pou3f2

a The chromosome position is based on NCBI Build 37;

b Histone modification of H3K4me1 and H3K27ac (tissue types: if >3, only the number is included);

c Histone modification of H3K4me3 (tissue types: if >3, only the number is included);

d Levels of DNase I hypersensitivity (tissue types: if >3, only the number is included);

e Alteration in transcription factor binding (disruptions: if >3, only the number is included);

f Alteration in regulatory motif (disruptions: if >3, only the number is included).

## Discussion

The *NER* pathway plays a crucial role in maintaining genomic integrity and preventing carcinogenesis by continuously monitoring and repairing various forms of DNA damage (35). *ERCC4* and *ERCC5* were indispensable component members of the *NER* pathway; numerous studies were conducted to investigate the correlations between the SNPs of *ERCC4* or *ERCC5* and the risk of cancers. However, most previous meta-analyses focused unilaterally on a single SNP and/or an individual cancer type; furthermore, the conclusions of which were inconsonant, resulting from the related small sample size and diversity of population (22, 36–38). To the best of our knowledge, the present study was the first work to comprehensively elucidate whether the studied variants of both *ERCC4* and *ERCC5* were associated with cancer risk and then to evaluate the credibility of significantly epidemiological evidence using the Venice criteria and FPRP tests. We exacted data from a total of 472 datasets in 160 literatures; the relationship among 19 types of cancers and 25 polymorphisms was involved into meta-analyses for assessment. We had 54 associations to be demonstrated as statistically significant, as mentioned above, 10 of which were rated as strong-credibility evidence; moderate and weak credibility were graded to 13 and 31 significant findings. Moreover, the result of functional annotation indicated that these four SNPs (rs744154 in *ERCC4*, rs2296147, and rs17655 and rs751402 in *ERCC5*) with a strong evidence of a significant association might fall in several putative functional regions of *ERCC4* and *ERCC5* genes (**Table 3**). Briefly, our research offers comprehensive epidemiological evidence that common variants of the *ERCC4* and *ERCC5* genes show association with the predisposition of bladder cancer, esophageal cancer, laryngeal cancer, uterine cancer, and gastric cancer.

An obligate heterodimer complex is formed by proteins encoded by *ERCC4* and *ERCC1* genes, which could operate a 5’ incision to the DNA lesion in the irreversible dual-incision process of *NER* (39). The current evidence showed that four SNPs of the *ERCC4* gene (rs744154, rs1800067, rs2276466, and rs1799801) were significantly associated with risk of three cancers (bladder cancer, glioma, and gastric cancer). A former meta-analysis reported that no significant correlation was found between rs744154 and cancer risk (36), but with a larger sample size, we revealed a strong effect of increasing bladder cancer risk with rs744154 in Asian population under allelic and recessive models. In the same population, C allele and GC/CC genotypes were related to a protective effect on the gastric cancer risk compared with the G allele and GG genotype. Wang et al. and Chu et al. indicated that rs744154 was in LD with -357A > C polymorphism in the *ERCC4* promoter, then altered the expression of *ERCC4* mRNA and protein, and finally affected the susceptibility to bladder and gastric cancer (40, 41). Meanwhile, the TC/CC genotypes of rs1799801 polymorphism were proven to be protective factors of gastric cancer in comparison with the TT genotype in overpopulation; however, the statistical significance only appeared in Caucasians when exerting subgroup analysis, not in Asians, considering there was a single dataset of Caucasians. Larger-group studies were needed to further confirm this association. The genetic variants of *ERCC4* were reported to be associated with glioma risk before (42), and in our study, we discovered that rs1800067 and rs2276466 could alter the susceptibility of glioma, but with the reason of the replication of studies, protection from bias and/or FPRP > 0.2, the strength of evidence was moderate or weak, following different genetic models. Further studies are recommended for improving the confidence level of evidence.

The product expressed by *ERCC5* (*XPG*) is an endonuclease, which is mainly in charge of recognizing and cutting DNA lesions on the 3’ side, and the genetic alterations of *ERCC5* might impact the DNA repair capacity as a result of insufficient and loss-of-function proteins, thereby causing the initiation of carcinogenesis (5, 12, 43). As the result of our meta-analysis, seven SNPs were significantly linked to the risk of various types of cancer, including rs17655, rs1047768, rs2094258, rs2296147, rs2228959, rs751402, and rs873601, and we obtained four significant findings (rs17655 in laryngeal and uterine cancer, rs2296147 in esophageal cancer, and rs751402 in gastric cancer) with strong credibility for accumulating epidemiological evidence. The phase 3 of the 1000 Genomes Project (44) (**Supplementary Table S7**) suggested that rs2296147 is in weak LD with rs17655 in Asians (*r* ^2^ = 0.2366), Africans (*r* ^2^ = 0.1354), and Europeans (*r* ^2^ = 0.1354) and is in weak LD with rs751402 in Asians (*r* ^2^ = 0.1470), Africans (*r* ^2^ = 0.0883), and Europeans (*r* ^2^ = 0.2125); rs17655 is in weak LD with rs751402 in Asians (*r* ^2^ = 0.2450), Africans (*r* ^2^ = 0.1948), and Europeans (*r* ^2^ = 0.0679). According to the results, there might exist different causal variants and functional mechanisms in the relationships of variants in the *ERCC5* genes with esophageal cancer, gastric cancer, laryngeal cancer, and uterus and cervical cancer predisposition. Current evidence showed that rs17655 (C vs. G) polymorphism is the most widely studied SNP of *ERCC5*, triggering a replacement of a single amino acid from aspartate to histidine (4). Similar to the result of former research (20), we found that the C allele and CC+ GC genotype could strongly reduce the risk of laryngeal cancer among Asian individuals. Li et al. also pointed out that the variant-related risk may be adjusted by the smoking and alcohol drinking status (20). Apart from laryngeal cancer, the current evidence showed that variant rs17655 was strongly associated with uterine cancer, but conversely, the heterozygotes and homozygotes of this variant were linked to an increased risk of uterine cancer than wild controls. We further observed that this strong evidence of correlation was mainly derived from rs17655 with cervical cancer in the Asian population when stratified by ethnicity and the cancer type; null association was found in Caucasians with endometrial cancer.

In the past couple of years, the relationship between *ERCC5* variants and gastric cancer risk drew much attention of researchers, especially for Chinese investigators. It has been reported that rs873601 (A vs. G), rs2296147 (C vs. T), rs2094258 (T vs. C), rs751402 (G vs. A) were significantly associated with increased or decreased susceptibility of gastric cancer (45–47). What’s interesting is that null significant finding appeared in a study which exploring correlation of all above mentioned SNPs of *ERCC5* and gastric cancer risk (48). With the largest sample size thus far in present study, we acquired that three variants of *ERCC5* were probably involved in carcinogenesis of gastric cancer, rs17655 in Caucasians and rs873601 in Asians was related to an increased risk. rs751402 was strongly associated with a reduced risk of gastric cancer in Asian population. Former study has reported that accumulation of these risk genotypes could reinforce the link between *ERCC5* and gastric cancer risk (48), and some studies figured out that the *Helicobacter pylori* infection may enhance the genetic effect on altered gastric cancer risk (48, 49).

Another strong evidence of association was that rs2296147 (T vs. C) mutant of *ERCC5* could significantly downregulate the susceptibility of esophageal cancer in Asians under allelic and dominant genetic models. A previous study indicated that the locus rs2296147 is located in the 5’UTR, possibly the transcription factor– binding site (TFBS), which hinted that the mutant rs2296147 might affect the transcription process and finally affect the development of malignant tumor (50, 51). However, the specific mechanisms of a potential carcinogenic effect is still unclear; further studies are necessary for finding out whether the rs2296147 locus is the pathogenic SNP.

In this present study, we evaluated the associations between 78 SNPs in *ERCC4* and *ERCC5* and 22 cancer risks based on each SNP extracted from one data source, and then calculated the FPRP values of significant findings; two associations (rs2276466 with gastric cancer risk in Asians, rs2094258 with neuroblastoma in Asians) were affirmed as strong evidence **(** **Supplementary Table S8** **)**. This will provide reference directions for future research.

Our study demonstrated that two SNPs on *ERCC4* and three SNPs on *ERCC5* were observed in a sample of at least 3,000 cases, 3,000 controls, which offers over 89% power to detect an OR of 1.15 in an allelic model for a variant with an MAF of 20%. Further investigations evaluating the following relationships (rs1800067, rs744154 and rs17655 with breast cancer, rs17655 with lung cancer and skin cancer, and rs2094258 and rs2296147 with stomach cancer) will probably not yield meaningful results.

In spite of the largest-sample and comprehensive evaluation of variants related to the risk of cancers, there were several limitations in our study. Firstly, a few literatures might be neglected because we only enrolled studies published in English and the searching stratagem had some drawbacks. Secondly, we could not exact sufficient data for the assessment of the interaction among different variants and the adjustment effect of environment factors like smoking, *H. pylori* infection, and others. Thirdly, we did not carry out a detailed subgroup analysis of cancer types ascribed to the heterogeneity of cancer typing among eligible studies. Fourthly, the excess of significant findings was not further evaluated due to insufficient data. Finally, some of the significant findings were identified with moderate or weak credibility; part of the reasons may be generated from related small samples in the subgroup of ethnicity under different genetic models, so studies with sufficient subgroups are warranted for the validation of our findings.

## Conclusion

In this extensively updated meta-analysis, 11 SNPs were proven to be significantly associated with cancer risk, and four variant-related cancer risks (one for *ERCC4* and three for *ERCC5*) were graded as strong-credibility cumulative epidemiological evidence. These results of high statistical efficacy confirmed once again that SNPs in DNA repair genes play a crucial role in the development of cancer. Therefore, future studies on the pathogenesis of these genes will be conducive to improving the prevention and treatment of cancer.

## Data availability statement

The original contributions presented in the study are included in the article/**Supplementary Material**. Further inquiries can be directed to the corresponding author.

## Author contributions

Specific contributions are as follows: study design: HC, CZ, TL. Data collection: CZ, XL, ZY. Data management and analysis: CZ, LY, CY. Manuscript drafting: XL, CZ. Manuscript review: all. All authors read and approved the final manuscript.

## Funding

This study was supported by funding from the Chongqing Natural Science Foundation (grant No. cstc2020jcyj-msxmX0257).

## Conflict of interest

The authors declare that the research was conducted in the absence of any commercial or financial relationships that could be construed as a potential conflict of interest.

The reviewer XM declare a shared parent affiliation with the author CZ to the handling editor at the time of review.

## Publisher’s note

All claims expressed in this article are solely those of the authors and do not necessarily represent those of their affiliated organizations, or those of the publisher, the editors and the reviewers. Any product that may be evaluated in this article, or claim that may be made by its manufacturer, is not guaranteed or endorsed by the publisher.
