# Supplementary figures and images for: Polymorphisms in ERCC4 and ERCC5 and risk of cancers: Systematic research synopsis, meta-analysis, and epidemiological evidence
Source: Front Oncol. 2022 Aug 11;12:951193. doi: 10.3389/fonc.2022.951193 (PMC9404303; doi:10.3389/fonc.2022.951193)

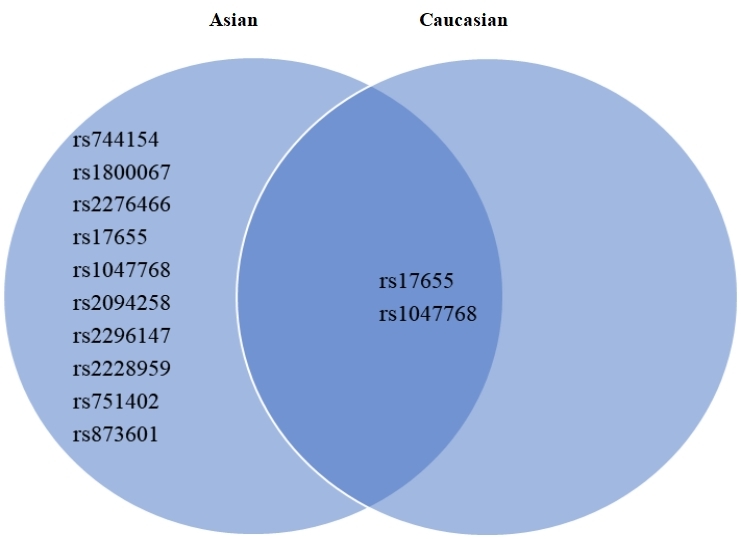

Supplement: Supplementary file 1 [file Image_1.jpeg]

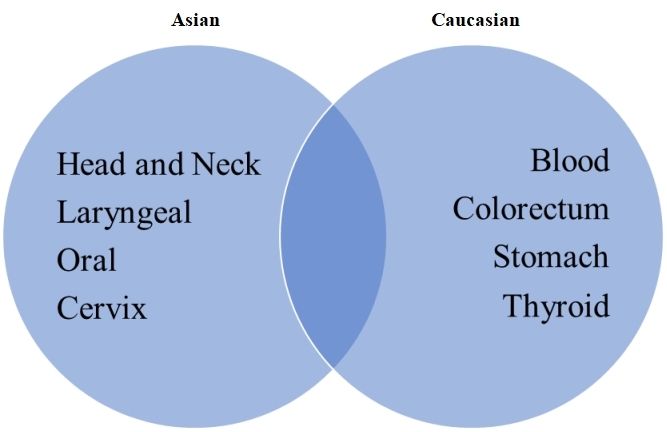

Supplement: Supplementary file 2 [file Image_2.jpeg]
