# Supplementary material for: Polymorphisms in ERCC4 and ERCC5 and risk of cancers: Systematic research synopsis, meta-analysis, and epidemiological evidence
Source: Front Oncol. 2022 Aug 11;12:951193. doi: 10.3389/fonc.2022.951193 (PMC9404303; doi:10.3389/fonc.2022.951193)
Supplement: Supplementary file 4 [file Presentation_2.pdf]

The parameters used for each tool in this study

In statistical analyses part: statistical analyses were conducted utilizing Stata, version 12 (Stata, College Station, TX) (**please see Statistical Analysis part**).

In functional annotation part, as the consequence of the function evaluation using the PolyPhen-2 web server (http://genetics.bwh.harvard.edu/pph2/). The results were assigned into three grade: a mutation was classified as "benign" if its probabilistic score is less than 0.15; a mutation was classified as "possibly damaging" if its probabilistic score ranged from 0.15 to 0.85; a mutation was classified as "probably damaging" if its probabilistic score is above 0.85. In our result showed that the unique non-synonymous variant rs17655 was qualitatively predicted to be "probably damaging" with a naïve Bayes posterior probability of more than 0.85

The underlying functional role of variants on ERCC4 and ERCC5 genes was evaluated with information from the Encyclopedia of DNA Elements (ENCODE) tool HaploReg (v4.1) (https://pubs.broadinstitute.org/mammals/haploreg/haploreg.php) as well as UCSC Genome browser (http://genome.ucsc.edu/). Furthermore, the current work explored genome-wide cis-eQTL data in multiple tissues from the Genotype-Tissue Expression Project (https://gtexportal.org/home) and the Multiple Tissue Human Expression Resource Project databases (http://www.muther.ac.uk/Data.html) in order to reveal whether these genes might explain the observed findings in these loci.

In Discussion part, we attempted to use the Phase 3 of the 1000 Genomes Project (https://ldlink.nci.nih.gov/) to elucide the relationships of variants in the ERCC5 genes with risk of different cancers. Our results found that rs2296147 is in weak LD with rs17655 in Asians (r^2 = 0.2366), Africans (r^2 = 0.1354) and Europeans (r^2 = 0.1354), and is in weak LD with rs751402 in Asians (r^2 = 0.1470), Africans (r^2 = 0.0883) and Europeans (r^2 = 0.2125); rs17655 is in weak LD with rs751402 in Asians (r^2 = 0.2450), Africans (r^2 = 0.1948) and Europeans (r^2 = 0.0679). According to the results, there might exist different causal variants and functional mechanisms in relationships of variants in the *ERCC5* genes with esophageal cancer, gastric cancer, laryngeal cancer, uterus and cervical cancer predisposition.
