# Supplementary material for: Polymorphisms in ERCC4 and ERCC5 and risk of cancers: Systematic research synopsis, meta-analysis, and epidemiological evidence
Source: Front Oncol. 2022 Aug 11;12:951193. doi: 10.3389/fonc.2022.951193 (PMC9404303; doi:10.3389/fonc.2022.951193)
Supplement: Supplementary file 6 [file Table_2.pdf]

Supplementary Table S2: Genetic model with the complete data structure of genetic polymorphism study

Genotype amount			
Genotype type	AA	AB	BB
Case group	a_n	b_n	c_n
Control group	d_n	e_n	f_n

Abbreviation: AA:Wild homozygous; AB: Heterozygous mutant; BB: Mutant homozygous; n: indicating the Nth study.

Description: For a SNP, two alleles, A and B, could be presented. Specifically, A was considered as wild type, meanwhile, B was considered mutant type. Therefore, there may be three genotypes, AA, AB, BB, respectively, in population. Suppose there were three genotypes of the subjects, we could assign a,b,c to AA, AB, BB in case group, and d,e,f to AA, AB, BB in control group, respectively. The table above could offer additional explanation.

In our meta-analyses, the following genetic models were all used to evaluate the associations between genetic variants in miRNAs and cancer risk: 1) Allelic model (i.e. B vs A); 2) Dominant model (BB+BA vs AA); 3) Recessive model (BB vs BA+AA).
