# Supplementary material for: Polymorphisms in ERCC4 and ERCC5 and risk of cancers: Systematic research synopsis, meta-analysis, and epidemiological evidence
Source: Front Oncol. 2022 Aug 11;12:951193. doi: 10.3389/fonc.2022.951193 (PMC9404303; doi:10.3389/fonc.2022.951193)
Supplement: Supplementary file 7 [file Table_3.pdf]

Supplementary Table S3. Characteristics of the included articles

No.	PMID	First author, Year	cancer site	Genotyping	Country/ region	ethnicity	Study design	Case	control	sample size	Gene	variant
1	31568607	Zhang,Y, 2019	Colorectum	RTPCR	China	Asian	CCS	1096	1165	2261	ERCC4	rs6498486 (A>C)
1	31568607	Zhang,Y, 2019	Colorectum	TaqMan	China	Asian	HBCCS	1094	1156	2250	ERCC5	rs17655 (G>C)
2	30161024	Zhu,J, 2018	Kidney	TaqMan	China	Asian	HBCCS	139	531	670	ERCC4	rs2276466 (C>G)
								142	531	673	ERCC5	rs2094258 (C>T)
3	29741112	Lawania,S, 2018	Lung	PCR-RFLP	India	Others	HBCCS	370	377	747	ERCC4	rs316028 (C>T)
								370	370	740	ERCC4	rs254942 (G>A)
								370	370	740	ERCC4	rs1800067 (G>A)
4	29695933	He,J, 2018	Stomach	TaqMan	China	Asian	PBCCS	1141	1173	2314	ERCC4	rs2276466 (C>G)
5	29669843	Zhao,Z, 2018	Ovary	TaqMan	China	Asian	HBCCS	87	356	443	ERCC4	rs2276466 (C>G)
								89	356	445	ERCC5	rs2094258 (C>T)
6	29544698	Zhuo,Z.J, 2018	Brain	TaqMan	China	Asian	CCS	387	812	1199	ERCC4	rs2276466 (C>G)
7	28415781	Campo,R, 2017	Stomach	TaqMan	Spain	Caucasian	CCS	117	600	717	ERCC4	rs1799801 (T>C)
								117	602	719	ERCC4	rs2238463 (C>G)
								116	601	717	ERCC4	rs3136038 (C>T)
								595	594	1189	ERCC4	rs1800067 (G>A)
								248	601	849	ERCC5	rs17655 (G>C)
8	28386271	Dziki,L, 2017	Colorectum	TaqMan	Poland	Caucasian	CCS	309	304	613	ERCC4	rs149364215 (Arg>Ser)
9	27974699	Wang,M, 2017	Prostate	TaqMan	China	Asian	CCS	1004	1055	2059	ERCC4	rs2276464 (G>C)
								1004	1055	2059	ERCC5	rs17655 (G>C)
								1004	1055	2059	ERCC5	rs751402 (A>G)
								1004	1055	2059	ERCC5	rs1047768 (T>C)
10	27768589	He,B.S, 2016	Breast	MassARRAY	China	Asian	CCS	450	430	880	ERCC4	rs1799801 (T>C)
								450	430	880	ERCC5	rs17655 (G>C)
11	27050953	Ying,M.F, 2016	Pancreas	PCR-RFLP	China	Asian	CCS	195	253	448	ERCC4	rs6498486 (A>C)
								195	254	449	ERCC5	rs2094258 (C>T)
12	26617899	Sun,Y, 2015	Laryngeal	PCR-RFLP	China	Asian	HBCCS	271	271	542	ERCC4	rs6498486 (A>C)
								271	271	542	ERCC5	rs2094258 (C>T)

13	26617894	Zhao,F, 2015	Pancreas	PCR-RFLP	China	Asian	HBCCS	246	246	492	ERCC4	rs6498486 (A>C)
								246	246	492	ERCC5	rs873601 (G>A)
14	26146099	Kabzinski,J, 2015	Colorectum	TaqMan	Poland	Caucasian	CCS	146	149	295	ERCC4	rs1799801 (T>C)
15	25730007	Yang,H, 2015	Colorectum	PCR-RFLP	China	Asian	HBCCS	279	316	595	ERCC4	rs2276466 (C>G)
16	25391773	Paszkowska S,K, 2015	Colorectum	TaqMan	Poland	Caucasian	PBCCS	752	1263	2015	ERCC4	rs762521 (G>A)
								463	1330	1793	ERCC5	rs1047768 (T>C)
17	25209577	Pesz,K.A, 2014	Skin	PCR-RFLP	Poland	Caucasian	CCS	96	98	194	ERCC4	rs1800067 (G>A)
								90	100	190	ERCC5	rs17655 (G>C)
18	24938470	Zhou,W.K, 2014	Brain	MassARRAY	China	Asian	HBCCS	225	259	484	ERCC4	rs3136038 (C>T)
								225	262	487	ERCC4	rs1799798 (G>A)
								225	262	487	ERCC4	rs1800067 (G>A)
								225	262	487	ERCC4	rs2276466 (C>G)
19	24933002	Slojewski,M, 2014	Prostate	TaqMan	Poland	Caucasian	CCS	531	772	1303	ERCC4	rs762521 (G>A)
								621	781	1402	ERCC5	rs1047768 (T>C)
20	24861646	Hou,R, 2014	Colorectum	MassARRAY	China	Asian	CCS	204	204	408	ERCC4	rs2276466 (C>G)
								204	204	408	ERCC4	rs6498486 (A>C)
21	24737474	Pei,X.H, 2014	Breast	TaqMan	China	Asian	HBCCS	417	417	834	ERCC4	rs1800067 (G>A)
								417	417	834	ERCC4	rs1799797 (T>C)
								417	417	834	ERCC4	rs2276465 (G>A)
								417	417	834	ERCC4	rs2276466 (C>A)
22	24709955	Liu,Y, 2014	Esophagus	PCR-RFLP	China	Asian	CCS	1524	1524	3048	ERCC4	rs3136038 (C>T)
								1524	1524	3048	ERCC4	rs254942 (G>A)
23	24607854	Steck,S.E, 2014	Colorectum	MassARRAY	USA	African	PBCCS	226	323	549	ERCC4	rs1800067 (G>A)
								224	317	541	ERCC5	rs17655 (G>C)
23	24607854	Steck,S.E, 2014	Colorectum	MassARRAY	USA	Caucasian	PBCCS	304	536	840	ERCC4	rs1800067 (G>A)
								298	532	830	ERCC5	rs17655 (G>C)
24	24582975	Lu,B, 2014	Laryngeal	MassARRAY	China	Asian	HBCCS	176	176	352	ERCC4	rs6498486 (A>C)
								176	176	352	ERCC4	rs2276465 (G>A)
								176	176	352	ERCC5	rs17655 (G>C)
25	24563277	Li,X, 2014	Laryngeal	MassARRAY	China	Asian	HBCCS	210	210	420	ERCC4	rs6498486 (A>C)
								210	210	420	ERCC4	rs2276466 (C>G)
								210	210	420	ERCC4	rs2276465 (G>A)

								211	210	421	ERCC5	rs17655 (G>C)
26	24465539	Kohlhase,S, 2014	Breast	TaqMan	Germany	Caucasian	CCS	932	983	1915	ERCC4	rs1800067 (G>A)
								1937	1229	3166	ERCC4	rs1800067 (G>A)
								829	656	1485	ERCC4	rs1800067 (G>A)
27	23991957	Cheng,H.B, 2013	Brain	MassARRAY	China	Asian	HBCCS	208	236	444	ERCC4	rs3136038 (C>T)
								207	236	443	ERCC4	rs1799798 (G>A)
								207	236	443	ERCC4	rs1800067 (G>A)
								208	236	444	ERCC4	rs6498486 (A>C)
								207	236	443	ERCC4	rs2276465 (G>A)
								207	236	443	ERCC4	rs2276466 (C>G)
28	23982724	Santos,L.S, 2013	Thyroid	TaqMan	Portugal	Caucasian	HBCCS	102	210	312	ERCC4	rs1800067 (G>A)
								105	212	317	ERCC5	rs17655 (G>C)
								106	212	318	ERCC5	rs2227869 (G>C)
29	23911298	Wang,X.F, 2013	Brain	MassARRAY	China	Asian	CCS	330	652	982	ERCC4	rs1800067 (G>A)
								329	652	981	ERCC4	rs6498486 (A>C)
								330	652	982	ERCC4	rs1799797 (T>A)
								331	684	1015	ERCC4	rs3743538 (G>T)
								331	652	983	ERCC4	rs2276465 (G>A)
								330	652	982	ERCC4	rs2276466 (C>A)
30	23909490	Yang,Z, 2013	Breast	MassARRAY	China	Asian	HBCCS	461	504	965	ERCC4	rs2276465 (G>A)
								461	504	965	ERCC4	rs6498486 (A>C)
								461	504	965	ERCC4	rs2276466 (C>G)
31	23720401	Wyss,A.B, 2013	Head and Neck	TaqMan	USA	Caucasian	PBCCS	922	1074	1996	ERCC4	rs3136038 (C>T)
								922	1074	1996	ERCC4	rs1799798 (G>A)
								922	1074	1996	ERCC4	rs744154 (G>C)
								922	1074	1996	ERCC4	rs1800067 (G>A)
								922	1074	1996	ERCC4	rs3136172 (A>G)
31	23720401	Wyss,A.B, 2013	Head and Neck	TaqMan	USA	African	PBCCS	305	251	556	ERCC4	rs3136038 (C>T)
								305	251	556	ERCC4	rs744154 (G>C)
								305	251	556	ERCC4	rs3136085 (G>C)
								305	251	556	ERCC4	rs3136091 (C>G)
								305	251	556	ERCC4	rs3136130 (G>T)

								305	251	556	ERCC4	rs3136172 (A>G)
								305	251	556	ERCC4	rs2020955 (T>C)
32	23679285	Zhang,J.S, 2013	Stomach	TaqMan	China	Asian	HBCCS	331	355	686	ERCC4	rs180067 (G>C)
								331	355	686	ERCC4	rs1799801 (T>C)
								331	355	686	ERCC4	rs2276466 (C>A)
								331	355	686	ERCC4	rs744154 (G>C)
33	23568549	Oliveira,C, 2013	Skin	PCR-RFLP	Brazil	MIixed	HBCCS	146	146	292	ERCC4	rs1799801 (T>C)
34	23537993	Chu,H, 2013	Stomach	PCR-RFLP	China	Asian	HBCCS	350	468	818	ERCC4	rs744154 (G>C)
								350	468	818	ERCC4	rs31870 (A>G)
								350	468	818	ERCC4	rs1799801 (T>C)
35	23436679	Paszkowska S,K, 2013	Skin	MassARRAY	Poland	Caucasian	CCS	626	1263	1889	ERCC4	rs762521 (G>A)
								633	1330	1963	ERCC5	rs1047768 (T>C)
								650	1356	2006	ERCC5	rs1047769 (A>G)
								640	1358	1998	ERCC5	rs17655 (G>C)
								636	1332	1968	ERCC5	rs2227869 (G>C)
								519	649	1168	ERCC5	rs4150360 (C>T)
36	23166636	He,J, 2012	Stomach	TaqMan	China	Asian	CCS	1125	1196	2321	ERCC4	rs2276466 (C>G)
								1125	1196	2321	ERCC4	rs6498486 (A>C)
37	22848636	Yu,H, 2012	Head and Neck	PCR-RFLP	USA	Caucasian	HBCCS	1040	1046	2086	ERCC4	rs2276466 (C>G)
								1040	1046	2086	ERCC4	rs1800067 (G>A)
								1039	1042	2081	ERCC4	rs1799798 (G>A)
								1040	1046	2086	ERCC4	rs3136038 (C>T)
38	22493747	Sakoda,L.C, 2012	Lung	TaqMan	USA	Caucasian	CCS	744	1477	2221	ERCC4	rs1799797 (T>A)
								743	1477	2220	ERCC4	rs1800067 (G>A)
								744	1477	2221	ERCC4	rs3136166 (T>G)
								742	1476	2218	ERCC4	rs1799801 (T>C)
								743	1476	2219	ERCC5	rs2018836 (A>G)
								741	1471	2212	ERCC5	rs2296147 (T>C)
								743	1474	2217	ERCC5	rs7325708 (C>G)
								742	1474	2216	ERCC5	rs1047768 (T>C)
								740	1471	2211	ERCC5	rs1047769 (A>G)
								744	1474	2218	ERCC5	rs2227869 (G>C)

								742	1474	2216	ERCC5	rs3759500 (C>T)
								742	1475	2217	ERCC5	rs4150351 (A>C)
								742	1475	2217	ERCC5	rs4150355 (C>T)
								744	1477	2221	ERCC5	rs732321 (A>C)
								744	1474	2218	ERCC5	rs4150386 (A>C)
								744	1476	2220	ERCC5	rs17655 (G>C)
								742	1474	2216	ERCC5	rs873601 (G>A)
								741	1476	2217	ERCC5	rs4150393 (A>G)
39	22374244	Zhou,R.M, 2012	Esophagus	PCR-RFLP	China	Asian	CCS	389	778	1167	ERCC4	rs6498486 (A>C)
								262	524	786	ERCC4	rs6498486 (A>C)
40	21750170	Doherty,J.A, 2011	uterus	TaqMan	USA	Caucasian	PBCCS	713	717	1430	ERCC4	rs3136064 (C>T)
								703	714	1417	ERCC4	rs1800067 (G>A)
								708	715	1423	ERCC4	rs3136215 (T>C)
								722	727	1449	ERCC4	rs1799801 (T>C)
40	21750170	Doherty,J.A, 2011	Uterus	SNaPshot	USA	Caucasian	PBCCS	715	710	1425	ERCC5	rs2296147 (T>C)
								680	699	1379	ERCC5	rs4150261 (G>A)
								681	700	1381	ERCC5	rs4150276 (T>A)
								702	679	1381	ERCC5	rs3818356 (G>A)
								702	681	1383	ERCC5	rs4150351 (A>C)
								702	680	1382	ERCC5	rs4150355 (C>T)
								726	722	1448	ERCC5	rs4150375 (A>G)
								702	680	1382	ERCC5	rs4150383 (G>A)
								664	646	1310	ERCC5	rs4150386 (A>C)
								703	714	1417	ERCC5	rs17655 (G>C)
								702	681	1383	ERCC5	rs4150393 (A>G)
41	21622940	Roberts,M.R, 2011	Breast	MassARRAY	USA	Caucasian	PBCCS	1063	1913	2976	ERCC4	rs1799801 (T>C)
42	21559836	Gil,J, 2012	Colorectum	PCR-RFLP	Poland	Caucasian	CCS	133	98	231	ERCC4	rs1800067 (G>A)
								132	100	232	ERCC5	rs17655 (G>C)
43	20183911	Ming Shiean,H, 2010	Breast	TaqMan	Taiwan	Asian	HBCCS	401	533	934	ERCC4	rs2020955 (T>C)
								401	531	932	ERCC5	rs17655 (G>C)
44	20150366	Rajaraman,P, 2010	Brain	TaqMan	USA	Caucasian	HBCCS	337	471	808	ERCC4	rs1800067 (G>A)
								124	471	595	ERCC4	rs1800067 (G>A)

								65	471	536	ERCC4	rs1800067 (G>A)
								332	445	777	ERCC4	rs2020955 (T>C)
								121	445	566	ERCC4	rs2020955 (T>C)
								62	445	507	ERCC4	rs2020955 (T>C)
								342	468	810	ERCC5	rs17655 (G>C)
								121	468	589	ERCC5	rs17655 (G>C)
								65	468	533	ERCC5	rs17655 (G>C)
45	20062074	Wang,M, 2010	Bladder	PCR-RFLP	China	Asian	HBCCS	234	250	484	ERCC4	rs744154 (G>C)
								234	250	484	ERCC4	rs31870 (A>G)
								234	250	484	ERCC4	rs1799801 (T>C)
								130	150	280	ERCC4	rs744154 (G>C)
								130	150	280	ERCC4	rs6498486 (A>C)
46	19902366	Agalliu,I, 2010	Prostate	SNPlex	USA	Caucasian	PBCCS	1259	1243	2502	ERCC4	rs1799801 (T>C)
								1221	1219	2440	ERCC4	rs1800067 (G>A)
46	19902366	Agalliu,I, 2010	Prostate	SNPlex	USA	African	PBCCS	145	79	224	ERCC4	rs1799801 (T>C)
								144	81	225	ERCC4	rs1800067 (G>A)
47	19444904	Abbasi,R, 2009	Laryngeal	PCR-RFLP	Germany	Caucasian	PBCCS	248	647	895	ERCC4	rs1800067 (G>A)
								248	647	895	ERCC5	rs17655 (G>C)
								248	647	895	ERCC5	rs1047768 (T>C)
48	19270000	Pan,J, 2009	Esophagus	TaqMan	USA	Caucasian	CCS	384	453	837	ERCC4	rs2020955 (T>C)
								382	457	839	ERCC5	rs17655 (G>C)
49	19029193	Joshi,A.D, 2009	Colorectum	TaqMan	USA	Caucasian	PBCCS	129	148	277	ERCC4	rs1800067 (G>A)
								176	209	385	ERCC4	rs1800067 (G>A)
50	18767034	Rajaraman,P, 2008	Breast	TaqMan	USA	Caucasian	CCS	838	1069	1907	ERCC4	rs1800067 (G>A)
								821	1071	1892	ERCC4	rs1800124 (A>G)
								852	1082	1934	ERCC5	rs1047769 (A>G)
								819	1079	1898	ERCC5	rs17655 (G>C)
51	18711149	Lin,J, 2008	Kidney	TaqMan	USA	Caucasian	PBCCS	319	326	645	ERCC4	rs2020955 (T>C)
								323	335	658	ERCC5	rs17655 (G>C)
52	18709642	Chang,J.S, 2008	Lung	TaqMan	USA	Latino	CCS	113	299	412	ERCC4	rs1800067 (G>A)
								113	299	412	ERCC5	rs17655 (G>C)
52	18709642	Chang,J.S, 2008	Lung	TaqMan	USA	African	CCS	255	280	535	ERCC5	rs17655 (G>C)

53	18551366	Han,J, 2009	Breast	TaqMan	USA	Caucasian	PBCCS	238	473	711	ERCC4	rs11648736 (G>A)
								238	475	713	ERCC4	rs4781560 (T>C)
								235	471	706	ERCC4	rs3136130 (G>T)
								236	472	708	ERCC4	rs1646332 (C>A)
								237	475	712	ERCC4	rs11649492 (G>A/C)
								235	473	708	ERCC4	rs3136112 (C>T)
								238	473	711	ERCC4	rs3136064 (C>T)
								239	474	713	ERCC4	rs3136189 (T>C)
								236	466	702	ERCC4	rs3136038 (C>T)
53	18551366	Han,J, 2009	Breast	BeadArray	USA	Mixed	CCS	234	469	703	ERCC5	rs4150350 (G>A)
								235	470	705	ERCC5	rs1047769 (A>G)
								237	473	710	ERCC5	rs4150282 (C>T)
								239	472	711	ERCC5	rs17655 (G>C)
								238	473	711	ERCC5	rs4150351 (A>C)
								237	471	708	ERCC5	rs4743 (C>G)
								235	471	706	ERCC5	rs4150260 (G>A)
								239	471	710	ERCC5	rs2018836 (A>G)
								239	474	713	ERCC5	rs4150355 (C>T)
								239	474	713	ERCC5	rs1408047 (C>T)
								232	462	694	ERCC5	rs4150386 (A>C)
								238	473	711	ERCC5	rs4771435 (A>G)
								233	469	702	ERCC5	rs9557945 (T>A)
								236	471	707	ERCC5	rs873601 (G>A)
								236	473	709	ERCC5	rs7325496 (C>A/G)
								238	471	709	ERCC5	rs2227869 (G>C)
54	18544627	McWilliams,R.R, 2008	Pancreas	SNPstream	USA	Caucasian	CCS	470	596	1066	ERCC4	rs1800067 (G>A)
								468	593	1061	ERCC4	rs2020955 (T>C)
55	18068852	Shao,M, 2008	Lung	TaqMan	China	Asian	CCS	971	973	1944	ERCC4	rs3136038 (C>T)
								912	942	1854	ERCC4	rs1799798 (G>A)
								996	989	1985	ERCC4	rs2238463 (C>G)
56	18026184	Hooker,S, 2008	Prostate	MassARRAY	USA	African	CCS	254	301	555	ERCC4	rs2020955 (T>C)

								252	301	553	ERCC4	rs1799801 (T>C)
								256	301	557	ERCC5	rs2296148 (C>T)
								254	304	558	ERCC5	rs4150313 (A>G)
								254	301	555	ERCC5	rs2227869 (G>C)
								254	301	555	ERCC5	rs17655 (G>C)
57	17932351	Crew,K.D, 2007	Breast	HMLDTMS	USA	Caucasian	PBCCS	1018	1065	2083	ERCC4	rs1800067 (G>A)
								799	1051	1850	ERCC5	rs17655 (G>C)
58	17728339	Chen,M, 2007	Bladder	TaqMan	USA	Caucasian	CCS	607	597	1204	ERCC4	rs2020955 (T>C)
59	17210993	Povey,J.E, 2007	Skin	PCR-RFLP	UK	Caucasian	PBCCS	506	441	947	ERCC4	rs1799801 (T>C)
								507	441	948	ERCC5	rs17655 (G>C)
60	16823510	Jorgensen,T.J, 2007	Breast	TaqMan	USA	Caucasian	PBCCS	259	275	534	ERCC4	rs1800067 (G>A)
								264	275	539	ERCC5	rs17655 (G>C)
61	16609022	Moreno,V, 2006	Colorectum	APEX	Spain	Caucasian	HBCCS	362	323	685	ERCC4	rs1799802 (C>T)
								360	323	683	ERCC4	rs1800067 (G>A)
								351	320	671	ERCC5	rs1047768 (T>C)
62	16537713	Garcia Closas,M, 2006	Bladder	TaqMan	Spain	Caucasian	CCS	1102	1025	2127	ERCC4	rs1800067 (G>A)
								1083	1066	2149	ERCC4	rs1799799 (T>C)
								1066	1001	2067	ERCC4	rs2020955 (T>C)
								1091	1019	2110	ERCC4	rs1799801 (T>C)
								1103	1094	2197	ERCC5	rs1047768 (T>C)
								1077	1068	2145	ERCC5	rs1047769 (A>G)
								1143	1136	2279	ERCC5	rs2227869 (G>C)
								1141	1136	2277	ERCC5	rs17655 (G>C)
63	16492920	Huang,W.Y, 2006	Colorectum	TaqMan	USA	Caucasian	PBCCS	691	702	1393	ERCC4	rs1799802 (C>T)
								703	716	1419	ERCC4	rs1800067 (G>A)
								670	687	1357	ERCC5	rs1047769 (A>G)
								651	662	1313	ERCC5	rs2227869 (G>C)
								679	697	1376	ERCC5	rs17655 (G>C)
64	16465622	Wu,X, 2006	Bladder	TaqMan	USA	Caucasian	HBCCS	607	597	1204	ERCC4	rs2020955 (T>C)
								615	600	1215	ERCC5	rs17655 (G>C)
65	16393248	Sugimura,T, 2006	Oral	PCR	Japan	Asian	CCS	122	241	363	ERCC4	rs1799797 (T>A)
65	16393248	Sugimura,T, 2006	Oral	PCR-RFLP	Japan	Asian	CCS	122	241	363	ERCC5	rs17655 (G>C)

66	16284380	Matullo,G, 2005	Bladder	SNaPshot	Italy	Caucasian	HBCCS	204	213	417	ERCC4	rs1799801 (T>C)
								289	291	580	ERCC4	rs1800124 (A>G)
67	16284373	Weiss,J.M, 2005	uterus	SNaPshot	USA	Caucasian	PBCCS	371	420	791	ERCC4	rs1800067 (G>A)
								371	420	791	ERCC4	rs1799801 (T>C)
								371	420	791	ERCC5	rs17655 (G>C)
68	16195237	Zienolddiny,S, 2006	Lung	APEX	Norway	Caucasian	PBCCS	337	409	746	ERCC4	rs1799802 (C>T)
								224	200	424	ERCC4	rs1800067 (G>A)
								316	373	689	ERCC5	rs1047768 (T>C)
69	15886521	Lee,S.A, 2005	Breast	DASH	Korea	Asian	HBCCS	386	336	722	ERCC4	rs1799801 (T>C)
70	14652281	Smith,T.R, 2003	Breast	PCR-RFLP	USA	Caucasian	HBCCS	253	268	521	ERCC4	rs1800067 (G>A)
71	23659246	Torres,S.M, 2013	Skin	Golden Gate	USA	Caucasian	PBCCS	892	763	1655	ERCC4	rs1799800 (G>A)
								893	766	1659	ERCC4	rs1800067 (G>A)
								893	766	1659	ERCC4	rs744154 (G>C)
								884	756	1640	ERCC4	rs9302507 (C>T)
72	23415627	Gong,Y, 2013	Stomach	PCR-RFLP	China	Asian	CCS	400	400	800	ERCC4	rs6498486 (A>C)
								378	378	756	ERCC4	rs254942 (G>A)
73	19423537	Gaudet,M.M, 2009	Breast	Quantitative PCR	Multicenter	Asian	CCS	2700	2104	4804	ERCC4	rs744154 (G>C)
								651	571	1222	ERCC4	rs744154 (G>C)
								25743	29074	54817	ERCC4	rs744154 (G>C)
74	24634177	Gao K, 2014	Brain	MassARRAY	China	Asian	HBCCS	325	376	701	ERCC4	rs1799801 (T>C)
								325	376	701	ERCC4	rs1800067 (G>A)
75	21641795	Ibarrola Villava M, 2011	Skin	MassARRAY	Spain	Caucasian	CCS	600	378	978	ERCC4	rs1800067 (G>A)
								600	378	978	ERCC4	rs1799797 (T>A)
								600	378	978	ERCC4	rs1799801 (T>C)
								599	380	979	ERCC5	rs17655 (G>C)
76	18701435	Tasha R.Smith, 2008	Breast	MassARRAY	USA	Caucasian	CCS	324	406	730	ERCC4	rs1800067 (G>A)
76	18701435	Tasha R.Smith, 2008	Breast	MassARRAY	USA	African	CCS	53	75	128	ERCC4	rs1800067 (G>A)
76	18701435	Tasha R.Smith, 2008	Breast	MassARRAY	USA	Caucasian	HBCCS	320	408	728	ERCC5	rs17655 (G>C)
76	18701435	Tasha R.Smith, 2008	Breast	MassARRAY	USA	African	HBCCS	52	75	127	ERCC5	rs17655 (G>C)

77	16399771	Leah E.M, 2006	Breast	TaqMan	USA	Caucasian	PBCCS	1246	1133	2379	ERCC4	rs1800067 (G>A)
								249	250	499	ERCC4	rs2020955 (T>C)
								1249	1133	2382	ERCC5	rs17655 (G>C)
77	16399771	Leah E.M, 2006	Breast	TaqMan	USA	African	PBCCS	757	673	1430	ERCC4	rs1800067 (G>A)
								752	674	1426	ERCC4	rs2020955 (T>C)
								757	674	1431	ERCC5	rs17655 (G>C)
78	15849729	Shen M, 2005	Lung	RTPCR	China	Asian	PBCCS	117	111	228	ERCC4	rs1799801 (T>C)
								118	112	230	ERCC5	rs1047768 (T>C)
								118	111	229	ERCC5	rs2227869 (G>C)
								106	99	205	ERCC5	rs2228959 (C>A)
								116	109	225	ERCC5	rs17655 (G>C)
79	11059748	Winsey,S.L, 2000	Skin	PCR-SSP	UK	Caucasian	PBCCS	125	211	336	ERCC4	rs1799797 (T>A)
								125	211	336	ERCC4	rs1799801 (T>C)
80	26967386	Wang,B, 2016	Liver	MassARRAY	China	Asian	PBCCS	168	489	657	ERCC4	rs254942 (G>A)
								169	500	669	ERCC4	rs1799801 (T>C)
								169	497	666	ERCC4	rs2276464 (G>C)
								538	893	1431	ERCC5	rs873601 (G>A)
81	16857995	Deirdre A H, 2006	Blood	TaqMan	USA	Caucasian	PBCCS	662	553	1215	ERCC4	rs1799802 (C>T)
81	16857995	Deirdre A H. 2006	Blood	TaqMan	USA	Mixed	PBCCS	1101	923	2024	ERCC5	rs17655 (G>C)
82	19920816	A Osorio, 2009	Breast	TaqMan	Multicen ter	Caucasian	CCS	8088	6952	15040	ERCC4	rs744154 (G>C)
83	26946191	Mordukhovich,I, 2016	Breast	HMLDTMS	USA	Caucasian	PBCCS	861	925	1786	ERCC4	rs1800067 (G>A)
								852	911	1763	ERCC5	rs17655 (G>C)
84	23534771	Luo,K.Q, 2013	Brain	MassARRAY	China	Asian	HBCCS	297	415	712	ERCC4	rs3734091 (G>T)
								297	415	712	ERCC5	rs17655 (G>C)
85	31988591	Li,Y.K, 2020	Colorectum	KASPCR	China	Asian	CCS	839	845	1684	ERCC5	rs1047768 (T>C)
								843	841	1684	ERCC5	rs2094258 (C>T)
								841	851	1692	ERCC5	rs2228959 (C>A)
								844	847	1691	ERCC5	rs2296147 (T>C)
								842	837	1679	ERCC5	rs873601 (G>A)
86	31584889	Minina,V.I, 2019	Lung	KASPCR	Russia	Caucasian	CCS	340	335	675	ERCC5	rs17655 (G>C)
87	31528641	Shakil,Malik S, 2019	Breast	Tetra	Pakistan	Asian	CCS	100	75	175	ERCC5	rs1047768 (T>C)

				ARMS-PCR								
88	31450912	Nigam,K, 2019	Oral	PCR-RFLP	India	Others	CCS	293	288	581	ERCC5	rs17655 (G>C)
89	31374908	Santos,L.S, 2019	Thyroid	PCR-RFLP	Portugal	Caucasian	HBCCS	423	211	634	ERCC5	rs2227869 (G>C)
90	30899401	Su,J 2019	Colorectum	TaqMan	China	Asian	PBCCS	1019	1036	2055	ERCC5	rs17655 (G>C)
91	30522358	Lawania,S, 2019	Lung	PCR-RFLP	India	Others	HBCCS	370	370	740	ERCC5	rs2228959 (C>A)
								370	370	740	ERCC5	rs17655 (G>C)
92	30245779	Pongsavee,M, 2018	Breast	RT-qPCR	Thailand	Asian	CCS	155	122	277	ERCC5	rs751402 (A>G)
								142	531	673	ERCC5	rs751402 (A>G)
								142	531	673	ERCC5	rs2296147 (T>C)
								142	531	673	ERCC5	rs1047768 (T>C)
								142	531	673	ERCC5	rs873601 (G>A)
93	29732643	Zhang,Z, 2018	Stomach	PCR-RFLP	China	Asian	HBCCS	382	436	818	ERCC5	rs2094258 (C>T)
								89	356	445	ERCC5	rs751402 (A>G)
								89	356	445	ERCC5	rs2296147 (T>C)
								89	356	445	ERCC5	rs1047768 (T>C)
								89	356	445	ERCC5	rs873601 (G>A)
94	29434449	Sang,L, 2018	Stomach	MassARRAY	China	Asian	CCS	450	535	985	ERCC5	rs1047768 (T>C)
								450	535	985	ERCC5	rs2094258 (C>T)
								450	535	985	ERCC5	rs2228959 (C>A)
								450	535	985	ERCC5	rs4150291 (A>T)
								450	535	985	ERCC5	rs4150383 (G>A)
								427	509	936	ERCC5	rs751402 (A>G)
								450	535	985	ERCC5	rs873601 (G>A)
95	29254785	Perez R,C, 2018	Lung	TaqMan	Spain	Caucasian	CCS	174	298	472	ERCC5	rs1047768 (T>C)
								174	298	472	ERCC5	rs17655 (G>C)
								174	298	472	ERCC5	rs2296147 (T>C)
								174	298	472	ERCC5	rs2094258 (C>T)
								174	298	472	ERCC5	rs873601 (G>A)
96	28952217	Zhang,C, 2017	Esophagus	TaqMan	China	Asian	HBCCS	557	1503	2060	ERCC5	rs2296147 (T>C)
								579	1503	2082	ERCC5	rs873601 (G>A)
								557	1503	2060	ERCC5	rs2094258 (C>T)
97	28920307	Li,X, 2012	Lung	AllGlo probe	China	Asian	HBCCS	385	208	593	ERCC5	rs17655 (G>C)

				assay								
98	28499365	Lonjou,C, 2017	Thyroid	Illumina	Belarus	Caucasian	CCS	75	254	329	ERCC5	rs1047768 (T>C)
								74	251	325	ERCC5	rs17655 (G>C)
99	27929383	Hua,R.X ,2016	Stomach	TaqMan	China	Asian	CCS	1142	1173	2315	ERCC5	rs2094258 (C>T)
								1142	1173	2315	ERCC5	rs751402 (A>G)
								1142	1173	2315	ERCC5	rs2296147 (T>C)
								1142	1173	2315	ERCC5	rs1047768 (T>C)
								1142	1173	2315	ERCC5	rs873601 (G>A)
100	27706622	Yang,L.Q, 2016	Stomach	PCR-RFLP	China	Asian	HBCCS	155	246	401	ERCC5	rs2094258 (C>T)
								155	246	401	ERCC5	rs751402 (A>G)
101	27698911	Hua,R.X, 2016	Colorectum	TaqMan	China	Asian	CCS	1901	1976	3877	ERCC5	rs2094258 (C>T)
								1900	1976	3876	ERCC5	rs751402 (A>G)
								1901	1976	3877	ERCC5	rs2296147 (T>C)
								1901	1976	3877	ERCC5	rs1047768 (T>C)
								1901	1976	3877	ERCC5	rs873601 (G>A)
102	27323183	Guo,B.W, 2016	Stomach	PCR-RFLP	China	Asian	HBCCS	142	274	416	ERCC5	rs17655 (G>C)
								142	274	416	ERCC5	rs751402 (A>G)
103	27323165	Feng,Y.B, 2016	Stomach	PCR-RFLP	China	Asian	HBCCS	177	238	415	ERCC5	rs2094258 (C>T)
								177	236	413	ERCC5	rs751402 (A>G)
								177	237	414	ERCC5	rs17655 (G>C)
104	27323158	Lu,J.J, 2016	Stomach	PCR-RFLP	China	Asian	HBCCS	184	206	390	ERCC5	rs2094258 (C>T)
								184	206	390	ERCC5	rs751402 (A>G)
105	27323134	Ma,S.H, 2016	Breast	PCR-RFLP	China	Asian	HBCCS	320	238	558	ERCC5	rs2094258 (C>T)
								320	236	556	ERCC5	rs751402 (A>G)
								320	237	557	ERCC5	rs17655 (G>C)
106	27228234	Zhou,R.M, 2016	Stomach	PCR-LDR	China	Asian	CCS	431	432	863	ERCC5	rs751402 (A>G)
								431	432	863	ERCC5	rs873601 (G>A)
107	27051028	Li,R.J ,2016	Stomach	PCR-RFLP	China	Asian	HBCCS	216	216	432	ERCC5	rs1047768 (T>C)
								216	216	432	ERCC5	rs751402 (A>G)
								216	216	432	ERCC5	rs17655 (G>C)
108	27019310	He,J, 2016	Nervous tissue	TaqMan	China	Asian	CCS	248	531	779	ERCC5	rs2094258 (C>T)
								248	531	779	ERCC5	rs751402 (A>G)

								248	531	779	ERCC5	rs2296147 (T>C)
								248	531	779	ERCC5	rs1047768 (T>C)
								248	531	779	ERCC5	rs873601 (G>A)
109	26820236	Chen,Y.Z 2016	Stomach	TaqMan	China	Asian	HBCCS	692	771	1463	ERCC5	rs2094258 (C>T)
								692	771	1463	ERCC5	rs751402 (A>G)
								692	771	1463	ERCC5	rs2296147 (T>C)
								692	771	1463	ERCC5	rs873601 (G>A)
110	26130668	Joo,J, 2016	Uterus	TaqMan	Korea	Asian	HBCCS	478	922	1400	ERCC5	rs17655 (G>C)
								478	922	1400	ERCC5	rs9557929 (G>C)
								478	922	1400	ERCC5	rs2094258 (C>T)
								478	922	1400	ERCC5	rs751402 (A>G)
								478	922	1400	ERCC5	rs9557946 (T>A)
111	26045839	Na,N, 2015	Breast	PCR-RFLP	China	Asian	HBCCS	325	325	650	ERCC5	rs1047768 (T>C)
								325	325	650	ERCC5	rs2094258 (C>T)
								325	325	650	ERCC5	rs2296147 (T>C)
								325	325	650	ERCC5	rs751402 (A>G)
								325	325	650	ERCC5	rs873601 (G>A)
112	25835182	Kabzinski,J, 2015	Colorectum	TaqMan	Poland	Caucasian	CCS	234	238	472	ERCC5	rs17655 (G>C)
113	25644244	Wang,H, 2016	Breast	PCR-RFLP	China	Asian	HBCCS	101	101	202	ERCC5	rs17655 (G>C)
								101	101	202	ERCC5	rs751402 (A>G)
114	25400036	Bahceci,A 2015	Blood	Tm Shift RT PCR	USA	Caucasian	CCS	93	96	189	ERCC5	rs17655 (G>C)
								460	1356	1816	ERCC5	rs1047769 (A>G)
								733	1358	2091	ERCC5	rs17655 (G>C)
								429	1332	1761	ERCC5	rs2227869 (G>C)
								651	649	1300	ERCC5	rs4150360 (C>T)
115	25355595	Sun,K, 2015	Colorectum	PCR-RFLP	China	Asian	PBCCS	890	910	1800	ERCC5	rs17655 (G>C)
116	25311495	Douzi,K, 2015	Blood	PCR-RFLP	Tunisia	African	CCS	206	206	412	ERCC5	rs17655 (G>C)
								615	783	1398	ERCC5	rs1047769 (A>G)
								571	782	1353	ERCC5	rs2227869 (G>C)
								636	778	1414	ERCC5	rs17655 (G>C)
117	24648912	Fabisiewicz,A, 2013	Blood	TaqMan	Poland	Caucasian	CCS	182	193	375	ERCC5	rs17655 (G>C)

118	24615090	Zhang,X.J, 2014	Prostate	MassARRAY	China	Asian	HBCCS	230	238	468	ERCC5	rs2296147 (T>C)
								229	238	467	ERCC5	rs2094258 (C>T)
								210	210	420	ERCC5	rs1047768 (T>C)
119	24289586	Yang,,B 2013	Prostate	MassARRAY	China	Asian	HBCCS	229	238	467	ERCC5	rs2296147 (T>C)
								229	238	467	ERCC5	rs2094258 (C>T)
120	23818366	Ruiz Cosano,J, 2013	Blood	TaqMan	Spain	Caucasian	CCS	213	214	427	ERCC5	rs17655 (G>C)
121	23464443	Yang,W.G, 2012	Stomach	TaqMan	China	Asian	HBCCS	337	347	684	ERCC5	rs2296147 (T>C)
								337	347	684	ERCC5	rs2094258 (C>T)
								337	346	683	ERCC5	rs873601 (G>A)
122	23246108	Wen,H, 2013	Bladder	TaqMan	China	Asian	HBCCS	112	278	390	ERCC5	rs17655 (G>C)
123	22982416	Duan,Z, 2012	Stomach	PCR-RFLP	China	Asian	PBCCS	400	400	800	ERCC5	rs751402 (A>G)
								403	403	806	ERCC5	rs2296147 (T>C)
124	22981091	Zavras,A.I, 2012	Oral	TaqMan	Taiwan	Asian	HBCCS	239	336	575	ERCC5	rs751402 (A>G)
125	22866149	Yoon,A.J, 2011	Liver	TaqMan	Taiwan	Asian	HBCCS	96	336	432	ERCC5	rs751402 (A>G)
126	22848513	Zhu,M.L, 2012	Esophagus	TaqMan	China	Asian	HBCCS	1115	1117	2232	ERCC5	rs2296147 (T>C)
								1115	1117	2232	ERCC5	rs2094258 (C>T)
								1115	1117	2232	ERCC5	rs873601 (G>A)
127	22371296	He,J, 2012	Stomach	TaqMan	China	Asian	CCS	1125	1196	2321	ERCC5	rs2094258 (C>T)
								1125	1196	2321	ERCC5	rs2296147 (T>C)
								1125	1196	2321	ERCC5	rs873601 (G>A)
128	22271435	Cincin,Z.B, 2012	Uterus	PCR-RFLP	Turkey	Caucasian	HBCCS	104	158	262	ERCC5	rs17655 (G>C)
129	22213216	Liu,D, 2012	Colorectum	PCR-RFLP	China	Asian	PBCCS	1028	1085	2113	ERCC5	rs17655 (G>C)
130	22108238	Ma,H, 2012	Head and Neck	PCR-RFLP	USA	Caucasian	HBCCS	1038	1053	2091	ERCC5	rs2094258 (C>T)
								1056	1065	2121	ERCC5	rs2296147 (T>C)
								1057	1064	2121	ERCC5	rs4771436 (T>G)
								1059	1065	2124	ERCC5	rs1047768 (T>C)
								1059	1066	2125	ERCC5	rs2227869 (G>C)
								1057	1065	2122	ERCC5	rs4150351 (A>C)
								1054	1061	2115	ERCC5	rs4150355 (C>T)
								1059	1063	2122	ERCC5	rs4150383 (G>A)
								1059	1066	2125	ERCC5	rs4150386 (A>C)
								1059	1066	2125	ERCC5	rs17655 (G>C)

								1058	1066	2124	ERCC5	rs873601 (G>A)
								1059	1066	2125	ERCC5	rs4150393 (A>G)
131	21733660	Goncalves,F.T, 2011	Skin	PCR-RFLP	Brazil	Caucasian	HBCCS	192	208	400	ERCC5	rs17655 (G>C)
132	21670956	Berhane,N, 2012	Prostate	PCR-RFLP	India	Others	PBCCS	150	150	300	ERCC5	rs17655 (G>C)
133	21647780	Rouissi,K, 2011	Bladder	PCR-RFLP	Tunisia	African	CCS	125	125	250	ERCC5	rs17655 (G>C)
134	21561390	Canbay,E, 2011	Colorectum	PCR-RFLP	Turkey	Caucasian	CCS	79	247	326	ERCC5	rs17655 (G>C)
135	21426550	Rouissi,K, 2011	Bladder	PCR-RFLP	Tunisia	African	CCS	193	193	386	ERCC5	rs17655 (G>C)
136	20530453	Canbay,E, 2010	Stomach	PCR-RFLP	Turkey	Caucasian	CCS	40	247	287	ERCC5	rs17655 (G>C)
137	19414392	Narter,K.F, 2009	Bladder	PCR-RFLP	Turkey	Caucasian	CCS	56	40	96	ERCC5	rs17655 (G>C)
138	19318434	McKean C R, 2009	Brain	MassARRAY	USA	Caucasian	PBCCS	1004	1957	2961	ERCC5	rs17655 (G>C)
139	19280628	El Zein,R, 2009	Blood	TaqMan	USA	Mixed	CCS	198	219	417	ERCC5	rs17655 (G>C)
140	19096231	He,X 2008	Uterus	TaqMan	China	Asian	CCS	200	200	400	ERCC5	rs17655 (G>C)
141	17494052	Michiels,S, 2007	Lung	TaqMan	France	Caucasian	CCS	147	166	313	ERCC5	rs732321 (A>C)
								146	172	318	ERCC5	rs2018836 (A>G)
								143	172	315	ERCC5	rs3759500 (C>T)
								143	172	315	ERCC5	rs3818356 (G>A)
								146	172	318	ERCC5	rs4771436 (T>G)
142	17164380	Li,C, 2006	Skin	TaqMan	USA	Caucasian	HBCCS	602	603	1205	ERCC5	rs17655 (G>C)
143	16738949	Shen,M, 2006	Blood	TaqMan	USA	Caucasian	PBCCS	464	550	1014	ERCC5	rs17655 (G>C)
144	15494739	Blankenburg,S, 2005	Skin	PCR-RFLP	Germany	Caucasian	HBCCS	293	374	667	ERCC5	rs17655 (G>C)
145	14688016	Sanyal,S, 2004	Bladder	PCR-RFLP	Sweden	Caucasian	PBCCS	299	284	583	ERCC5	rs17655 (G>C)
146	12869423	Jeon,H.S, 2003	Lung	PCR-RFLP	Korea	Asian	HBCCS	310	311	621	ERCC5	rs17655 (G>C)
147	12494477	Kumar,R, 2003	Breast	PCR-RFLP	Finland	Caucasian	CCS	220	308	528	ERCC5	rs17655 (G>C)
148	25332048	Du,H, 2014	Colorectum	TaqMan	China	Asian	HBCCS	878	884	1762	ERCC5	rs17655 (G>C)
149	22969958	Yuan H, 2012	Head and Neck	TaqMan	China	Asian	HBCCS	394	884	1278	ERCC5	rs17655 (G>C)
150	21826087	P Biason, 2012	Bone	TaqMan	Italy	Caucasian	HBCCS	130	250	380	ERCC5	rs17655 (G>C)
151	20601096	Figl A, 2010	Skin	MassARRAY	Europe	Caucasian	PBCCS	1186	1274	2460	ERCC5	rs17655 (G>C)
152	17991492	B Pardini, 2008	Colorectum	PCR-RFLP	Czech	Caucasian	HBCCS	532	532	1064	ERCC5	rs17655 (G>C)
153	17975167	Wang LE, 2007	Skin	PCR-RFLP	USA	Caucasian	HBCCS	246	329	575	ERCC5	rs17655 (G>C)
154	17684138	An J, 2007	Head and Neck	PCR-RFLP	USA	Caucasian	HBCCS	829	854	1683	ERCC5	rs17655 (G>C)
155	16985021	Shen J, 2006	Breast	TaqMan	USA	Caucasian	CCS	154	151	305	ERCC5	rs17655 (G>C)
156	16646069	Valérie Le M, 2006	Soft tissue	PCR-RFLP	France	Caucasian	CCS	93	53	146	ERCC5	rs17655 (G>C)

157	16501254	Thirumaran RK, 2006	Skin	TaqMan	Europe	Caucasian	HBCCS	529	533	1062	ERCC5	rs17655 (G>C)
158	16094634	Cui Y, 2006	Lung	PCR-RFLP	USA	Mixed	CCS	497	902	1399	ERCC5	rs17655 (G>C)
159	15609317	Sakiyama T 2005	Lung	Pyrosequencing	Japan	Asian	CCS	1002	685	1687	ERCC5	rs17655 (G>C)
160		Bai Y, 2016	Stomach	PCR-RFLP	China	Asian	HBCCS	194	225	419	ERCC5	rs17655 (G>C)
								194	225	419	ERCC5	rs1800975 (T>C)

Abbreviations: OR, odds ratio; A, adenine; C, cytosine; G, guanine; T, thymine; HBCCS: Hospital-based case control study; CCS: Case-control study; PBCCS: Population-based case control study; ERCC: excision repair cross-complementation; HMLDTMS: high-throughput matrix-assisted laser desorption/ionization time-of-flight mass spectrometry; APEX: arrayed primer extension; DASH: dynamic allele-specific hybridization; KASPCR: kompetitive allele specific PCR; PCR-LDR: polymerase chain reaction/ligase detection reaction; PCR-RFLP: Polymerase Chain Reaction restriction fragment length polymorphism; ERCC: excision repair cross-complementation; RT-PCR: Real time Polymerase Chain Reaction restriction.

Reference: [1-160]

1. Zhang, Y., et al., Association between nucleotide excision repair gene polymorphism and colorectal cancer risk. Journal of clinical laboratory analysis, 2019. **33**(8): p. e22956.
2. Zhu, J., et al., Association between NER Pathway Gene Polymorphisms and Wilms Tumor Risk. Molecular therapy. Nucleic acids, 2018. **12**: p. 854-860.
3. Lawania, S., et al., XPF polymorphism toward lung cancer susceptibility and survival in patients treated with platinum-based chemotherapy. Future oncology (London, England), 2018. **14**(11): p. 1071-1089.
4. He, J., et al., Genetic variants in the nucleotide excision repair pathway genes and gastric cancer susceptibility in a southern Chinese population. Cancer management and research, 2018. **10**: p. 765-774.
5. Zhao, Z., et al., The association of polymorphisms in nucleotide excision repair genes with ovarian cancer susceptibility. Bioscience reports, 2018. **38**(3).
6. Zhuo, Z.-J., et al., Functional Polymorphisms at ERCC1/XPF Genes Confer Neuroblastoma Risk in Chinese Children. EBioMedicine, 2018. **30**: p. 113-119.
7. Carrera-Lasfuentes, P., et al., Relevance of DNA repair gene polymorphisms to gastric cancer risk and phenotype. Oncotarget, 2017. **8**(22): p. 35848-35862.
8. Dziki, L., et al., Modulation of Colorectal Cancer Risk by Polymorphisms in 51Gln/His, 64Ile/Val, and 148Asp/Glu of APEX Gene; 23Gly/Ala of XPA Gene; and 689Ser/Arg of ERCC4 Gene. Gastroenterology research and practice, 2017. **2017**: p. 3840243.
9. Wang, M., et al., Polymorphisms in nucleotide excision repair genes and risk of primary prostate cancer in Chinese Han populations. Oncotarget, 2017. **8**(15): p. 24362-24371.
10. He, B.-S., et al., Nucleotide excision repair pathway gene polymorphisms are linked to breast cancer risk in a Chinese population. Oncotarget, 2016. **7**(51): p. 84872-84882.
11. Ying, M.F. and R. Zhao, Role of single nucleotide polymorphisms of DNA repair genes in susceptibility to pancreatic cancer in Chinese population. Genetics and molecular research : GMR, 2016. **15**(1).
12. Sun, Y., et al., Association of NER pathway gene polymorphisms with susceptibility to laryngeal cancer in a Chinese population. International journal of clinical and experimental pathology, 2015. **8**(9): p. 11615-11621.
13. Zhao, F., et al., Association of single nucleotide polymorphisms of DNA repair genes in NER pathway and susceptibility to pancreatic cancer. International journal of clinical and experimental pathology, 2015. **8**(9): p. 11579-11586.

14. Kabziński, J., et al., The Role of the XPF Gene Polymorphism (Xrcc4) Ser835ser in the Risk of Malignant Transformation of Cells in the Colorectal Cancer. Polski przeglad chirurgiczny, 2015. **87**(2): p. 83-85.
15. Yang, H., G. Li, and W.F. Li, Association between ERCC1 and XPF polymorphisms and risk of colorectal cancer. Genetics and molecular research : GMR, 2015. **14**(1): p. 700-705.
16. Paszkowska-Szczur, K., et al., Polymorphisms in nucleotide excision repair genes and susceptibility to colorectal cancer in the Polish population. Molecular biology reports, 2015. **42**(3): p. 755-764.
17. Pesz, K.A., et al., Polymorphisms in nucleotide excision repair genes and basal cell carcinoma of the skin. International journal of dermatology, 2014. **53**(12): p. 1474-1477.
18. Zhou, W.K., et al., Association of polymorphisms of the xeroderma pigmentosum complementation group F gene with increased glioma risk. Genetics and molecular research : GMR, 2014. **13**(2): p. 3826-3831.
19. Mirecka, A., et al., Common variants of xeroderma pigmentosum genes and prostate cancer risk. Gene, 2014. **546**(2): p. 156-161.
20. Hou, R., et al., Association of single nucleotide polymorphisms of ERCC1 and XPF with colorectal cancer risk and interaction with tobacco use. Gene, 2014. **548**(1): p. 1-5.
21. Pei, X.H., et al., Genetic variation in ERCC1 and XPF genes and breast cancer risk. Genetics and molecular research : GMR, 2014. **13**(1): p. 2259-2267.
22. Liu, Y., et al., XPF-673C>T polymorphism effect on the susceptibility to esophageal cancer in Chinese population. PloS one, 2014. **9**(4): p. e94136.
23. Steck, S.E., et al., Nucleotide excision repair gene polymorphisms, meat intake and colon cancer risk. Mutation research, 2014. **762**: p. 24-31.
24. Lu, B., et al., Laryngeal cancer risk and common single nucleotide polymorphisms in nucleotide excision repair pathway genes ERCC1, ERCC2, ERCC3, ERCC4, ERCC5 and XPA. Gene, 2014. **542**(1): p. 64-68.
25. Li, X., et al., Association of single nucleotide polymorphisms of nucleotide excision repair genes with laryngeal cancer risk and interaction with cigarette smoking and alcohol drinking. Tumour biology : the journal of the International Society for Oncodevelopmental Biology and Medicine, 2014. **35**(5): p. 4659-4665.
26. Kohlhase, S., et al., Mutation analysis of the ERCC4/FANCQ gene in hereditary breast cancer. PloS one, 2014. **9**(1): p. e85334.
27. Cheng, H.-B., et al., Xeroderma pigmentosum complementation group f polymorphisms influence risk of glioma. Asian Pacific journal of cancer prevention : APJCP, 2013. **14**(7): p. 4083-4087.
28. Santos, L.S., et al., The role of CCNH Val270Ala (rs2230641) and other nucleotide excision repair polymorphisms in individual susceptibility to well-differentiated thyroid cancer. Oncology reports, 2013. **30**(5): p. 2458-2466.
29. Wang, X.-F., S. Liu, and Z.-K. Shao, Effects of polymorphisms in nucleotide excision repair genes on glioma risk in a Chinese population. Gene, 2013. **529**(2): p. 317-320.
30. Yang, Z., et al., Polymorphisms in the ERCC1 and XPF genes and risk of breast cancer in a Chinese population. Genetic testing and molecular biomarkers, 2013. **17**(9): p. 700-706.
31. Wyss, A.B., et al., Single-nucleotide polymorphisms in nucleotide excision repair genes, cigarette smoking, and the risk of head and neck cancer. Cancer epidemiology, biomarkers & prevention : a publication of the American Association for Cancer Research, cosponsored by the American Society of Preventive Oncology, 2013. **22**(8): p. 1428-1445.
32. Zhang, J.-S., et al., Effect of Xeroderma pigmentosum complementation group F polymorphisms on gastric cancer risk and associations with H.pylori infection. Asian Pacific journal of cancer prevention : APJCP, 2013. **14**(3): p. 1847-1850.
33. Oliveira, C., et al., Assessment of the XPC (A2920C), XPF (T30028C), TP53 (Arg72Pro) and GSTP1 (Ile105Val) polymorphisms in the risk of cutaneous melanoma. Journal of cancer research and clinical oncology, 2013. **139**(7): p. 1199-1206.

34. Chu, H., et al., Tagging SNPs in the ERCC4 gene are associated with gastric cancer risk. Gene, 2013. **521**(1): p. 50-54.
35. Paszkowska-Szczur, K., et al., Xeroderma pigmentosum genes and melanoma risk. International journal of cancer, 2013. **133**(5): p. 1094-1100.
36. He, J., et al., Polymorphisms in ERCC1 and XPF genes and risk of gastric cancer in an eastern Chinese population. PloS one, 2012. **7**(11): p. e49308.
37. Yu, H., et al., Association between single nucleotide polymorphisms in ERCC4 and risk of squamous cell carcinoma of the head and neck. PloS one, 2012. **7**(7): p. e41853.
38. Sakoda, L.C., et al., Germ line variation in nucleotide excision repair genes and lung cancer risk in smokers. International journal of molecular epidemiology and genetics, 2012. **3**(1).
39. Zhou, R.-M., et al., ERCC1 gene +262A/C polymorphism associated with risk of gastric cardiac adenocarcinoma in nonsmokers. Archives of medical research, 2012. **43**(1): p. 67-74.
40. Doherty, J.A., et al., Polymorphisms in nucleotide excision repair genes and endometrial cancer risk. Cancer epidemiology, biomarkers & prevention : a publication of the American Association for Cancer Research, cosponsored by the American Society of Preventive Oncology, 2011. **20**(9): p. 1873-1882.
41. Roberts, M.R., et al., Single-nucleotide polymorphisms in DNA repair genes and association with breast cancer risk in the web study. Carcinogenesis, 2011. **32**(8): p. 1223-1230.
42. Gil, J., et al., The C/A polymorphism in intron 11 of the XPC gene plays a crucial role in the modulation of an individual's susceptibility to sporadic colorectal cancer. Molecular biology reports, 2012. **39**(1): p. 527-534.
43. Ming-Shiean, H., et al., Synergistic effects of polymorphisms in DNA repair genes and endogenous estrogen exposure on female breast cancer risk. Annals of surgical oncology, 2010. **17**(3): p. 760-771.
44. Rajaraman, P., et al., DNA repair gene polymorphisms and risk of adult meningioma, glioma, and acoustic neuroma. Neuro-oncology, 2010. **12**(1): p. 37-48.
45. Wang, M., et al., A novel XPF -357A>C polymorphism predicts risk and recurrence of bladder cancer. Oncogene, 2010. **29**(13): p. 1920-1928.
46. Agalliu, I., et al., Genetic variation in DNA repair genes and prostate cancer risk: results from a population-based study. Cancer causes & control : CCC, 2010. **21**(2): p. 289-300.
47. Abbasi, R., et al., Laryngeal cancer risk associated with smoking and alcohol consumption is modified by genetic polymorphisms in ERCC5, ERCC6 and RAD23B but not by polymorphisms in five other nucleotide excision repair genes. International journal of cancer, 2009. **125**(6): p. 1431-1439.
48. Pan, J., et al., Genetic susceptibility to esophageal cancer: the role of the nucleotide excision repair pathway. Carcinogenesis, 2009. **30**(5): p. 785-792.
49. Joshi, A.D., et al., Red meat and poultry intake, polymorphisms in the nucleotide excision repair and mismatch repair pathways and colorectal cancer risk. Carcinogenesis, 2009. **30**(3): p. 472-479.
50. Rajaraman, P., et al., Nucleotide excision repair polymorphisms may modify ionizing radiation-related breast cancer risk in US radiologic technologists. International journal of cancer, 2008. **123**(11): p. 2713-2716.
51. Lin, J., et al., Case-control analysis of nucleotide excision repair pathway and the risk of renal cell carcinoma. Carcinogenesis, 2008. **29**(11): p. 2112-2119.
52. Chang, J.S., et al., Nucleotide excision repair genes and risk of lung cancer among San Francisco Bay Area Latinos and African Americans. International journal of cancer, 2008. **123**(9): p. 2095-2104.
53. Han, J., et al., Genetic variation in DNA repair pathway genes and premenopausal breast cancer risk. Breast cancer research and treatment, 2009. **115**(3): p. 613-622.
54. McWilliams, R.R., et al., Polymorphisms in DNA repair genes, smoking, and pancreatic adenocarcinoma risk. Cancer research, 2008. **68**(12): p. 4928-4935.
55. Shao, M., et al., Polymorphisms in excision repair cross-complementing group 4 (ERCC4) and susceptibility to primary lung cancer in a Chinese Han population. Lung cancer (Amsterdam, Netherlands), 2008. **60**(3): p. 332-339.

56. Hooker, S., et al., NAT2 and NER genetic variants and sporadic prostate cancer susceptibility in African Americans. Prostate cancer and prostatic diseases, 2008. **11**(4): p. 349-356.
57. Crew, K.D., et al., Polymorphisms in nucleotide excision repair genes, polycyclic aromatic hydrocarbon-DNA adducts, and breast cancer risk. Cancer epidemiology, biomarkers & prevention : a publication of the American Association for Cancer Research, cosponsored by the American Society of Preventive Oncology, 2007. **16**(10): p. 2033-2041.
58. Chen, M., et al., High-order interactions among genetic polymorphisms in nucleotide excision repair pathway genes and smoking in modulating bladder cancer risk. Carcinogenesis, 2007. **28**(10): p. 2160-2165.
59. Povey, J.E., et al., DNA repair gene polymorphisms and genetic predisposition to cutaneous melanoma. Carcinogenesis, 2007. **28**(5): p. 1087-1093.
60. Jorgensen, T.J., et al., Breast cancer risk is not associated with polymorphic forms of xeroderma pigmentosum genes in a cohort of women from Washington County, Maryland. Breast cancer research and treatment, 2007. **101**(1): p. 65-71.
61. Moreno, V., et al., Polymorphisms in genes of nucleotide and base excision repair: risk and prognosis of colorectal cancer. Clinical cancer research : an official journal of the American Association for Cancer Research, 2006. **12**(7 Pt 1): p. 2101-2108.
62. García-Closas, M., et al., Genetic variation in the nucleotide excision repair pathway and bladder cancer risk. Cancer epidemiology, biomarkers & prevention : a publication of the American Association for Cancer Research, cosponsored by the American Society of Preventive Oncology, 2006. **15**(3): p. 536-542.
63. Huang, W.-Y., et al., Nucleotide excision repair gene polymorphisms and risk of advanced colorectal adenoma: XPC polymorphisms modify smoking-related risk. Cancer epidemiology, biomarkers & prevention : a publication of the American Association for Cancer Research, cosponsored by the American Society of Preventive Oncology, 2006. **15**(2): p. 306-311.
64. Wu, X., et al., Bladder cancer predisposition: a multigenic approach to DNA-repair and cell-cycle-control genes. American journal of human genetics, 2006. **78**(3): p. 464-479.
65. Sugimura, T., et al., Gene-environment interaction involved in oral carcinogenesis: molecular epidemiological study for metabolic and DNA repair gene polymorphisms. Journal of oral pathology & medicine : official publication of the International Association of Oral Pathologists and the American Academy of Oral Pathology, 2006. **35**(1): p. 11-18.
66. Matullo, G., et al., Polymorphisms/haplotypes in DNA repair genes and smoking: a bladder cancer case-control study. Cancer epidemiology, biomarkers & prevention : a publication of the American Association for Cancer Research, cosponsored by the American Society of Preventive Oncology, 2005. **14**(11 Pt 1): p. 2569-2578.
67. Weiss, J.M., et al., Interindividual variation in nucleotide excision repair genes and risk of endometrial cancer. Cancer epidemiology, biomarkers & prevention : a publication of the American Association for Cancer Research, cosponsored by the American Society of Preventive Oncology, 2005. **14**(11 Pt 1): p. 2524-2530.
68. Zienolddiny, S., et al., Polymorphisms of DNA repair genes and risk of non-small cell lung cancer. Carcinogenesis, 2006. **27**(3): p. 560-567.
69. Lee, S.-A., et al., Obesity and genetic polymorphism of ERCC2 and ERCC4 as modifiers of risk of breast cancer. Experimental & molecular medicine, 2005. **37**(2): p. 86-90.
70. Smith, T.R., et al., DNA-repair genetic polymorphisms and breast cancer risk. Cancer epidemiology, biomarkers & prevention : a publication of the American Association for Cancer Research, cosponsored by the American Society of Preventive Oncology, 2003. **12**(11 Pt 1): p. 1200-1204.
71. Torres, S.M., et al., DNA repair variants, indoor tanning, and risk of melanoma. Pigment cell & melanoma research, 2013. **26**(5): p. 677-684.
72. Gong, Y., et al., Association of two ERCC4 tagSNPs with susceptibility to atrophic gastritis and gastric cancer in Chinese. Gene, 2013. **519**(2): p. 335-342.
73. Gaudet, M.M., et al., Five polymorphisms and breast cancer risk: results from the Breast Cancer Association Consortium. Cancer epidemiology, biomarkers & prevention : a publication of the American Association for Cancer Research, cosponsored by the American Society of Preventive Oncology, 2009. **18**(5): p. 1610-1616.
74. Gao, K., S.Q. Mu, and Z.X. Wu, Investigation of the effects of single-nucleotide polymorphisms in DNA repair genes on the risk of glioma. Genetics and molecular research :

GMR, 2014. **13**(1): p. 1203-1211.
75. Ibarrola-Villava, M., et al., Genetic polymorphisms in DNA repair and oxidative stress pathways associated with malignant melanoma susceptibility. European journal of cancer (Oxford, England : 1990), 2011. **47**(17): p. 2618-2625.
76. Smith, T.R., et al., Polygenic model of DNA repair genetic polymorphisms in human breast cancer risk. Carcinogenesis, 2008. **29**(11): p. 2132-2138.
77. Mechanic, L.E., et al., Polymorphisms in nucleotide excision repair genes, smoking and breast cancer in African Americans and whites: a population-based case-control study. Carcinogenesis, 2006. **27**(7): p. 1377-1385.
78. Shen, M., et al., Polymorphisms in the DNA nucleotide excision repair genes and lung cancer risk in Xuan Wei, China. International journal of cancer, 2005. **116**(5): p. 768-773.
79. Winsey, S.L., et al., A variant within the DNA repair gene XRCC3 is associated with the development of melanoma skin cancer. Cancer research, 2000. **60**(20): p. 5612-5616.
80. Wang, B., et al., The association of six polymorphisms of five genes involved in three steps of nucleotide excision repair pathways with hepatocellular cancer risk. Oncotarget, 2016. **7**(15): p. 20357-20367.
81. Hill, D.A., et al., Risk of non-Hodgkin lymphoma (NHL) in relation to germline variation in DNA repair and related genes. Blood, 2006. **108**(9): p. 3161-3167.
82. Osorio, A., et al., Evaluation of a candidate breast cancer associated SNP in ERCC4 as a risk modifier in BRCA1 and BRCA2 mutation carriers. Results from the Consortium of Investigators of Modifiers of BRCA1/BRCA2 (CIMBA). British journal of cancer, 2009. **101**(12): p. 2048-2054.
83. Mordukhovich, I., et al., Polymorphisms in DNA repair genes, traffic-related polycyclic aromatic hydrocarbon exposure and breast cancer incidence. International journal of cancer, 2016. **139**(2): p. 310-321.
84. Luo, K.-Q., et al., Polymorphisms in DNA repair genes and risk of glioma and meningioma. Asian Pacific journal of cancer prevention : APJCP, 2013. **14**(1): p. 449-452.
85. Li, Y.-K., et al., Nucleotide excision repair pathway gene polymorphisms are associated with risk and prognosis of colorectal cancer. World journal of gastroenterology, 2020. **26**(3): p. 307-323.
86. Minina, V.I., et al., Polymorphisms in DNA repair genes in lung cancer patients living in a coal-mining region. European journal of cancer prevention : the official journal of the European Cancer Prevention Organisation (ECP), 2019. **28**(6): p. 522-528.
87. Shakil Malik, S., et al., Genetic polymorphism in and breast cancer risk. Molecular biology research communications, 2019. **8**(1): p. 27-31.
88. Nigam, K., et al., Risk Modulation of Oral Pre Cancer and Cancer with Polymorphisms in XPD and XPG Genes in North Indian Population. Asian Pacific journal of cancer prevention : APJCP, 2019. **20**(8): p. 2397-2403.
89. Santos, L.S., et al., Thyroid Cancer: The Quest for Genetic Susceptibility Involving DNA Repair Genes. Genes, 2019. **10**(8).
90. Su, J., et al., Asp1104His polymorphism increases colorectal cancer risk especially in Asians. American journal of translational research, 2019. **11**(2): p. 1020-1029.
91. Lawania, S., et al., XPG polymorphisms and their association with lung cancer susceptibility, overall survival and response in North Indian patients treated with platinum-based doublet chemotherapy. Future oncology (London, England), 2019. **15**(2): p. 151-165.
92. Pongsavee, M. and K. Wisuwan, rs751402 polymorphism is the risk factor for sporadic breast cancer in Thailand. International journal of molecular epidemiology and genetics, 2018. **9**(4): p. 27-33.
93. Zhang, Z., et al., Association between the XPG gene rs2094258 polymorphism and risk of gastric cancer. Journal of clinical laboratory analysis, 2018. **32**(8): p. e22564.
94. Sang, L., et al., Impact of SNP-SNP interactions of DNA repair gene and metabolic gene on gastric cancer/atrophic gastritis risk in a Chinese population. World journal of gastroenterology, 2018. **24**(5): p. 602-612.

95. Pérez-Ramírez, C., et al., Impact of DNA repair, folate and glutathione gene polymorphisms on risk of non small cell lung cancer. Pathology, research and practice, 2018. **214**(1): p. 44-52.
96. Zhang, C., et al., Study on association between ERCC5 single nucleotide polymorphism and susceptibility to esophageal cancer. Journal of B.U.ON. : official journal of the Balkan Union of Oncology, 2017. **22**(4): p. 979-984.
97. Li, X., et al., The association between polymorphisms in the DNA nucleotide excision repair genes and RRM1 gene and lung cancer risk. Thoracic cancer, 2012. **3**(3): p. 239-248.
98. Lonjou, C., et al., Investigation of DNA repair-related SNPs underlying susceptibility to papillary thyroid carcinoma reveals MGMT as a novel candidate gene in Belarusian children exposed to radiation. BMC cancer, 2017. **17**(1): p. 328.
99. Hua, R.-X., et al., Association between genetic variants in the gene and gastric cancer risk in a Southern Chinese population. Aging, 2016. **8**(12): p. 3311-3320.
100. Yang, L.Q., Y. Zhang, and H.F. Sun, Investigation on ERCC5 genetic polymorphisms and the development of gastric cancer in a Chinese population. Genetics and molecular research : GMR, 2016. **15**(3).
101. Hua, R.-X., et al., Gene Polymorphisms Contribute to Colorectal Cancer Susceptibility: A Two-Stage Case-Control Study. Journal of Cancer, 2016. **7**(12): p. 1731-1739.
102. Guo, B.W., et al., Association between ERCC5 gene polymorphisms and gastric cancer risk. Genetics and molecular research : GMR, 2016. **15**(2).
103. Feng, Y.B., et al., Association between XPG gene polymorphisms and development of gastric cancer risk in a Chinese population. Genetics and molecular research : GMR, 2016. **15**(2).
104. Lu, J.J., et al., Lack of association between ERCC5 gene polymorphisms and gastric cancer risk in a Chinese population. Genetics and molecular research : GMR, 2016. **15**(2).
105. Ma, S.H., et al., Investigation on the role of XPG gene polymorphisms in breast cancer risk in a Chinese population. Genetics and molecular research : GMR, 2016. **15**(2).
106. Zhou, R.-M., et al., XPG Gene Polymorphisms and the Risk of Gastric Cardia Adenocarcinoma. Genetic testing and molecular biomarkers, 2016. **20**(8): p. 432-437.
107. Li, R.J., et al., Association between ERCC5 gene polymorphisms and gastric cancer risk in a Chinese population. Genetics and molecular research : GMR, 2016. **15**(1).
108. He, J., et al., Association of potentially functional variants in the XPG gene with neuroblastoma risk in a Chinese population. Journal of cellular and molecular medicine, 2016. **20**(8): p. 1481-1490.
109. Chen, Y.-Z., et al., Association between XPG polymorphisms and stomach cancer susceptibility in a Chinese population. Journal of cellular and molecular medicine, 2016. **20**(5): p. 903-908.
110. Joo, J., et al., Nucleotide Excision Repair Gene ERCC2 and ERCC5 Variants Increase Risk of Uterine Cervical Cancer. Cancer research and treatment : official journal of Korean Cancer Association, 2016. **48**(2): p. 708-714.
111. Na, N., et al., Association between ERCC5 gene polymorphisms and breast cancer risk. International journal of clinical and experimental pathology, 2015. **8**(3): p. 3192-3197.
112. Kabzinski, J., et al., An association of selected ERCC2 and ERCC5 genes polymorphisms, the level of oxidative DNA damage and its repair efficiency with a risk of colorectal cancer in Polish population. Cancer biomarkers : section A of Disease markers, 2015. **15**(4): p. 413-423.
113. Wang, H., et al., Association analysis of ERCC5 gene polymorphisms with risk of breast cancer in Han women of northwest China. Breast cancer (Tokyo, Japan), 2016. **23**(3): p. 479-485.
114. Bahceci, A., et al., DNA repair gene polymorphisms in B cell non-Hodgkin's lymphoma. Tumour biology : the journal of the International Society for Oncodevelopmental Biology and Medicine, 2015. **36**(3): p. 2155-2161.
115. Sun, K., A. Gong, and P. Liang, Predictive impact of genetic polymorphisms in DNA repair genes on susceptibility and therapeutic outcomes to colorectal cancer patients. Tumour

biology : the journal of the International Society for Oncodevelopmental Biology and Medicine, 2015. **36**(3): p. 1549-1559.
116. Douzi, K., et al., Polymorphisms in XPC, XPD and XPG DNA repair genes and leukemia risk in a Tunisian population. Leukemia & lymphoma, 2015. **56**(6): p. 1856-1862.
117. Fabisiewicz, A., et al., Polymorphisms of DNA repair and oxidative stress genes in B-cell lymphoma patients. Biomedical reports, 2013. **1**(1): p. 151-155.
118. Zhang, X.J., P. Liu, and F. Zhu, Polymorphisms of DNA repair-related genes with susceptibility and prognosis of prostate cancer. Genetics and molecular research : GMR, 2014. **13**(2): p. 4419-4424.
119. Yang, B., et al., Role of DNA repair-related gene polymorphisms in susceptibility to risk of prostate cancer. Asian Pacific journal of cancer prevention : APJCP, 2013. **14**(10): p. 5839-5842.
120. Ruiz-Cosano, J., D. Torres-Moreno, and P. Conesa-Zamora, Influence of polymorphisms in ERCC5, XPA and MTR DNA repair and synthesis genes in B-cell lymphoma risk. A case-control study in Spanish population. Journal of B.U.ON. : official journal of the Balkan Union of Oncology, 2013. **18**(2): p. 486-490.
121. Yang, W.-G., et al., SNPs of excision repair cross complementing group 5 and gastric cancer risk in Chinese populations. Asian Pacific journal of cancer prevention : APJCP, 2012. **13**(12): p. 6269-6272.
122. Wen, H., et al., Study on bladder cancer susceptibility and genetic polymorphisms of XPC, XPG, and CYP in smokers and non-smokers. Actas urologicas espanolas, 2013. **37**(5): p. 259-265.
123. Duan, Z., et al., Promoter polymorphisms in DNA repair gene ERCC5 and susceptibility to gastric cancer in Chinese. Gene, 2012. **511**(2): p. 274-279.
124. Zavras, A.I., et al., Association between polymorphisms of DNA repair gene ERCC5 and oral squamous cell carcinoma. Oral surgery, oral medicine, oral pathology and oral radiology, 2012. **114**(5): p. 624-629.
125. Yoon, A.J., et al., Role of ERCC5 polymorphism in risk of hepatocellular carcinoma. Oncology letters, 2011. **2**(5): p. 911-914.
126. Zhu, M.-L., et al., Polymorphisms in the ERCC5 gene and risk of esophageal squamous cell carcinoma (ESCC) in Eastern Chinese populations. PloS one, 2012. **7**(7): p. e41500.
127. He, J., et al., Polymorphisms in the XPG gene and risk of gastric cancer in Chinese populations. Human genetics, 2012. **131**(7): p. 1235-1244.
128. Cincin, Z.B., et al., DNA repair gene variants in endometrial carcinoma. Medical oncology (Northwood, London, England), 2012. **29**(4): p. 2949-2954.
129. Liu, D., et al., DNA repair genes XPC, XPG polymorphisms: relation to the risk of colorectal carcinoma and therapeutic outcome with Oxaliplatin-based adjuvant chemotherapy. Molecular carcinogenesis, 2012. **51 Suppl 1**: p. E83-E93.
130. Ma, H., et al., Polymorphisms of XPG/ERCC5 and risk of squamous cell carcinoma of the head and neck. Pharmacogenetics and genomics, 2012. **22**(1): p. 50-57.
131. Gonçalves, F.T., et al., European ancestry and polymorphisms in DNA repair genes modify the risk of melanoma: a case-control study in a high UV index region in Brazil. Journal of dermatological science, 2011. **64**(1): p. 59-66.
132. Berhane, N., R.C. Sobti, and S.A. Mahdi, DNA repair genes polymorphism (XPG and XRCC1) and association of prostate cancer in a north Indian population. Molecular biology reports, 2012. **39**(3): p. 2471-2479.
133. Rouissi, K., et al., Smoking and polymorphisms in xenobiotic metabolism and DNA repair genes are additive risk factors affecting bladder cancer in Northern Tunisia. Pathology oncology research : POR, 2011. **17**(4): p. 879-886.
134. Canbay, E., et al., Association of APE1 and hOGG1 polymorphisms with colorectal cancer risk in a Turkish population. Current medical research and opinion, 2011. **27**(7): p. 1295-1302.
135. Rouissi, K., et al., The effect of tobacco, XPC, ERCC2 and ERCC5 genetic variants in bladder cancer development. BMC cancer, 2011. **11**: p. 101.

136. Canbay, E., et al., Possible associations of APE1 polymorphism with susceptibility and HOGG1 polymorphism with prognosis in gastric cancer. Anticancer research, 2010. **30**(4): p. 1359-1364.

137. Narter, K.F., et al., Bladder cancer and polymorphisms of DNA repair genes (XRCC1, XRCC3, XPD, XPG, APE1, hOGG1). Anticancer research, 2009. **29**(4): p. 1389-1393.

138. McKean-Cowdin, R., et al., Associations between polymorphisms in DNA repair genes and glioblastoma. Cancer epidemiology, biomarkers & prevention : a publication of the American Association for Cancer Research, cosponsored by the American Society of Preventive Oncology, 2009. **18**(4): p. 1118-1126.

139. El-Zein, R., et al., Genetic polymorphisms in DNA repair genes as modulators of Hodgkin disease risk. Cancer, 2009. **115**(8): p. 1651-1659.

140. He, X., et al., Susceptibility of XRCC3, XPD, and XPG genetic variants to cervical carcinoma. Pathobiology : journal of immunopathology, molecular and cellular biology, 2008. **75**(6): p. 356-363.

141. Michiels, S., et al., Polymorphism discovery in 62 DNA repair genes and haplotype associations with risks for lung and head and neck cancers. Carcinogenesis, 2007. **28**(8): p. 1731-1739.

142. Li, C., et al., Polymorphisms in the DNA repair genes XPC, XPD, and XPG and risk of cutaneous melanoma: a case-control analysis. Cancer epidemiology, biomarkers & prevention : a publication of the American Association for Cancer Research, cosponsored by the American Society of Preventive Oncology, 2006. **15**(12): p. 2526-2532.

143. Shen, M., et al., Polymorphisms in DNA repair genes and risk of non-Hodgkin lymphoma among women in Connecticut. Human genetics, 2006. **119**(6): p. 659-668.

144. Blankenburg, S., et al., No association between three xeroderma pigmentosum group C and one group G gene polymorphisms and risk of cutaneous melanoma. European journal of human genetics : EJHG, 2005. **13**(2): p. 253-255.

145. Sanyal, S., et al., Polymorphisms in DNA repair and metabolic genes in bladder cancer. Carcinogenesis, 2004. **25**(5): p. 729-734.

146. Jeon, H.-S., et al., Relationship between XPG codon 1104 polymorphism and risk of primary lung cancer. Carcinogenesis, 2003. **24**(10): p. 1677-1681.

147. Kumar, R., et al., Single nucleotide polymorphisms in the XPG gene: determination of role in DNA repair and breast cancer risk. International journal of cancer, 2003. **103**(5): p. 671-675.

148. Du, H., et al., Association study between XPG Asp1104His polymorphism and colorectal cancer risk in a Chinese population. Scientific reports, 2014. **4**: p. 6700.

149. Yuan, H., et al., Genetic polymorphisms in key DNA repair genes and risk of head and neck cancer in a Chinese population. Experimental and therapeutic medicine, 2012. **3**(4): p. 719-724.

150. Biason, P., et al., Nucleotide excision repair gene variants and association with survival in osteosarcoma patients treated with neoadjuvant chemotherapy. The pharmacogenomics journal, 2012. **12**(6): p. 476-483.

151. Figl, A., et al., Single-nucleotide polymorphisms in DNA-repair genes and cutaneous melanoma. Mutation research, 2010. **702**(1).

152. Pardini, B., et al., DNA repair genetic polymorphisms and risk of colorectal cancer in the Czech Republic. Mutation research, 2008. **638**(1-2): p. 146-153.

153. Wang, L.-E., et al., Repair capacity for UV light induced DNA damage associated with risk of nonmelanoma skin cancer and tumor progression. Clinical cancer research : an official journal of the American Association for Cancer Research, 2007. **13**(21): p. 6532-6539.

154. An, J., et al., Potentially functional single nucleotide polymorphisms in the core nucleotide excision repair genes and risk of squamous cell carcinoma of the head and neck. Cancer epidemiology, biomarkers & prevention : a publication of the American Association for Cancer Research, cosponsored by the American Society of Preventive Oncology, 2007. **16**(8): p. 1633-1638.

155. Shen, J., et al., Polymorphisms in nucleotide excision repair genes and DNA repair capacity phenotype in sisters discordant for breast cancer. Cancer epidemiology, biomarkers &

prevention : a publication of the American Association for Cancer Research, cosponsored by the American Society of Preventive Oncology, 2006. **15**(9): p. 1614-1619.
156. Le Morvan, V., et al., Genetic polymorphisms of the XPG and XPD nucleotide excision repair genes in sarcoma patients. International journal of cancer, 2006. **119**(7): p. 1732-1735.
157. Thirumaran, R.K., et al., Single nucleotide polymorphisms in DNA repair genes and basal cell carcinoma of skin. Carcinogenesis, 2006. **27**(8): p. 1676-1681.
158. Cui, Y., et al., Polymorphism of Xeroderma Pigmentosum group G and the risk of lung cancer and squamous cell carcinomas of the oropharynx, larynx and esophagus. International journal of cancer, 2006. **118**(3): p. 714-720.
159. Sakiyama, T., et al., Association of amino acid substitution polymorphisms in DNA repair genes TP53, POLI, REV1 and LIG4 with lung cancer risk. International journal of cancer, 2005. **114**(5): p. 730-737.
160. Yu Bai, H.L., Yan Li, Lei Wang, Xiangjie Fang, Role of ERCC5 His1104Asp and His46His gene polymorphisms in the development of gastric cancer risk in a Chinese Han population. Int J Clin Exp Pathol, 2016. **9**(3): p. 3925-3930.
