# Supplementary material for: Polymorphisms in ERCC4 and ERCC5 and risk of cancers: Systematic research synopsis, meta-analysis, and epidemiological evidence
Source: Front Oncol. 2022 Aug 11;12:951193. doi: 10.3389/fonc.2022.951193 (PMC9404303; doi:10.3389/fonc.2022.951193)
Supplement: Supplementary file 8 [file Table_4.pdf]

Supplementary Table S4. Associations between variants in the ERCC4 and ERCC5 with cancer risk.

ID	Gene	Variant	Alleles[a]	cancer site	Ethnicity	MAF[b]	Genetic Models	Effect model	Number Evaluation: studises	Number Evaluation: sample size (case/controls)	Risk of Meta-Analysis: OR (95%CI)	Risk of Meta-Analysis: P_{value}	Heterogeneity: I^2 (%)	Heterogeneity: P_Q	Egger	Begg	Venice Criteria[c]	Venice Criteria Grade	Amount of Evidence: N_{minor}	Amount of Evidence: Grade	Replication	Protection from Bias: Grade	Protection from Bias: Reasons for Bias	First Study	Reason for bias Exemption	Deviation from HWE	Pegger	Pbegger	Low OR	FPRP values[d]	Credibility of Evidence[e]
1	ERCC4	rs1799801	C vs T	Bladder	Overall	0.3009	Allelic	Fixed	3	3417 (1701/1716)	0.990 (0.887-1.106)	0.861	0.0	0.710	0.199	1.000															
							Dominant	Fixed	3	3417 (1701/1716)	1.042 (0.903-1.202)	0.575	0.0	0.525	0.029	0.296															
							Recessive	Fixed	3	3417 (1701/1716)	0.845 (0.662-1.080)	0.179	0.0	0.793	0.680	1.000															
					Asian	0.2480	Allelic	Fixed	1	484 (234/250)	1.034 (0.773-1.383)	0.822	NA	NA	NA	NA															
							Dominant	Fixed	1	484 (234/250)	1.163 (0.811-1.666)	0.412	NA	NA	NA	NA															
							Recessive	Fixed	1	484 (234/250)	0.676 (0.329-1.333)	0.289	NA	NA	NA	NA															
					Caucasian	0.3117	Allelic	Fixed	2	2527 (1295/1232)	0.983 (0.872-1.108)	0.778	0.0	0.444	NA	NA															
							Dominant	Fixed	2	2527 (1295/1232)	1.021 (0.873-1.193)	0.799	0.0	0.352	NA	NA															
							Recessive	Fixed	2	2527 (1295/1232)	0.871 (0.671-1.130)	0.299	0.0	0.826	NA	NA															
2	ERCC4	rs2020955	C vs T	Bladder	Caucasian	0.0048	Allelic	Fixed	3	4475 (2280/2195)	1.413 (0.811-2.462)	0.222	0.0	0.442	NA	0.602															
							Dominant	Fixed	3	4475 (2280/2195)	1.416 (0.811-2.472)	0.221	0.0	0.442	NA	0.602															
							Recessive	Fixed	3	4475 (2280/2195)	NA	NA	NA	NA	NA	NA															
3	ERCC4	rs744154	C vs G	Bladder	Asian	0.7213	Allelic	Fixed	2	484 (234/250)	1.566 (1.233-1.989)	0.000	0.0	0.789	NA	1.000	Strong	AAA	1161	A	A	A	No	NA	NA	NA	NA	1.000	No	0.012	Strong
							Dominant	Fixed	2	484 (234/250)	2.061 (0.962-4.417)	0.063	0.0	0.614	NA	1.000															
3	ERCC4	rs744154	C vs G	Bladder	Asian	0.7213	Recessive	Fixed	2	484 (234/250)	1.731 (1.296-2.313)	0.000	0.0	0.862	NA	1.000	Moderate	BAA	429	B	A	A	No	NA	NA	NA	NA	1.000	No	0.023	Strong
4	ERCC4	rs1799798	A vs G	Brain	Asian	0.8906	Allelic	Fixed	2	930 (432/498)	0.842 (0.635-1.117)	0.233	0.0	0.993	NA	1.000															
							Dominant	Fixed	2	930 (432/498)	0.491 (0.231-1.043)	0.064	0.0	0.942	NA	1.000															
							Recessive	Fixed	2	930 (432/498)	0.981 (0.667-1.264)	0.600	0.0	0.973	NA	1.000															
5	ERCC4	rs1800067	A vs G	Brain (Glioma)	Overall	0.7253	Allelic	Random	5	3502 (1481/2021)	0.634 (0.426-0.944)	0.025	82.9	0.000	0.372	0.806	Weak	ACC	1424	A	C	C	First study; HWE	Yes	No	Yes	0.372	0.806	No	0.540	Weak
							Dominant	Random	5	3502 (1481/2021)	0.680 (0.390-1.184)	0.172	57.5	0.052	0.104	1.000															
							Recessive	Random	5	3502 (1481/2021)	0.528 (0.350-0.796)	0.002	69.3	0.011	0.909	0.462	Weak	ACA	3421	A	C	A	No	No	No	No	0.909	0.462	No	0.247	Weak
					Asian	0.9263	Allelic	Random	4	2645 (1119/1526)	0.543 (0.389-0.760)	0.000	69.5	0.020	0.542	0.734	Weak	ACA	4707	A	C	A	No	No	No	No	0.542	0.734	No	0.057	Weak
							Dominant	Random	4	2645 (1119/1526)	0.502 (0.312-0.808)	0.005	0.0	0.825	0.442	0.308	Weak	AAC	2537	A	A	C	First study; HWE	Yes	No	Yes	0.442	0.308	No	0.416	Weak
							Recessive	Random	4	2645 (1119/1526)	0.535 (0.347-0.826)	0.005	76.7	0.005	0.143	0.089	Weak	ACC	2170	A	C	C	Publication bias	No	No	No	0.143	0.089	No	0.361	Weak
					Caucasian	0.0743	Allelic	Random	1	857 (362/495)	1.173 (0.816-1.686)	0.389	NA	NA	NA	NA															
							Dominant	Random	1	857 (362/495)	1.249 (0.849-1.837)	0.258	NA	NA	NA	NA															
							Recessive	Random	1	857 (362/495)	0.347 (0.039-3.123)	0.345	NA	NA	NA	NA															
6	ERCC4	rs2020955	C vs T	Brain	Caucasian	0.0034	Allelic	Fixed	3	2050 (565/1485)	1.035 (0.301-3.567)	0.956	0.0	0.859	0.384	1.000															
							Dominant	Fixed	3	2050 (565/1485)	1.163 (0.277-4.873)	0.837	0.0	0.939	0.650	1.000															
							Recessive	Fixed	3	2050 (565/1485)	1.503 (0.256-8.837)	0.652	0.0	0.951	0.725	1.000															
7	ERCC4	rs2276465	A vs G	Brain	Asian	0.3925	Allelic	Fixed	2	1456 (568/888)	0.996 (0.842-1.178)	0.960	0.0	0.351	NA	1.000															
							Dominant	Fixed	2	1456 (568/888)	0.990 (0.779-1.259)	0.935	0.0	0.597	NA	1.000															
							Recessive	Fixed	2	1456 (568/888)	1.002 (0.753-1.331)	0.992	14.3	0.280	NA	1.000															
8	ERCC4	rs2276466	G vs C	Brain (Glioma)	Asian	0.3243	Allelic	Fixed	2	930 (432/498)	1.332 (1.101-1.612)	0.003	0.0	0.878	NA	1.000	Moderate	BAA	660	B	A	A	No	NA	NA	NA	NA	1.000	No	0.065	Moderate
							Dominant	Fixed	2	930 (432/498)	1.336 (1.030-1.733)	0.029	0.0	0.881	NA	1.000	Moderate	BAA	509	B	A	A	No	NA	NA	NA	NA	1.000	No	0.4240	Weak
							Recessive	Fixed	2	930 (432/498)	1.553 (1.094-2.206)	0.014	0.0	0.917	NA	1.000	Moderate	BAA	151	B	A	A	No	NA	NA	NA	NA	1.000	No	0.385	Weak
9	ERCC4	rs3136038	T vs C	Brain	Asian	0.3475	Allelic	Fixed	2	930 (432/498)	1.042 (0.861-1.261)	0.673	0.0	0.972	NA	1.000															
							Dominant	Fixed	2	930 (432/498)	1.048 (0.808-1.360)	0.725	0.0	0.984	NA	1.000															
							Recessive	Fixed	2	930 (432/498)	1.064 (0.730-1.552)	0.747	0.0	0.973	NA	1.000															
10	ERCC4	rs6498486	C vs A	Brain	Asian	0.2787	Allelic	Fixed	2	1456 (568/888)	1.031 (0.870-1.221)	0.723	0.0	0.706	NA	1.000															
							Dominant	Fixed	2	1456 (568/888)	1.038 (0.836-1.288)	0.738	0.0	0.769	NA	1.000															
							Recessive	Fixed	2	1456 (568/888)	1.037 (0.722-1.490)	0.844	0.0	0.765	NA	1.000															
11	ERCC4	rs1799801	C vs T	Breast	Overall	0.2723	Allelic	Fixed	3	5241 (2194/3047)	0.955 (0.868-1.051)	0.350	42.9	0.173	0.033	0.296															
							Dominant	Fixed	3	5241 (2194/3047)	0.957 (0.849-1.079)	0.475	17.3	0.298	0.101	0.296															
							Recessive	Fixed	3	5241 (2194/3047)	0.899 (0.709-1.138)	0.375	21.3	0.280	0.148	1.000															
					Asian	0.2245	Allelic	Fixed	2	1956 (1024/932)	1.084 (0.919-1.278)	0.337	0.0	0.779	NA	NA															
							Dominant	Fixed	2	1956 (1024/932)	1.081 (0.886-1.319)	0.445	0.0	0.670	NA	NA															
							Recessive	Fixed	2	1956 (1024/932)	1.248 (0.781-1.995)	0.353	0.0	0.925	NA	NA															
					Caucasian	0.0688	Allelic	Fixed	1	3285 (1170/2115)	1.082 (0.844-1.387)	0.533	NA	NA	NA	NA															
							Dominant	Fixed	1	3285 (1170/2115)	0.893 (0.769-1.038)	0.141	NA	NA	NA	NA															
							Recessive	Fixed	1	3285 (1170/2115)	0.802 (0.608-1.059)	0.119	NA	NA	NA	NA															
12	ERCC4	rs1800067	A vs G	Breast	Overall	0.1040	Allelic	Random	12	17885 (9310/8575)	1.011 (0902-1.133)	0.850	37.5	0.091	0.858	0.945															
							Dominant	Fixed	12	17885 (9310/8575)	0.988 (0.901-1.083)	0.794	12.3	0.324	0.756	631.000															
							Recessive	Random	12	17885 (9310/8575)	1.725 (0.797-3.733)	0.166	59.2	0.009	0.191	0.858															
					Caucasian	0.0696	Allelic	Random	9	15228 (7936/7292)	1.042 (0.924-1.176)	0.502	38.6	0.111	NA	NA															
							Dominant	Fixed	9	15228 (7936/7292)	1.009 (0.917-1.109)	0.862	0.0	0.466	NA	NA															
							Recessive	Random	9	15228 (7936/7292)	2.041 (0.701-5.939)	0.191	61.9	0.010	NA	NA															
					African	0.0221	Allelic	Random	2	1823 (957/866)	0.609 (0.353-1.050)	0.074	0.0	0.381	NA	NA															
							Dominant	Fixed	2	1823 (957/866)	0.576 (0.330-1.003)	0.051	0.0	0.351	NA	NA															
							Recessive	Random	2	1823 (957/866)	2.671 (0.109-65.673)	0.548	NA	NA	NA	NA															
					Asian	0.8345	Allelic	Random	1	834 (417/417)	0.942 (0.730-1.217)	0.648	NA	NA	NA	NA															
							Dominant	Fixed	1	834 (417/417)	0.873 (0.524-1.455)	0.603	NA	NA	NA	NA															
							Recessive	Random	1	834 (417/417)	0.964 (0.708-1.312)	0.813	NA	NA	NA	NA															
13	ERCC4	rs2020955	C vs T	Breast	Overall	0.0940	Allelic	Fixed	3	5267 (2712/2555)	0.969 (0.807-1.164)	0.739	0.0	0.550	NA	1.000															
							Dominant	Fixed	3	5267 (2712/2555)	0.940 (0.756-1.168)	0.575	0.0	0.532	NA	1.000															
							Recessive	Fixed	3	5267 (2712/2555)	1.091 (0.680-1.751)	0.718	NA	NA	NA	NA															
14	ERCC4	rs2276465	A vs G	Breast	Asian	0.7562	Allelic	Fixed	2	1799 (878/921)	0.924 (0.795-1.075)	0.307	0.0	0.771	NA	1.000															
							Dominant	Fixed	2	1799 (878/921)	0.932 (0.703-1.235)	0.623	0.0	0.865	NA	1.000															
							Recessive	Fixed	2	1799 (878/921)	0.911 (0.752-1.102)	0.337	0.0	0.658	NA	1.000															
15	ERCC4	rs744154	C vs G	Breast	Overall	0.3517	Allelic	Fixed	4	81066 (41439/39627)	1.000 (0.978-1.023)	0.997	16.5	0.309	0.193	0.734															
							Dominant	Fixed	4	81066 (41439/39627)	0.994 (0.964-1.025)	0.717	0.0	0.759	0.535	1.000															
							Recessive	Fixed	4	81066 (41439/39627)	1.012 (0.968-1.058)	0.597	48.5	0.120	0.287	1.000															
					Caucasian	0.3598	Allelic	Fixed	2	75040 (38088/36952)	0.996 (0.972-1.019)	0.714	40.3	0.195	NA	NA															
							Dominant	Fixed	2	75040 (38088/36952)	0.990 (0.958-1.022)	0.534	0.0	0.965	NA	NA															
							Recessive	Fixed	2	75040 (38088/36952)	1.004 (0.959-1.050)	0.868	64.8	0.092	NA	NA															
					Asian	0.2412	Allelic	Fixed	1	4804 (2700/2104)	1.063 (0.968-1.167)	0.200	NA	NA	NA	NA															
							Dominant	Fixed	1	4804 (2700/2104)	1.057 (0.942-1.186)	0.345	NA	NA	NA	NA															
							Recessive	Fixed	1	4804 (2700/2104)	1.171 (0.923-1.485)	0.194	NA	NA	NA	NA															
					African	0.2469	Allelic	Fixed	1	1222 (651/571)	1.035 (0.862-1.244)	0.710	NA	NA	NA	NA															
							Dominant	Fixed	1	1222 (651/571)	0.981 (0.782-1.231)	0.870	NA	NA	NA	NA															
							Recessive	Fixed	1	1222 (651/571)	1.341 (0.845-2.128)	0.214	NA	NA	NA	NA															
16	ERCC4	rs1799802	T vs C	Colorectum	Caucasian	0.0034	Allelic	Fixed	2	2255 (1149/1106)	0.988 (0345-2.829)	0.982	0.0	0.585	NA	1.000															
							Dominant	Fixed	2	2255 (1149/1106)	0.988 (0.344-2.834)	0.982	0.0	0.584	NA	1.000															
							Recessive	Fixed	2	2255 (1149/1106)	NA	NA	NA	NA	NA	NA															
17	ERCC4	rs1800067	A vs G	Colorectum	Overall	0.0716	Allelic	Fixed	7	5295 (2427/2868)	0.949 (0.804-1.121)	0.593	0.0	0.777	0.712	0.548															
							Dominant	Fixed	7	5295 (2427/2868)	0.971 (0.814-1.158)	0.741	0.0	0.768	0.460	0.548															
							Recessive	Fixed	7	5295 (2427/2868)	0.600 (0.278-1.295)	0.193	45.2	0.140	0.753	1.000															
					Caucasian	0.0791	Allelic	Fixed	6	4564 (2133/2431)	0.952 (0.804-1.128)	0.571	0.0	0.666	NA	NA															
							Dominant	Fixed	6	4564 (2133/2431)	0.980 (0.818-1.174)	0.826	0.0	0.687	NA	NA															
							Recessive	Fixed	6	4564 (2133/2431)	0.512 (0.226-1.160)	0.109	52.2	0.124	NA	NA															
18	ERCC4	rs2276466	G vs C	Colorectum	Asian	0.2298	Allelic	Fixed	2	1003 (483/520)	1.158 (0.944-1.420)	0.160	0.0	0.917	NA	1.000															
							Dominant	Fixed	2	1003 (483/520)	1.159 (0.899-1.493)	0.255	0.0	0.878	NA	1.000															
							Recessive	Fixed	2	1003 (483/520)	1.252 (0.814-1.924)	0.306	0.0	0.974	NA	1.000															
19	ERCC4	rs6498486	C vs A	Colorectum	Asian	0.2462	Allelic	Fixed	2	2684 (1305/1379)	0.912 (0.804-1.035)	0.155	35.5	0.213	NA	1.000															
							Dominant	Fixed	2	2684 (1305/1379)	0.857 (0.734-1.000)	0.049	39.2	0.200	NA	1.000															
							Recessive	Fixed	2	2684 (1305/1379)	1.084 (0.781-1.504)	0.631	0.0	0.682	NA	1.000															
20	ERCC4	rs1799798	A vs G	Head and Neck	Caucasian	0.0855	Allelic	Fixed	2	4082 (1962/2120)	1.096 (0.941-1.276)	0.239	0.0	0.778	NA	1.000															
							Dominant	Fixed	2	4082 (1962/2120)	1.109 (0.943-1.304)	0.211	0.0	0.785	NA	1.000															
							Recessive	Fixed	2	4082 (1962/2120)	1.003 (0.375-2.682)	0.995	NA	NA	NA	NA															
21	ERCC4	rs1800067	A vs G	Head and Neck	Caucasian	0.0894	Allelic	Fixed	2	4082 (1962/2120)	1.000 (0.859-1.164)	0.998	0.3	0.316	NA	1.000															
							Dominant	Fixed	2	4082 (1962/2120)	1.000 (0.850-1.175)	0.997	11.2	0.288	NA	1.000															
							Recessive	Fixed	2	4082 (1962/2120)	1.006 (0.376-2.690)	0.991	NA	NA	NA	NA															
22	ERCC4	rs3136038	T vs C	Head and Neck	Overall	0.3111	Allelic	Fixed	3	4638 (2267/2371)	1.002 (0.918-1.095)	0.960	0.0	0.446	0.604	1.000															
							Dominant	Fixed	3	4638 (2267/2371)	1.060 (0.943-1.191)	0.331	0.0	0.743	0.478	0.296															
							Recessive	Fixed	3	4638 (2267/2371)	0.751 (0.573-0.985)	0.038	NA	NA	NA	NA															
					Caucasian	0.3085	Allelic	Fixed	2	4082 (1962/2120)	0992 (0.903-1.090)	0.869	20.3	0.263	NA	NA															
							Dominant	Fixed	2	4082 (1962/2120)	1.048 (0.926-1.186)	0.459	0.0	0.587	NA	NA															
							Recessive	Fixed	2	4082 (1962/2120)	0.751 (0.573-0.985)	0.038	NA	NA	NA	NA															
					African	0.3327	Allelic	Fixed	1	556 (305/251)	0.895 (0.795-1.008)	0.066	NA	NA	NA	NA															
							Dominant	Fixed	1	556 (305/251)	1.165 (0.814-1667)	0.405	NA	NA	NA	NA															
							Recessive	Fixed	1	556 (305/251)	NA	NA	NA	NA	NA	NA															
23	ERCC4	rs3136172	G vs A	Head and Neck	Overall	0.2219	Allelic	Fixed	2	2552 (1227/1325)	1.053 (0.922-1.202)	0.445	3.4	0.309	NA	1.000															
							Dominant	Fixed	2	2552 (1227/1325)	1.077 (0.919-1.261)	0.361	31.3	0.228	NA	1.000															
							Recessive	Fixed	2	2552 (1227/1325)	NA	NA	NA	NA	NA	NA															
24	ERCC4	rs744154	C vs G	Head and Neck	Overall	0.2853	Allelic	Fixed	2	2552 (1227/1325)	0.976 (0.864-1.103)	0.697	0.0	0.396	NA	1.000															
							Dominant	Fixed	2	2552 (1227/1325)	0.960 (0.819-1.125)	0.613	23.4	0.253	NA	1.000															
							Recessive	Fixed	2	2552 (1227/1325)	NA	NA	NA	NA	NA	NA															
25	ERCC4	rs2276465	A vs G	Laryngeal	Asian	0.6619	Allelic	Fixed	2	772 (386/386)	0.821 (0.667-1.011)	0.064	0.0	0.982	NA	1.000															
							Dominant	Fixed	2	772 (386/386)	0.768 (0.531-1.111)	0.161	0.0	0.945	NA	1.000															
							Recessive	Fixed	2	772 (386/386)	0.811 (0.611-1.077)	0.149	0.0	0.983	NA	1.000															
26	ERCC4	rs6498486	C vs A	Laryngeal	Asian	0.2938	Allelic	Fixed	3	1314 (657/657)	1.169 (0.991-1.380)	0.064	0.0	0.965	0.808	1.000															
							Dominant	Fixed	3	1314 (657/657)	1.179 (0.949-1.464)	0.136	0.0	0.986	0.715	1.000															
							Recessive	Fixed	3	1314 (657/657)	1.299 (0.920-1.836)	0.137	0.0	0.971	0.558	1.000															
27	ERCC4	rs1799801	C vs T	Lung	Overall	0.2760	Allelic	Fixed	2	2465 (866/1599)	1.035 (0.907-1,181)	0.611	31.1	0.228	NA	1.000															
							Dominant	Fixed	2	2465 (866/1599)	1.051 (0.889-1.242)	0.563	13.2	0.283	NA	1.000															
							Recessive	Fixed	2	2465 (866/1599)	1.020 (0.748-1.390)	0.920	0.0	0.453	NA	1.000															
28	ERCC4	rs1800067	A vs G	Lung	Overall	0.1042	Allelic	Fixed	4	3796 (1450/2346)	0.965 (0.828-1.124)	0.644	51.5	0.103	0.394	0.734															
							Dominant	Fixed	4	3796 (1450/2346)	0.969 (0.814-1.153)	0.721	37.4	0.187	0.475	0.734															
							Recessive	Random	4	3796 (1450/2346)	1.265 (0.443-3.612)	0.661	53.4	0.092	0.412	1.000															
					Caucasian	0.0692	Allelic	Fixed	2	2644 (967/1677)	1.125 (0.906-1.398)	0.286	0.0	0.660	NA	NA															
							Dominant	Fixed	2	2644 (967/1677)	1.082 (0.859-1.364)	0.331	0.0	0.701	NA	NA															
							Recessive	Random	2	2644 (967/1677)	2.780 (0.996-7.760)	0.051	0.0	0.978	NA	NA															
					Asian	0.3027	Allelic	Fixed	1	740 (370/370)	0.785 (0.628-0.985)	0.037	NA	NA	NA	NA															
							Dominant	Fixed	1	740 (370/370)	0.763 (0.571-1.018)	0.066	NA	NA	NA	NA															
							Recessive	Random	1	740 (370/370)	0.588 (0.316-1.094)	0.094	NA	NA	NA	NA															
					Latino	0.0552	Allelic	Fixed	1	412 (113/299)	1.304 (0.703-2.419)	0.399	NA	NA	NA	NA															
							Dominant	Fixed	1	412 (113/299)	1.376 (0.723-2,619)	0.331	NA	NA	NA	NA															
							Recessive	Random	1	412 (113/299)	0.877 (0.035-21.677)	0.936	NA	NA	NA	NA															
29	ERCC4	rs6498486	C vs A	Pancreas	Asian	0.2625	Allelic	Fixed	2	941 (441/500)	1.146 (0.936-1.404)	0.188	0.0	0.855	NA	1.000															
							Dominant	Fixed	2	941 (441/500)	1.154 (0.893-1.493)	0.274	0.0	0.915	NA	1.000															
							Recessive	Fixed	2	941 (441/500)	1.276 (0.803-2.027)	0.302	0.0	0.721	NA	1.000															
30	ERCC4	rs1799801	C vs T	Prostate	Overall	0.2532	Allelic	Fixed	3	3363 (1711/1652)	1.038 (0.928-1.162)	0.510	0.0	0.408	0.340	1.000															
							Dominant	Fixed	3	3363 (1711/1652)	1.037 (0.901-1.194)	0.608	0.0	0.460	0.348	1.000															
							Recessive	Fixed	3	3363 (1711/1652)	1.089 (0.828-1.433)	0.540	0.0	0.705	0.273	1.000															
					African	0.1355	Allelic	Fixed	2	789 (403/386)	1.235 (0.930-1.640)	0.144	0.0	0.785	NA	NA															
							Dominant	Fixed	2	789 (403/386)	1.241 (0.901-1.709)	0.186	0.0	0.815	NA	NA															
							Recessive	Fixed	2	789 (403/386)	1.619 (0.613-4.273)	0.331	0.0	0.986	NA	NA															
					Caucasian	0.2892	Allelic	Fixed	1	2574 (1308/1266)	1.005 (0.890-1.136)	0.932	NA	NA	NA	NA															
							Dominant	Fixed	1	2574 (1308/1266)	0.994 (0.850-1.162)	0.937	NA	NA	NA	NA															
							Recessive	Fixed	1	2574 (1308/1266)	1.051 (0.790-1.400)	0.732	NA	NA	NA	NA															
31	ERCC4	rs1800067	A vs G	Prostate	Overall	0.0827	Allelic	Fixed	2	2808 (1457/1351)	0.993 (0.815-1.210)	0.943	0.0	0.532	NA	1.000															
							Dominant	Fixed	2	2808 (1457/1351)	0.947 (0.767-1.169)	0.613	0.0	0.482	NA	1.000															
							Recessive	Fixed	2	2808 (1457/1351)	2.613 (0.929-7.352)	0.069	NA	NA	NA	NA															
32	ERCC4	rs1800067	A vs G	Skin	Caucasian	0.0866	Allelic	Fixed	3	3036 (1713/1323)	1.007 (0.836-1.213)	0.938	0.0	0.428	0.940	1.000															
							Dominant	Fixed	3	3036 (1713/1323)	0.986 (0.809-1.202)	0.888	0.0	0.375	0.962	1.000															
							Recessive	Fixed	3	3036 (1713/1323)	1.855 (0.623-5.521)	0.267	0.0	0.866	0.162	0.296															
33	ERCC4	rs1799797	A vs T	Skin	Caucasian	0.3115	Allelic	Random	2	1314 (725/589)	0.837 (0.579-1.210)	0.345	70.0	0.068	NA	1.000															
							Dominant	Random	2	1314 (725/589)	0.772 (0.505-1.180)	0.231	63.9	0.096	NA	1.000															
							Recessive	Fixed	2	1314 (725/589)	1.019 (0.705-1.473)	0.920	8.1	0.297	NA	1.000															
34	ERCC4	rs1799801	C vs T	Skin	Overall	0.3074	Allelic	Fixed	4	2755 (1551/1204)	0.997 (0.884-1.125)	0.966	18.6	0.297	0.418	1.000															
							Dominant	Random	4	2755 (1551/1204)	0.967 (0.732-1.278)	0.816	63.4	0.042	0.433	0.734															
							Recessive	Fixed	4	2755 (1551/1204)	0.955 (0.736-1.240)	0.730	0.0	0.401	0.999	1.000															
					Caucasian	0.3102	Allelic	Fixed	3	2463 (1405/1058)	0.993 (0.873-1.129)	0.912	45.1	0.162	NA	NA															
							Dominant	Random	3	2463 (1405/1058)	0950 (0.663-1.362)	0.781	75.6	0.017	NA	NA															
							Recessive	Fixed	3	2463 (1405/1058)	0.931 (0.707-1.227)	0.613	24.0	0.268	NA	NA															
					MIxed	0.2877	Allelic	Fixed	1	292 (146/146)	1.034 (0.723-1.477)	0.855	NA	NA	NA	NA															
							Dominant	Random	1	292 (146/146)	1.000 (0.632-1.582)	1.000	NA	NA	NA	NA															
							Recessive	Fixed	1	292 (146/146)	1.184 (0.528-2.656)	0.681	NA	NA	NA	NA															
35	ERCC4	rs1799801	C vs T	Stomach	Overall	0.4027	Allelic	Random	3	2796 (1327/1469)	0.847 (0.667-1.074)	0.171	57.5	0.095	0.661	1.000															
							Dominant	Fixed	3	2796 (1327/1469)	0.755 (0.614-0.930)	0.008	42.2	0.177	0.954	1.000	Moderate	ABA	1281	A	B	A	No	No	No	No	0.954	1.000	No	0.151	Moderate
							Recessive	Fixed	3	2796 (1327/1469)	0.977 (0.755-1.264)	0.859	0.0	0.373	0.448	1.000															
					Asian	0.4660	Allelic	Random	2	1504 (681/823)	0.914 (0.687-1.215)	0.534	62.9	0.101	NA	NA															
							Dominant	Fixed	2	1504 (681/823)	0.839 (0.658-1.070)	0.157	0.0	0.371	NA	NA															
							Recessive	Fixed	2	1504 (681/823)	0.981 (0.743-1.297)	0.894	49.1	0.161	NA	NA															
					Caucasian	0.3158	Allelic	Random	1	1292 (646/646)	0.698(0.505-0.963)	0.029	NA	NA	NA	NA															
							Dominant	Fixed	1	1292 (646/646)	0.567 (0.378-0.849)	0.006	NA	NA	NA	NA															
							Recessive	Fixed	1	1292 (646/646)	0.952 (0.484-1.872)	0.886	NA	NA	NA	NA															
36	ERCC4	rs2276466	G vs C	Stomach	Asian	0.2153	Allelic	Fixed	2	4636 (2267/2369)	1.051 (0.953-1.160)	0.319	12.0	0.286	NA	1.000															
							Dominant	Fixed	2	4636 (2267/2369)	1.077 (0.957-1.212)	0.216	48.4	0.164	NA	1.000															
							Recessive	Fixed	2	4636 (2267/2369)	0.987 (0.751-1.295)	0.922	0.0	0.835	NA	1.000															
37	ERCC4	rs6498486	C vs A	Stomach	Asian	0.2361	Allelic	Fixed	3	3970 (1787/2120)	0.982 (0.883-1.092)	0.736	0.0	0.743	0.487	1.000															
							Dominant	Fixed	3	3970 (1787/2120)	0.969 (0.852-1.101)	0.626	0.0	0.632	0.584	1.000															
							Recessive	Fixed	3	3970 (1787/2120)	1.026 (0.770-1.367)	0.862	0.0	0.946	0.581	0.296															
38	ERCC4	rs744154	C vs G	Stomach	Asian	0.4854	Allelic	Fixed	2	1504 (681/823)	0.790 (0.666-0.937)	0.007	62.2	0.104	NA	1.000	Weak	ACA	1442	A	C	A	No	NA	NA	NA	NA	1.000	No	0.117	Weak
							Dominant	Fixed	2	1504 (681/823)	0.681 (0.535-0.866)	0.020	37.6	0.2060	NA	1.000	Moderate	BBA	951	B	B	A	No	NA	NA	NA	NA	1.000	No	0.055	Moderate
							Recessive	Fixed	2	1504 (681/823)	0.890 (0.671-1.181)	0.419	0.0	0.505	NA	1.000															
39	ERCC4	rs1799801	C vs T	Uterus	Caucasian	0.2890	Allelic	Fixed	2	2369 (1154/1215)	0.978 (0.859-1.113)	0.736	0.0	0.972	NA	1.000															
							Dominant	Fixed	2	2369 (1154/1215)	1.021 (0.865-1.205)	0.809	0.0	0.915	NA	1.000															

							Recessive	Fixed	2	2369 (1154/1215)	0.818 (0.598-1.118)	0.207	0.0	0.898	NA	1.000															
40	ERCC4	rs1800067	A vs G	Uterus	Caucasian	0.0670	Allelic	Fixed	2	2369 (1154/1215)	1.186 (0.945-1.490)	0.141	0.0	0.894	NA	1.000															
							Dominant	Fixed	2	2369 (1154/1215)	1.236 (0.971-1.572)	0.085	0.0	0.911	NA	1.000															
							Recessive	Fixed	2	2369 (1154/1215)	0.596 (0.174-2.043)	0.410	0.0	0.959	NA	1.000															
41	ERCC5	rs17655	C vs G	Bladder	Overall	0.2615	Allelic	Random	7	5476 (2704/2772)	1.095 (0.900-1.331)	0.364	73.2	0.001	0.478	0.368															
							Dominant	Random	7	5476 (2704/2772)	1.121 (0.870-1.444)	0.377	73.8	0.001	0.459	0.548															
							Recessive	Random	7	5476 (2704/2772)	1.082 (0.778-1.506)	0.639	48.7	0.069	0.866	0.368															
					Asian	0.2698	Allelic	Randon	1	390 (112/278)	2.105 (1.523-2.910)	0.000	NA	NA	NA	NA															
							Dominant	Random	1	390 (112/278)	2.921 (1.851-4.608)	0.000	NA	NA	NA	NA															
							Recessive	Random	1	390 (112/278)	1.608 (0.933-2.771)	0.087	NA	NA	NA	NA															
					African	0.3506	Allelic	Random	2	636 (318/318)	1.007 (0.799-1.268)	0.953	0.0	0.944	NA	NA															
							Dominant	Random	2	636 (318/318)	0.879 (0.641-1.204)	0.422	0.0	0.767	NA	NA															
							Recessive	Random	2	636 (318/318)	1.342 (0.850-2.119)	0.206	0.0	0.668	NA	NA															
					Caucasian	0.2466	Allelic	Random	4	4171 (2111/2060)	0.980 (0.887-1.083)	0.691	0.0	0.433	NA	NA															
							Dominant	Random	4	4171 (2111/2060)	0.994 (0.879-1.124)	0.927	0.0	0.673	NA	NA															
							Recessive	Random	4	4171 (2111/2060)	0.836 (0.497-1.406)	0.500	56.0	0.078	NA	NA															
42	ERCC5	rs17655	C vs G	Blood	Overall	0.2597	Allelic	Random	7	5100 (2592/2508)	1.176 (1.017-1.360)	0.029	50.2	0.061	0.219	0.133	Weak	ACA	2457	A	C	A	No	No	No	None	0.219	0.133	No	0.354	Weak
							Dominant	Fixed	7	5100 (2592/2508)	1.169 (1.041-1.313)	0.009	37.3	0.144	0.259	0.230	Moderate	ABA	2401	A	B	A	No	No	No	None	0.259	0.230	No	0.138	Moderate
							Recessive	Fixed	7	5100 (2592/2508)	1.178 (0.955-1.451)	0.125	23.9	0.247	0.387	1.000															
					Caucasian	0.2517	Allelic	Random	4	2114 (1014/1100)	1.298 (1.007-1.671)	0.044	59.0	0.062	0.696	0.734	Weak	ACA	1097	A	C	A	No	None	No	None	0.696	0.734	No	0.484	Weak
							Dominant	Fixed	4	2114 (1014/1100)	1.285 (1.065-1.550)	0.009	61.0	0.051	0.537	0.734	Weak	BCA	888	B	C	A	No	None	No	None	0.537	0.734	No	0.149	Weak
							Recessive	Fixed	4	2114 (1014/1100)	1.511 (1.093-2.090)	0.013	0.0	0.472	0.384	0.308	Moderate	BAA	209	B	A	A	No	None	No	None	0.384	0.308	No	0.332	Weak
					African	0.3398	Allelic	Random	1	412 (206/206)	1.022 (0.766-1.363)	0.883	NA	NA	NA	NA															
							Dominant	Fixed	1	412 (206/206)	1.040 (0.704-1.537)	0.842	NA	NA	NA	NA															
							Recessive	Fixed	1	412 (206/206)	1.000 (0.542-1.846)	1.000	NA	NA	NA	NA															
					Mixed	0.2526	Allelic	Random	2	2441 (1299/1142)	1.069 (0.936-1.220)	0.325	2.3	0.312	NA	NA															
							Dominant	Fixed	2	2441 (1299/1142)	1.112 (0.948-1.306)	0.193	0.0	0.523	NA	NA															
							Recessive	Fixed	2	2441 (1299/1142)	0.980 (0.721-1.333)	0.900	31.2	0.228	NA	NA															
43	ERCC5	rs17655	C vs G	Brain	Overall	0.2815	Allelic	Random	5	5814 (1877/3937)	1.080 (0.899-1.298)	0.411	65.5	0.021	0.702	1.000															
							Dominant	Fixed	5	5814 (1877/3937)	1.066 (0.950-1.195)	0.277	44.8	0.123	0.587	0.462															
							Recessive	Random	5	5814 (1877/3937)	1.089 (0.644-1.841)	0.750	72.3	0.006	0.910	0.806															
					Caucasian	0.2831	Allelic	Random	4	5059 (1580/3479)	1,017 (0.839-1.233)	0.862	56.7	0.074	NA	NA															
							Dominant	Fixed	4	5059 (1580/3479)	1.042 (0.920-1.179)	0.518	53.0	0.094	NA	NA															
							Recessive	Random	4	5059 (1580/3479)	0.920 (0.707-1.197)	0.535	10.0	0.343	NA	NA															
					Asian	0.2687	Allelic	Random	1	755 (297/458)	1.330 (1.057-1.674)	0.015	NA	NA	NA	NA															
							Dominant	Fixed	1	755 (297/458)	1.215 (0.901-1.637)	0.201	NA	NA	NA	NA															
							Recessive	Random	1	755 (297/458)	2.588 (1.478-4.533)	0.001	NA	NA	NA	NA															
44	ERCC5	rs1047768	C vs T	Breast	Asian	0.2250	Allelic	Random	2	825 (425/400)	2.255 (0.475-10.696)	0.306	87.6	0.004	NA	1.000															
							Dominant	Random	2	825 (425/400)	2.492 (0.431-14.422)	0.308	86.8	0.006	NA	1.000															
							Recessive	Fixed	2	825 (425/400)	1.320 (0.734-2.374)	0.353	0.0	0.426	NA	1.000															
45	ERCC5	rs1047769	G vs A	Breast	Overall	0.0345	Allelic	Fixed	2	2657 (1097/1560)	0.959 (0.706-1.303)	0.790	39.6	0.198	NA	1.000															
							Dominant	Fixed	2	2657 (1097/1560)	0.958 (0.701-1.308)	0.786	41.7	0.190	NA	1.000															
							Recessive	NA	2	2657 (1097/1560)	NA	NA	NA	NA	NA	1.000															
					Caucasian	0.0328	Allelic	Fixed	1	1941 (858/1083)	1.076 (0.758-1.526)	0.682	NA	NA	NA	NA															
							Dominant	Fixed	1	1941 (858/1083)	1.079 (0.756-1.540)	0.677	NA	NA	NA	NA															
							Recessive	NA	1	1941 (858/1083)	NA	NA	NA	NA	NA	NA															
					Mixed	0.0383	Allelic	Fixed	1	716 (239/477)	0.658 (0.339-1.277)	0.216	NA	NA	NA	NA															
							Dominant	Fixed	1	716 (239/477)	0.649 (0.331-1.308)	0.208	NA	NA	NA	NA															
							Recessive	NA	1	716 (239/477)	NA	NA	NA	NA	NA	NA															
46	ERCC5	rs17655	C vs G	Breast	Overall	0.3003	Allelic	Random	15	17222 (8341/8881)	1.039 (0.959-1.125)	0.355	51.6	0.011	0.253	0.488															
							Dominant	Random	15	17222 (8341/8881)	1.077 (0.973-1.192)	0.153	49.5	0.018	0.999	0.743															
							Recessive	Fixed	15	17222 (8341/8881)	1.008 (0.902-1.127)	0.884	30.3	0.128	0.208	0.322															
					Asian	0.5058	Allelic	Random	4	2630 (1272/1358)	0.947 (0.774-1.159)	0.600	57.3	0.071	NA	NA															
							Dominant	Random	4	2630 (1272/1358)	0.970 (0.811-1.159)	0.737	0.0	0.382	NA	NA															
							Recessive	Fixed	4	2630 (1272/1358)	0.902 (0.741-1.098)	0.304	55.7	0.080	NA	NA															
					Caucasian	0.2392	Allelic	Random	8	12053 (5873/6180)	1.071 (0.962-1.195)	0.109	58.8	0.017	NA	NA															
							Dominant	Random	8	12053 (5873/6180)	1.104 (0.950-1.283)	0.198	67.6	0.003	NA	NA															
							Recessive	Fixed	8	12053 (5873/6180)	1.087 (0.923-1.280)	0.320	0.0	0.570	NA	NA															
					African	0.4292	Allelic	Random	2	1823 (957/866)	0.979 (0.712-1.347)	0.898	46.2	0.173	NA	NA															
							Dominant	Random	2	1823 (957/866)	1.169 (0.944-1.449)	0.152	0.0	0.603	NA	NA															
							Recessive	Fixed	2	1823 (957/866)	0.939 (0.727-1.214)	0.632	65.7	0.088	NA	NA															
					Mixed	0.2193	Allelic	Random	1	716 (239/477)	1.115 (0.859-1.447)	0.413	NA	NA	NA	NA															
							Dominant	Random	1	716 (239/477)	1.041 (0.758-1.430)	0.804	NA	NA	NA	NA															
							Recessive	Fixed	1	716 (239/477)	1.731 (0.889-3.369)	0.107	NA	NA	NA	NA															
47	ERCC5	rs2094258	T vs C	Breast	Asian	0.5249	Allelic	Random	2	1264 (645/619)	1.083 (0.686-1.709)	0.733	85.7	0.008	NA	1.000															
							Dominant	Random	2	1264 (645/619)	1.102 (0.558-2.176)	0.779	72.1	0.059	NA	1.000															
							Recessive	Random	2	1264 (645/619)	1.113 (0.629-1.968)	0.714	78.4	0.031	NA	1.000															
48	ERCC5	rs751402	G vs A	Breast	Asian	0.6390	Allelic	Random	4	1743 (901/842)	1.373 (0.802-2.349)	0.248	91.3	0.000	0.085	0.089															
							Dominant	Random	4	1743 (901/842)	1.116 (0.698-1.811)	0.657	56.7	0.074	0.042	0.308															
							Recessive	Random	4	1743 (901/842)	1.350 (0.649-2.805)	0.422	91.1	0.000	0.279	0.734															
49	ERCC5	rs873601	A vs G	Breast	Overall	0.3405	Allelic	Fixed	2	1366 (564/802)	1.034 (0.878-1.219)	0.686	0.0	0.469	NA	1.000															
							Dominant	Fixed	2	1366 (564/802)	1.030 (0.820-1.292)	0.801	0.0	0.358	NA	1.000															
							Recessive	Fixed	2	1366 (564/802)	1.069 (0.783-1.461)	0.674	0.0	0.946	NA	1.000															
					Asian	0.2756	Allelic	Fixed	1	650 (325/325)	1.091 (0.877-1.358)	0.435	NA	NA	NA	NA															
							Dominant	Fixed	1	650 (325/325)	1.152 (0.828-1.602)	0.401	NA	NA	NA	NA															
							Recessive	Fixed	1	650 (325/325)	1.077 (0.738-1.572)	0.700	NA	NA	NA	NA															
					Mixed	0.3422	Allelic	Fixed	1	716 (239/477)	0.966 (0.753-1.238)	0.783	NA	NA	NA	NA															
							Dominant	Fixed	1	716 (239/477)	0.930 (0.680-1,273)	0.652	NA	NA	NA	NA															
							Recessive	Fixed	1	716 (239/477)	1.052 (0.605-1.830)	0.856	NA	NA	NA	NA															
50	ERCC5	rs1047768	C vs T	Colorectum	Overall	0.3810	Allelic	Random	4	8894 (3890/5004)	0.978 (0.867-1.102)	0.714	62.5	0.046	0.515	0.308															
							Dominant	Fixed	4	8894 (3890/5004)	0.989 (0.901-1.086)	0.816	28.5	0.241	0.454	0.308															
							Recessive	Random	4	8894 (3890/5004)	1.009 (0.784-1.300)	0.943	66.8	0.029	0.984	1.000															
					Asian	0.2815	Allelic	Random	2	55889 (2755/2834)	1.054 (0.971-1.145)	0.208	0.0	0.651	NA	NA															
							Dominant	Fixed	2	55889 (2755/2834)	1.027 (0.924-1.141)	0.620	0.0	0.926	NA	NA															
							Recessive	Random	2	55889 (2755/2834)	1.218 (1.006-1.474)	0.044	0.0	0.367	NA	1.000	Moderate	BAA	461	B	A	A	No	NA	NA	NA	NA	1.000	No	0.452	Weak
					Caucasian	0.5512	Allelic	Fixed	2	3305 (1135/2170)	0.876 (0.774-0.991)	0.036	44.5	0.180	NA	1.000	Weak	BBC	707	B	B	C	Low OR	NA	NA	NA	NA	1.000	Yes	0.402	Weak
							Dominant	Fixed	2	3305 (1135/2170)	0.859 (0.701-1.052)	0.140	42.9	0.186	NA	NA															
							Recessive	Random	2	3305 (1135/2170)	0.822 (0.672-1.004)	0.055	0.0	0.480	NA	NA															
51	ERCC5	rs1047769	G vs A	Colorectum	Caucasian	0.0335	Allelic	Fixed	2	4148 (1530/2618)	0.826 (0.600-1.136)	0.240	37.1	0.207	NA	1.000															
							Dominant	Fixed	2	4148 (1530/2618)	0.829 (0.598-1.149)	0.261	31.5	0.227	NA	1.000															
							Recessive	Fixed	2	4148 (1530/2618)	0.708 (0.105-5.779)	0.808	0.0	0.791	NA	1.000															
52	ERCC5	rs17655	C vs G	Colorectum	Overall	0.3667	Allelic	Fixed	13	17943 (8068/9875)	1.053 (1.006-1.102)	0.027	18.3	0.259	0.852	0.951	Weak	AAC	12838	A	A	C	HWE, Low OR	None	No	Yes	0.852	0.951	Yes	0.331	Weak
							Dominant	Random	13	17943 (8068/9875)	1.132 (1.020-1.255)	0.019	53.3	0.012	0.842	0.855	Weak	ACC	10283	A	C	C	Low OR	None	No	No	0.842	0.855	Yes	0.260	Weak
							Recessive	Fixed	13	17943 (8068/9875)	0968 (0.888-1.057)	0.470	2.1	0.425	0.852	0.760															
					Asian	0.4628	Allelic	Fixed	5	10006 (4916/5090)	1.053 (0.996-1.113)	0.070	66.3	0.018	NA	NA															
							Dominant	Random	5	10006 (4916/5090)	1.149 (0.944-1.399)	0.167	79.6	0.001	NA	NA															
							Recessive	Fixed	5	10006 (4916/5090)	0.987 (0.896-1.087)	0.793	10.3	0.348	NA	NA															
					Caucasian	0.2283	Allelic	Fixed	7	7206 (2858/4348)	1.062 (0.976-1.156)	0.165	0.0	0.870	NA	NA															
							Dominant	Random	7	7206 (2858/4348)	1.118 (1.006-1.242)	0.038	0.0	0.431	0.907	0.548	weak	AAC	3704	A	A	C	HWE, Low OR	None	No	Yes	0.907	0.548	Yes	0.417	Weak
							Recessive	Fixed	7	7206 (2858/4348)	0.916 (0.730-1.151)	0.454	11.9	0.339	NA	NA															
					African	0.4464	Allelic	Fixed	1	731 (294/437)	0.982 (0.770-1.253)	0.886	NA	NA	NA	NA															
							Dominant	Random	1	731 (294/437)	1.127 (0.776-1.637)	0.529	NA	NA	NA	NA															
							Recessive	Fixed	1	731 (294/437)	0.802 (0.517-1.244)	0.324	NA	NA	NA	NA															
53	ERCC5	rs2094258	T vs C	Colorectum	Asian	0.3394	Allelic	Fixed	2	5589 (2755/2834)	1.128 (1.043-1.219)	0.002	9.0	0.295	NA	1.000	Weak	AAC	3927	A	A	C	Low OR	NA	NA	NA	NA	1.000	Yes	0.043	Moderate
							Dominant	Fixed	2	5589 (2755/2834)	1.141 (1.025-1.270)	0.015	0.0	0.706	NA	1.000	Weak	AAC	3232	A	A	C	Low OR	NA	NA	NA	NA	1.000	Yes	0.231	Weak
							Recessive	Fixed	2	5589 (2755/2834)	1.232 (1.051-1.445)	0.010	59.8	0.115	NA	1.000	Weak	BCA	695	B	C	A	No	NA	NA	NA	NA	1.000	No	0.165	Weak
54	ERCC5	rs2227869	C vs G	Colorectum	Caucasian	0.0572	Allelic	Fixed	2	4148 (1530/2618)	1.010 (0.796-1.282)	0.935	0.0	0.346	NA	1.000															
							Dominant	Fixed	2	4148 (1530/2618)	0.994 (0.775-1.275)	0.964	0.0	0.385	NA	1.000															
							Recessive	Fixed	2	4148 (1530/2618)	2.055 (0.411-10.272)	0.380	0.0	0.517	NA	1.000															
55	ERCC5	rs2296147	C vs T	Colorectum	Asian	0.2136	Allelic	Fixed	2	5589 (2755/2834)	1.027 (0.939-1.124)	0.559	0.0	0.981	NA	1.000															
							Dominant	Fixed	2	5589 (2755/2834)	1.008 (0.905-1.122)	0.888	0.0	0.737	NA	1.000															
							Recessive	Fixed	2	5589 (2755/2834)	1.191 (0.920-1.541)	0.185	0.0	0.427	NA	1.000															
56	ERCC5	rs873601	A vs G	Colorectum	Asian	0.4698	Allelic	Random	2	5589 (2755/2834)	1.043 (0.842-1.291)	0.701	85.6	0.008	NA	1.000															
							Dominant	Fixed	2	5589 (2755/2834)	1.094 (0.971-1.232)	0.141	45.0	0.177	NA	1.000															
							Recessive	Random	2	5589 (2755/2834)	1.052 (0.693-1.596)	0.813	89.2	0.002	NA	1.000															
57	ERCC5	rs2094258	T vs C	Esophagus	Asian	0.4052	Allelic	Fixed	2	4292 (1672/2620)	1.045 (0.954-1.145)	0.342	0.0	0.821	NA	1.000															
							Dominant	Fixed	2	4292 (1672/2620)	1.058 (0.927-1.207)	0.405	0.0	0.710	NA	1.000															
							Recessive	Fixed	2	4292 (1672/2620)	1.062 (0.897-1.258)	0.484	0.0	0.965	NA	1.000															
58	ERCC5	rs2296147	T vs C	Esophagus	Asian	0.2550	Allelic	Fixed	2	4292 (1672/2620)	0.825 (0.741-0.919)	0.000	0.0	0.531	NA	1.000	Strong	AAA	2019	A	A	A	No	NA	NA	NA	NA	1.000	No	0.009	Strong
58	ERCC5	rs2296147	T vs C	Esophagus	Asian	0.2550	Dominant	Fixed	2	4292 (1672/2620)	0.783 (0.687-0.892)	0.000	0.0	0.868	NA	1.000	Strong	AAA	1767	A	A	A	No	NA	NA	NA	NA	1.000	No	0.004	Strong
							Recessive	Fixed	2	4292 (1672/2620)	0.839 (0.633-1.111)	0.220	62.4	0.103	NA	1.000															
59	ERCC5	rs873601	T vs C	Esophagus	Asian	0.3885	Allelic	Random	2	4292 (1672/2620)	1.141 (0.849-1.533)	0.382	90.4	0.001	NA	1.000															
							Dominant	Random	2	4292 (1672/2620)	1.195 (0.815-1.751)	0.362	87.5	0.005	NA	1.000															
							Recessive	Random	2	4292 (1672/2620)	1.158 (0.801-1.675)	0.435	78.8	0.030	NA	1.000															
60	ERCC5	rs17655	C vs G	Head and Neck	Overall	0.4481	Allelic	Random	6	6772 (2919/3853)	0.889 (0.764-1.036)	0.131	70.6	0.004	0.062	0.133															
							Dominant	Random	6	6772 (2919/3853)	0.935 (0.773-1.131)	0.490	52.9	0.060	0.371	0.452															
							Recessive	Random	6	6772 (2919/3853)	0.787 (0.627-0.989)	0.040	58.8	0.033	0.032	0.060	Weak	ACC	1780	A	C	C	udy, HWE, Publicati	Yes	No	Yes	0.032	0.06	No	0.451	Weak
					Caucasian	0.4100	Allelic	Random	3	4703 (2136/2567)	1.010 (0.915-1.115)	0.846	0.0	0.840	NA	NA															
							Dominant	Random	3	4703 (2136/2567)	1.063 (0.921-1.225)	0.404	0.0	0.552	NA	NA															
							Recessive	Random	3	4703 (2136/2567)	0.908 (0.712-1.159)	0.439	28.4	0.248	NA	NA															
					Asian	0.5252	Allelic	Random	3	2069 (783/1286)	0.745 (0.542-1.023)	0.069	80.4	0.006	NA	NA															
							Dominant	Fixed	3	2069 (783/1286)	0.796 (0.649-0.976)	0.028	54.0	0.114	0.031	0.296	Weak	ACC	1502	A	C	C	First study, HWE	Yes	No	Yes	0.031	0.296	No	0.360	Weak
							Recessive	Random	3	2069 (783/1286)	0.703 (0.476-1.039)	0.077	70.2	0.035	NA	NA															
61	ERCC5	rs1047768	C vs T	Head and Neck	Overall	0.5827	Allelic	Fixed	3	3440 (1517/1923)	0.966 (0.876-1.066)	0.497	0.0	0.890	0.536	0.296															
							Dominant	Fixed	3	3440 (1517/1923)	0.969 (0.810-1.159)	0.733	0.0	0.928	0.937	1.000															
							Recessive	Fixed	3	3440 (1517/1923)	0.948 (0.820-1.096)	0.468	0.0	0.714	0.257	0.296															
					Caucasian	0.5867	Allelic	Fixed	2	3020 (1307/1713)	0.976 (0.878-1.084)	0.646	0.0	0.941	NA	NA															
							Dominant	Fixed	2	3020 (1307/1713)	0.978 (0.805-1.189)	0.824	0.0	0.758	NA	NA															
							Recessive	Fixed	2	3020 (1307/1713)	0.961 (0.823-1.123)	0.619	0.0	0.880	NA	NA															
					Asian	0.5500	Allelic	Fixed	1	420 (210/210)	0.909 (0.693-1.192)	0.489	NA	NA	NA	NA															
							Dominant	Fixed	1	420 (210/210)	0.922 (0.583-1.456)	0.726	NA	NA	NA	NA															
							Recessive	Fixed	1	420 (210/210)	0.854 (0.563-1.295)	0.457	NA	NA	NA	NA															
62	ERCC5	rs17655	C vs G	Laryngeal	Overall	0.3732	Allelic	Random	3	1667 (634/1033)	0.751 (0.539-1.047)	0.091	77.8	0.011	0.211	0.296															
							Dominant	Random	3	1667 (634/1033)	0.777 (0.488-1.236)	0.286	75.3	0.017	0.081	0.296															
							Recessive	Fixed	3	1667 (634/1033)	0.571 (0.533-0.753)	0.000	0.0	0.933	0.730	1.000	Weak	BAC	328	B	A	C	First study, HWE	Yes	No	Yes	0.730	1.000	No	0.010	Moderate
					Caucasian	0.2349	Allelic	Random	1	895 (248/647)	1.028 (0.806-1.311)	0.824	NA	NA																	
							Dominant	Random	1	895 (248/647)	1.153 (0.858-1.549)	0.344	NA	NA																	
							Recessive	Fixed	1	895 (248/647)	0.550 (0.252-1.197)	0.132	NA	NA																	
62	ERCC5	rs17655	C vs G	Laryngeal	Asian	0.6049	Allelic	Random	2	772 (386/386)	0.636 (0.520-0.779)	0.000	0.0	0.683	NA	1.000	Moderate	BAA	849	B	A	A	No	NA	NA	NA	NA	1.000	No	0.001	Strong
62	ERCC5	rs17655	C vs G	Laryngeal	Asian	0.6049	Dominant	Random	2	772 (386/386)	0.613 (0.444-0.847)	0.003	0.0	0.770	NA	1.000	Moderate	BAA	566	B	A	A	No	NA	NA	NA	NA	1.000	No	0.158	Moderate
62	ERCC5	rs17655	C vs G	Laryngeal	Asian	0.6049	Recessive	Fixed	2	772 (386/386)	0.574 (0.427-0.772)	0.000	0.0	0.720	NA	1.000	Moderate	BAA	283	B	A	A	No	NA	NA	NA	NA	1.000	No	0.025	Strong
63	ERCC5	rs1047768	C vs T	Lung	Overall	0.5654	Allelic	Random	4	3693 (1383/2310)	1.093 (0.868-1.377)	0.448	75.1	0.007	0.899	1.000															
							Dominant	Random	4	3693 (1383/2310)	1.184 (0.801-1.752)	0.397	76.1	0.006	0.843	1.000															
							Recessive	Fixed	4	3693 (1383/2310)	1.037 (0.897-1.199)	0.624	18.1	0.300	0.944	1.000															
					Caucasian	0.5804	Allelic	Random	3	3449 (1261/2188)	1.059 (0.805-1.394)	0.680	82.5	0.003	NA	NA															
							Dominant	Random	3	3449 (1261/2188)	1.110 (0676-1.824)	0.680	83.2	0.003	NA	NA															
							Recessive	Fixed	3	3449 (1261/2188)	1.037 (0.895-1.202)	0.626	45.4	0.160	NA	NA															
					Asian	0.2768	Allelic	Random	1	244 (122/122)	1.265 (0.848-1.887)	0.248	NA	NA	NA	NA															
							Dominant	Random	1	244 (122/122)	1.473 (0.876-2.477)	0.144	NA	NA	NA	NA															
							Recessive	Fixed	1	244 (122/122)	1.025 (0.459-2.290)	0.952	NA	NA	NA	NA															
64	ERCC5	rs17655	C vs G	Lung	Overall	0.4252	Allelic	Random	11	9728 (4284/5444)	1.078 (0.961-1.209)	0.199	65.2	0.001	0.383	0.755															
							Dominant	Random	11	9728 (4284/5444)	1.139 (0.976-1.328)	0.098	52.9	0.020	0.805	1.000															
							Recessive	Random	11	9728 (4284/5444)	1.059 (0.883-1.271)	0.536	56.4	0.011	0.324	0.640															
					Caucasian	0.2947	Allelic	Random	3	3368 (1258/2110)	1.077 (0.905-1.281)	0.405	46.1	0.156	NA	NA															
							Dominant	Random	3	3368 (1258/2110)	1.092 (0.883-1.349)	0.417	29.1	0.244	NA	NA															
							Recessive	Random	3	3368 (1258/2110)	1.095 (0.749-1.599)	0.640	52.8	0.120	NA	NA															
					Asian	0.4510	Allelic	Random	5	3885 (2189/1696)	1.117 (0.908-1.373)	0.296	74.5	0.003	NA	NA															
							Dominant	Random	5	3885 (2189/1696)	1.248 (0.927-1.680)	0.145	70.5	0.009	NA	NA															
							Recessive	Random	5	3885 (2189/1696)	1.041 (0.781-1.388)	0.783	59.7	0.042	NA	NA															
					African	0.4214	Allelic	Random	1	535 (255/280)	1.373 (1.078-1.748)	0.010	NA	NA	NA	NA															
							Dominant	Random	1	535 (255/280)	1.368 (0.942-1.985)	0.1	NA	NA	NA	NA															
							Recessive	Random	1	535 (255/280)	1.714 (1.132-2.596)	0.011	NA	NA	NA	NA															
					Mixed	0.7162	Allelic	Random	1	1651 (611/1040)	0.944 (0.796-1.119)	0.504	NA	NA	NA	NA															
							Dominant	Random	1	1651 (611/1040)	1.053 (0.709-1.563)	0.798	NA	NA	NA	NA															
							Recessive	Random	1	1651 (611/1040)	0.894 (0.718-1.113)	0.318	NA	NA	NA	NA															
					Latino	0.3261	Allelic	Random	1	412 (113/299)	0.781 (0.557-1.096)	0.153	NA	NA	NA	NA															
							Dominant	Random	1	412 (113/299)	0.757 (0.491-1.168)	0.209	NA	NA	NA	NA															
							Recessive	Random	1	412 (113/299)	0.674 (0.313-1.455)	0.315	NA	NA	NA	NA															
65	ERCC5	rs2018836	G vs A	Lung	Caucasian	0.6875	Allelic	Random	2	2544 (895/1649)	1.200 (0.876-1.644)	0.256	66.8	0.083	NA	1.000															
							Dominant	Fixed	2	2544 (895/1649)	1.252 (0.935-1.676)	0.132	0.0	0.586	NA	1.000															
							Recessive	Random	2	2544 (895/1649)	1.253 (0.792-1.981)	0.335	73.7	0.051	NA	1.000															
66	ERCC5	rs2227869	C vs G	Lung	Overall	0.8975	Allelic	Fixed	2	2465 (866/1599)	0.939 (0.702-1.254)	0.669	11.2	0.289	NA	1.000															
							Dominant	Fixed	2	2465 (866/1599)	1.287 (0.589-2.811)	0.527	0.0	0.834	NA	1.000															
							Recessive	Fixed	2	2465 (866/1599)	0.886 (0.644-1.219)	0.458	0.0	0.473	NA	1.000															
					Caucasian	0.9613	Allelic	Fixed	1	2221 (744/1477)	0.881 (0.645-1.202)	0.423	NA	NA	NA	NA															
							Dominant	Fixed	1	2221 (744/1477)	1.010 (0.091-11.151)	0.994	NA	NA	NA	NA															
							Recessive	Fixed	1	2221 (744/1477)	0.874 (0.634-1.204)	0.409	NA	NA	NA	NA															
					Asian	0.0495	Allelic	Fixed	1	244 (122/122)	1.395 (0.633-3.076)	0.409	NA	NA	NA	NA															
							Dominant	Fixed	1	244 (122/122)	1.324 (0.580-3.022)	0.505	NA	NA	NA	NA															
							Recessive	Fixed	1	244 (122/122)	2.847 (0.115-70.618)	0.523	NA	NA	NA	NA															
67	ERCC5	rs2228959	A vs C	Lung	Asian	0.2186	Allelic	Fixed	2	984 (492/492)	0.370 (0.283-0.484)	0.000	61.6	0.107	NA	1.000	Weak	BCA	295	B	C	A	No	NA	NA	NA	NA	1.000	No	0.000	Moderate
							Dominant	Random	2	984 (492/492)	0.429 (0.188-0.979)	0.044	66.9	0.082	NA	1.000	Weak	BCA	259	B	C	A	No	NA	NA	NA	NA	1.000	No	0.852	Weak
							Recessive	NA	2	984 (492/492)	NA	NA	NA	NA	NA	1.000															

68	ERCC5	rs2296147	C vs T	Lung	Caucasian	0.4799	Allelic	Fixed	2	2693 (918/1775)	1.033 (0.923-1.157)	0.572	41.8	0.19	NA	1.000															
							Dominant	Random	2	2693 (918/1775)	0.944 (0.576-1.545)	0.818	77.9	0.033	NA	1.000															
							Recessive	Fixed	2	2693 (918/1775)	1.018 (0.842-1.230)	0.856	0.0	0.986	NA	1.000															
69	ERCC5	rs3759500	T vs C	Lung	Caucasian	0.2321	Allelic	Random	2	2544 (895/1649)	0.811 (0.529-1.244)	0.337	76.8	0.038	NA	1.000															
							Dominant	Random	2	2544 (895/1649)	0.787 (0.467-1.326)	0.368	77.7	0.034	NA	1.000															
							Recessive	Fixed	2	2544 (895/1649)	0.840 (0.580-1.217)	0.356	0.0	0.444	NA	1.000															
70	ERCC5	rs732321	C vs A	Lung	Caucasian	0.0380	Allelic	Random	2	2544 (895/1649)	1.489 (0.740-2.993)	0.264	66.3	0.085	NA	1.000															
							Dominant	Fixed	2	2544 (895/1649)	1.261 (0.940-1.691)	0.122	61.9	0.105	NA	1.000															
							Recessive	Fixed	2	2544 (895/1649)	1.616 (0.265-9849)	0.603	0.0	0.545	NA	1.000															
71	ERCC5	rs873601	A vs G	Lung	Caucasian	0.7286	Allelic	Fixed	2	2693 (918/1775)	1.020 (0.898-1.159)	0.759	0.0	0.566	NA	1.000															
							Dominant	Fixed	2	2693 (918/1775)	1.023 (0.752-1.391)	0.886	0.0	0.645	NA	1.000															
							Recessive	Fixed	2	2693 (918/1775)	1.026 (0.874-1.204)	0.755	0.0	0.334	NA	1.000															
72	ERCC5	rs17655	C vs G	Oral	Asian	0.4433	Allelic	Fixed	2	965 (424/541)	1.334 (1.105-1.611)	0.003	0.0	0.365	NA	1.000	Moderate	BAA	870	B	A	A	No	NA	NA	NA	NA	1.000	No	0.054	Moderate
							Dominant	Fixed	2	965 (424/541)	1.414 (1.059-1.889)	0.019	0.0	0.974	NA	1.000	Moderate	BAA	650	B	A	A	No	NA	NA	NA	NA	1.000	No	0.359	Weak
							Recessive	Fixed	2	965 (424/541)	1.485 (1.082-2.038)	0.014	52.3	0.148	NA	1.000	Weak	BCA	220	B	C	A	No	NA	NA	NA	NA	1.000	No	0.347	Weak
73	ERCC5	rs1047768	C vs T	Prostate	Overall	0.3480	Allelic	Fixed	2	3900 (1724/2176)	1.013 (0.916-1.120)	0.798	0.0	0.438	NA	1.000															
							Dominant	Fixed	2	3900 (1724/2176)	1.002 (0.874-1.148)	0.977	33.6	0.220	NA	1.000															
							Recessive	Fixed	2	3900 (1724/2176)	1.051 (0.857-1.288)	0.634	0.0	0.943	NA	1.000															
					Asian	0.2853	Allelic	Fixed	1	2059 (1004/1055)	1.050 (0.918-1.201)	0.479	NA	NA	NA	NA															
							Dominant	Fixed	1	2059 (1004/1055)	1.071 (0.901-1.273)	0.437	NA	NA	NA	NA															
							Recessive	Fixed	1	2059 (1004/1055)	1.041 (0.755-1.436)	0.806	NA	NA	NA	NA															
					Caucasian	0.4328	Allelic	Fixed	1	1841 (720/1121)	0.969 (0.834-1.127)	0.684	NA	NA	NA	NA															
							Dominant	Fixed	1	1841 (720/1121)	0.898 (0.719-1.121)	0.342	NA	NA	NA	NA															
							Recessive	Fixed	1	1841 (720/1121)	1.057 (0.813-1.374)	0.678	NA	NA	NA	NA															
74	ERCC5	rs17655	C vs G	Prostate	Overall	0.3844	Allelic	Random	4	4755 (2128/2627)	1.087 (0.935-1.264)	0.277	54.4	0.087	0.904	1.000															
							Dominant	Fixed	4	4755 (2128/2627)	1.149 (1.005-1.312)	0.042	24.8	0.263	0.493	1.000	Weak	AAC	2661	A	A	C	HWE, Low OR	No	No	Yes	0.493	1.000	Yes	0.419	Weak
							Recessive	Fixed	4	4755 (2128/2627)	1.058 (0.900-1.244)	0.496	49.8	0.113	0.382	0.308															
					Asian	0.4689	Allelic	Random	2	2359 (1154/1205)	1.122 (0.950-1.326)	0.175	23.7	0.252	NA	NA															
							Dominant	Fixed	2	2359 (1154/1205)	1.176 (0.977-1.414)	0.086	0.0	0.786	NA	NA															
							Recessive	Fixed	2	2359 (1154/1205)	1.101 (0.906-1.337)	0.334	73.3	0.053	NA	NA															
					Caucasian	0.1838	Allelic	Random	1	1841 (720/1121)	1.208 (1.003-1.454)	0.046	NA	NA	NA	NA															
							Dominant	Fixed	1	1841 (720/1121)	1.243 (0.998-1.548)	0.052	NA	NA	NA	NA															
							Recessive	Fixed	1	1841 (720/1121)	1.277 (0.770-2.116)	0.344	NA	NA	NA	NA															
					African	0.5648	Allelic	Random	1	555 (254/301)	0.854 (0.673-1.082)	0.191	NA	NA	NA	NA															
							Dominant	Fixed	1	555 (254/301)	0.788 (0.526-1.179)	0.246	NA	NA	NA	NA															
							Recessive	Fixed	1	555 (254/301)	0.839 (0.584-1.205)	0.341	NA	NA	NA	NA															
75	ERCC5	rs2227869	C vs G	Prostate	Overall	0.0591	Allelic	Fixed	2	2396 (974/1422)	1.143 (0.877-1.488)	0.322	0.8	0.315	NA	1.000															
							Dominant	Fixed	2	2396 (974/1422)	1.127 (0.855-1.485)	0.196	0.0	0.338	NA	1.000															
							Recessive	NA	2	2396 (974/1422)	NA	NA	NA	NA	NA	1.000															
					Caucasian	0.0646	Allelic	Fixed	1	1841 (720/1121)	1.224 (0.911-1.646)	0.180	NA	NA	NA	NA															
							Dominant	Fixed	1	1841 (720/1121)	1.209 (0.886-1.650)	0.231	NA	NA	NA	NA															
							Recessive	NA	1	1841 (720/1121)	NA	NA	NA	NA	NA	NA															
					African	0.0449	Allelic	Fixed	1	555 (254/301)	0.873 (0.483-1.576)	0.652	NA	NA	NA	NA															
							Dominant	Fixed	1	555 (254/301)	0.867 (0.474-1.587)	0.644	NA	NA	NA	NA															
							Recessive	NA	1	555 (254/301)	NA	NA	NA	NA	NA	NA															
76	ERCC5	rs17655	C vs G	Skin	Caucasian	0.3245	Allelic	Fixed	10	11284 (5162/6122)	0.951 (0.890-1.015)	0.133	0.0	0.670	0.521	0.474															
							Dominant	Fixed	10	11284 (5162/6122)	0.950 (0.872-1.036)	0.247	0.0	0.873	0.840	0.592															
							Recessive	Fixed	10	11284 (5162/6122)	0.920 (0.807-1.050)	0.216	20.0	0.259	0.113	0.152															
77	ERCC5	rs1047768	C vs T	Stomach	Asian	0.2994	Allelic	Fixed	3	3818 (1808/2010)	1.055 (0.955-1.166)	0.294	0.0	0.599	0.015	0.296															
							Dominant	Fixed	3	3818 (1808/2010)	1.039 (0.913-1.183)	0.562	0.0	0.568	0.186	0.296															
							Recessive	Fixed	3	3818 (1808/2010)	1.154 (0.930-1.431)	0.193	0.0	0.657	0.333	0.296															
78	ERCC5	rs17655	C vs G	Stomach	Overall	0.5950	Allelic	Random	6	3174 (1372/1802)	0.990 (0.777-1.263)	0.937	73.8	0.002	0.704	1.000															
							Dominant	Random	6	3174 (1372/1802)	0.911 (0.652-1.273)	0.586	48.3	0.085	0.263	0.452															
							Recessive	Random	6	3174 (1372/1802)	1.016 (0.738-1.399)	0.922	67.9	0.008	0.787	0.707															
					Caucasian	0.5808	Allelic	Fixed	2	1493 (643/850)	1.282 (1.024-1.606)	0.030	18.3	0.269	NA	1.000	Strong	AAA	1390	A	A	A	No	NA	NA	NA	NA	1.000	No	0.404	Moderate
							Dominant	Random	2	1493 (643/850)	1.033 (0.668-1.596)	0.885	0.0	0.605	NA	NA															
							Recessive	Random	2	1493 (643/850)	1.513 (1.126-2.034)	0.006	0.0	0.686	NA	1.000	Moderate	BAA	494	B	A	A	No	NA	NA	NA	NA	1.000	No	0.187	Moderate
					Asian	0.6077	Allelic	Random	4	1681 (729/952)	0.916 (0.690-1.215)	0.542	74.7	0.008	NA	NA															
							Dominant	Random	4	1681 (729/952)	0.844 (0.507-1.404)	0.513	67.5	0.026	NA	NA															
							Recessive	Random	4	1681 (729/952)	0.887 (0.656-1.199)	0.435	52.9	0.095	NA	NA															
79	ERCC5	rs2094258	T vs C	Stomach	Asian	0.3885	Allelic	Random	9	9884 (4648/5236)	1.022 (0.929-1.124)	0.652	54.9	0.023	0.740	0.754															
							Dominant	Fixed	9	9884 (4648/5236)	0.999 (0.920-1.086)	0.990	38.5	0.112	0.969	0.917															
							Recessive	Random	9	9884 (4648/5236)	1.065 (0.912-1.244)	0.428	41.1	0.093	0.341	0.251															
80	ERCC5	rs2296147	C vs T	Stomach	Asian	0.2121	Allelic	Fixed	5	7589 (3699/3890)	0.989 (0.915-1.069)	0.778	33.7	0.197	0.530	0.806															
							Dominant	Fixed	5	7589 (3699/3890)	0.964 (0.879-1.058)	0.444	0.0	0.697	0.332	0.462															
							Recessive	Random	5	7589 (3699/3890)	1.130 (0.782-1.632)	0.517	64.2	0.025	0.685	0.806															
81	ERCC5	rs751402	G vs A	Stomach	Asian	0.6558	Allelic	Random	10	8565 (3989/4576)	0.865 (0.784-0.954)	0.004	51.8	0.028	0.022	0.152	Weak	ACA	10864	A	C	A	No	None	No	No	0.022	0.152	No	0.044	Moderate
81	ERCC5	rs751402	G vs A	Stomach	Asian	0.6558	Dominant	Random	10	8565 (3989/4576)	0.802 (0.657-0.980)	0.031	50.4	0.034	0.015	0.152	Weak	ACC	7362	A	C	C	HWE	None	No	Yes	0.015	0.152	No	0.379	Weak
81	ERCC5	rs751402	G vs A	Stomach	Asian	0.6558	Recessive	Fixed	10	8565 (3989/4576)	0.867 (0.794-0.946)	0.001	6.7	0.380	0.031	0.107	Strong	AAA	3502	A	A	A	No	None	No	No	0.031	0.107	No	0.010	Strong
82	ERCC5	rs873601	A vs G	Stomach	Asian	0.4757	Allelic	Fixed	6	8717 (4177/4540)	1.069 (1.007-1.135)	0.029	29.2	0.216	0.150	0.133	Weak	ABC	4177	A	B	C	First study; Low OR	Yes	No	None	0.150	0.133	Yes	0.444	Weak
							Dominant	Fixed	6	8717 (4177/4540)	1.056 (0.960-1.161)	0.264	0.5	0.413	0.448	0.707															
							Recessive	Fixed	6	8717 (4177/4540)	1.133 (1.026-1.251)	0.014	22.5	0.265	0.053	0.133	Weak	AAC	8630	A	A	C	First study; Low OR	Yes	No	None	0.053	0.133	Yes	0.195	Weak
83	ERCC5	rs17655	C vs G	Thyroid	Caucasian	0.5551	Allelic	Fixed	2	647 (181/466)	0.782 (0.595-1.028)	0.078	15.4	0.277	NA	1.000															
							Dominant	Fixed	2	647 (181/466)	1.005 (0.654-1.545)	0.982	0.0	0.567	NA	1.000															
							Recessive	Fixed	2	647 (181/466)	0.501 (0.313-0.801)	0.004	0.0	0.491	NA	1.000	Moderate	BAA	207	B	A	A	No	NA	NA	NA	NA	1.000	No	0.388	Weak
84	ERCC5	rs2227869	C vs G	Thyroid	Caucasian	0.1655	Allelic	Random	2	882 (441/441)	0.849 (0.432-1.668)	0.634	64.3	0.094	NA	1.000															
							Dominant	Fixed	2	882 (441/441)	0.897 (0.662-1.217)	0.486	62.7	0.102	NA	1.000															
							Recessive	Fixed	2	882 (441/441)	2.304 (0.978-5.429)	0.056	0.0	0.921	NA	1.000															
85	ERCC5	rs17655	C vs G	Uterus	Overall	0.3544	Allelic	Random	5	4431 (1936/2495)	1.239 (1.050-1.463)	0.011	63.5	0.027	0.707	0.806	Weak	ACA	1856	A	C	A	No	No	No	No	0.707	0.806	No	0.181	Weak
							Dominant	Random	5	4431 (1936/2495)	1.315 (1.039-1.664)	0.023	63.5	0.027	0.234	0.806	Weak	ACC	2414	A	C	C	HWE	No	No	Yes	0.234	0.806	No	0.332	Weak
85	ERCC5	rs17655	C vs G	Uterus	Overall	0.3544	Recessive	Fixed	5	4431 (1936/2495)	1.355 (1.142-1.608)	0.001	0.0	0.482	0.417	1.000	Strong	AAA	4270	A	A	A	No	No	No	No	0.417	1.000	No	0.011	Strong
85	ERCC5	rs17655	C vs G	Cervical Cancer	Asian	0.4764	Allelic	Random	2	1800 (678/1122)	1.365 (1.190-1.565)	0.000	0.0	0.483	NA	1.000	Strong	AAA	1829	A	A	A	No	NA	NA	NA	NA	1.000	No	0.000	Strong
85	ERCC5	rs17655	C vs G	Cervical Cancer	Asian	0.4764	Dominant	Random	2	1800 (678/1122)	1.618 (1.286-2.035)	0.000	0.0	0.820	NA	1.000	Strong	AAA	1335	A	A	A	No	NA	NA	NA	NA	1.000	No	0.003	Strong
							Recessive	Fixed	2	1800 (678/1122)	1.387 (1.121-1.715)	0.003	43.6	0.183	NA	1.000	Moderate	BBA	494	B	B	A	No	NA	NA	NA	NA	1.000	No	0.059	Moderate
				Endometrial Cancer	Caucasian	0.2485	Allelic	Random	3	2631 (1258/1373)	1.179 (0.923-1.505)	0.187	66.7	0.050	NA	NA															
							Dominant	Random	3	2631 (1258/1373)	1.123 (0.899-1.402)	0.307	38.1	0.199	NA	NA															
							Recessive	Fixed	3	2631 (1258/1373)	1.299 (0.974-1.734)	0.075	0.0	0.456	NA	NA															
86	ERCC5	rs2094258	T vs C	Head and Neck	Overall	0.1926	Allelic	Fixed	2	2667 (1330/1337)	1.051(0.916-1.204)	0.479	16.0	0.275	NA	1.000															
							Dominant	Fixed	2	2667 (1330/1337)	1.059 (0.901-1.244)	0.489	0.0	0.423	NA	1.000															
							Recessive	Fixed	2	2667 (1330/1337)	1.064 (0.738-1.534)	0.740	21.9	0.258	NA	1.000															
					Asian	0.8255	Allelic	Fixed	1	542 (271/271)	1.195 (0.913-1.563)	0.194	NA	NA	NA	NA															
							Dominant	Fixed	1	542 (271/271)	1.195 (0.852-1.676)	0.301	NA	NA	NA	NA															
							Recessive	Fixed	1	542 (271/271)	1.428 (0.760-2.684)	0.268	NA	NA	NA	NA															
					Caucasian	0.0920	Allelic	Fixed	1	2125 (1059/1066)	1.004 (0.857-1.177)	0.957	NA	NA	NA	NA															
							Dominant	Fixed	1	2125 (1059/1066)	1.021 (0.849-1.228)	0.823	NA	NA	NA	NA															
							Recessive	Fixed	1	2125 (1059/1066)	0.912 (0.580-1.435)	0.691	NA	NA	NA	NA															

Abbreviations:OR, odds ratio; A, adenine; T, thymine; G, guanine; C, cytosine; ERCC: excision repair cross-complementation.

First study=Yes: Significance lost excluding the first published or first positive report; No: Significance was not lost excluding the first published or first positive report; None: A meta-analysis didn't include studies with the first published or first positive report.

HWE:=Yes: Significance lost excluding studies with controls violating HWE; No: Significance was not lost excluding the study with controls violating HWE; None: A meta-analysis didn't include studies with controls violating HWE.

Low OR=Low magnitude of the association (i.e. 0.87<OR<1.15)

Publication bias= Possible existence of potential publication bias (Begg's test, p<0.10)

Small study (Sample size<100) = Possible existence of potential small study effect (modified Egger's test, p<0.10).

aMinor alleles vs major alleles (reference).

bFrequency of minor allele in controls.

cStrength of epidemiological evidence based on the Venice criteria.

dThe prior probability of FPRP is 0.05, and the FPRP level of noteworthiness is 0.20.

eDegree of epidemiological credibility based on the combination of results from Venice guidelines and FPRP tests.
