# Supplementary material for: Polymorphisms in ERCC4 and ERCC5 and risk of cancers: Systematic research synopsis, meta-analysis, and epidemiological evidence
Source: Front Oncol. 2022 Aug 11;12:951193. doi: 10.3389/fonc.2022.951193 (PMC9404303; doi:10.3389/fonc.2022.951193)
Supplement: Supplementary file 9 [file Table_5.pdf]

Supplementary Table S5. Characteristics of the articles related to 4 SNPs with strong evidence of a significant cancer correlation

PMID/Ref	First author, Year	cancer site	Genotyping	Country/ region	ethnicity	Study design	Case	control	sample size	Gene	variant
20062074	Wang,M, 2010	Bladder	PCR-RFLP	China	Asian	HBCCS	234	250	484	ERCC4	rs744154 (G>C)
20062074	Wang,M, 2010	Bladder	PCR-RFLP	China	Asian	HBCCS	130	150	280	ERCC4	rs744154 (G>C)
28952217	Zhang,C, 2017	Esophagus	TaqMan	China	Asian	HBCCS	557	1503	2060	ERCC5	rs2296147 (T>C)
22848513	Zhu,M.L, 2012	Esophagus	TaqMan	China	Asian	HBCCS	1115	1117	2232	ERCC5	rs2296147 (T>C)
24582975	Lu,B, 2014	Laryngeal	MassARRAY	China	Asian	HBCCS	176	176	352	ERCC5	rs17655 (G>C)
24563277	Li,X, 2014	Laryngeal	MassARRAY	China	Asian	HBCCS	211	210	421	ERCC5	rs17655 (G>C)
19444904	Abbasi,R, 2009	Laryngeal	PCR-RFLP	Germany	Caucasian	PBCCS	248	647	895	ERCC5	rs17655 (G>C)
26130668	Joo,J, 2016	Uterus	TaqMan	Korea	Asian	HBCCS	478	922	1400	ERCC5	rs17655 (G>C)
22271435	Cincin,Z.B, 2012	Uterus	PCR-RFLP	Turkey	Caucasian	HBCCS	104	158	262	ERCC5	rs17655 (G>C)
21750170	Doherty,J.A, 2011	Uterus	SNaPshot	USA	Caucasian	PBCCS	703	714	1417	ERCC5	rs17655 (G>C)
19096231	He,X 2008	Uterus	TaqMan	China	Asian	CCS	200	200	400	ERCC5	rs17655 (G>C)
16284373	Weiss,J.M, 2005	Uterus	SNaPshot	USA	Caucasian	PBCCS	371	420	791	ERCC5	rs17655 (G>C)
29434449	Sang,L, 2018	Stomach	MassARRAY	China	Asian	CCS	427	509	936	ERCC5	rs751402 (A>G)
27929383	Hua,R.X ,2016	Stomach	TaqMan	China	Asian	CCS	1142	1173	2315	ERCC5	rs751402 (A>G)
27706622	Yang,L.Q, 2016	Stomach	PCR-RFLP	China	Asian	HBCCS	155	246	401	ERCC5	rs751402 (A>G)
27323183	Guo,B.W, 2016	Stomach	PCR-RFLP	China	Asian	HBCCS	142	274	416	ERCC5	rs751402 (A>G)
27323165	Feng,Y.B, 2016	Stomach	PCR-RFLP	China	Asian	HBCCS	177	236	413	ERCC5	rs751402 (A>G)
27323158	Lu,J.J, 2016	Stomach	PCR-RFLP	China	Asian	HBCCS	184	206	390	ERCC5	rs751402 (A>G)
27228234	Zhou,R.M, 2016	Stomach	PCR-LDR	China	Asian	CCS	431	432	863	ERCC5	rs751402 (A>G)
27051028	Li,R.J ,2016	Stomach	PCR-RFLP	China	Asian	HBCCS	216	216	432	ERCC5	rs751402 (A>G)
26820236	Chen,Y.Z 2016	Stomach	TaqMan	China	Asian	HBCCS	692	771	1463	ERCC5	rs751402 (A>G)
22982416	Duan,Z, 2012	Stomach	PCR-RFLP	China	Asian	PBCCS	400	400	800	ERCC5	rs751402 (A>G)

Abbreviations: OR, odds ratio; A, adenine; C, cytosine; G, guanine; T, thymine; HBCCS: Hospital-based case control study; CCS: Case-control study; PBCCS: Population-based case control study; ERCC: excision repair cross-complementation; PCR-LDR: polymerase chain reaction/ligase detection reaction; PCR-RFLP: Polymerase Chain Reaction restriction fragment length polymorphism; ERCC: excision repair cross-complementation; RT-PCR: Real time Polymerase Chain Reaction restriction.
