# Supplementary material for: Polymorphisms in ERCC4 and ERCC5 and risk of cancers: Systematic research synopsis, meta-analysis, and epidemiological evidence
Source: Front Oncol. 2022 Aug 11;12:951193. doi: 10.3389/fonc.2022.951193 (PMC9404303; doi:10.3389/fonc.2022.951193)
Supplement: Supplementary file 10 [file Table_6.pdf]

Supplementary Table S6. Analyses of expression quantitative trait locus (eQTL) in significant variants associated with risk of cancer

Variant	Gene	Tissue	*P* value	Effect Size
rs744154	ERCC4	Muscle - Skeletal	1.4×10^{-8}	-0.15
	MKL2	Colon - Transverse	8.2×10^{-05}	-0.13
rs2296147	BIVM	Esophagus - Mucosa	1.2×10^{-16}	-0.2
	BIVM	Esophagus - Muscularis	4.1×10^{-06}	-0.12
	BIVM	Esophagus - Gastroesophageal Junction	7.3×10^{-5}	-0.12
	METTL21EP	Esophagus - Mucosa	4.3×10^{-08}	0.32
	METTL21EP	Esophagus - Muscularis	6.5×10^{-06}	0.3
rs751402	BIVM	Breast - Mammary Tissue	4.3×10^{-06}	-0.23
	ERCC5	Esophagus - Mucosa	1.1×10^{-05}	-0.13
	RP11-255P5.3	Nerve - Tibial	9.2×10^{-05}	-0.24
rs17655	ERCC5	Muscle - Skeletal	4.7×10^{-05}	0.19

Data source: Genotype-Tissue Expression (GTEx) Project
