# Supplementary material for: Polymorphisms in ERCC4 and ERCC5 and risk of cancers: Systematic research synopsis, meta-analysis, and epidemiological evidence
Source: Front Oncol. 2022 Aug 11;12:951193. doi: 10.3389/fonc.2022.951193 (PMC9404303; doi:10.3389/fonc.2022.951193)
Supplement: Supplementary file 11 [file Table_7.pdf]

Supplementary Table S7. Linkage Disequilibrium (r^2) among the significant variants in the ERCC5 gene

SNP Pair[1]	Asian[2]	European[2]	African[2]
rs2296147 & rs17655	0.2366	0.0739	0.1354
rs2296147 & rs751402	0.1470	0.2125	0.0883
rs17655 & rs751402	0.2450	0.0679	0.1948

[1]Data source: ldlink.nci.nih.gov
[2]Asians, European, and African were selected from five major population categories provided by the databases.
